# Network motif analysis of a multi-mode genetic-interaction network

**DOI:** 10.1186/gb-2007-8-8-r160

**Published:** 2007-08-02

**Authors:** R James Taylor, Andrew F Siegel, Timothy Galitski

**Affiliations:** 1 Institute for Systems Biology, N. 34^th ^Street, Seattle, WA 98103 USA; 2 University of British Columbia, Department of Genetics, Vancouver, BC, V6T 1Z4, Canada; 3 University of Washington, Departments of Management Science, Finance, and Statistics, Seattle, WA, 98195, USA

## Abstract

Different modes of genetic interaction indicate different functional relationships between genes. The extraction of biological information from dense multi-mode genetic-interaction networks demands appropriate statistical and computational methods. We developed such methods and implemented them in open-source software. Motifs extracted from multi-mode genetic-interaction networks form functional subnetworks, highlight genes dominating these subnetworks, and reveal genetic reflections of the underlying biochemical system.

## Abstract 2

Statistical and computational methods for the extraction of biological information from dense multi-mode genetic-interaction networks were developed and implemented in open-source software.

## Background

The cell is an elaborate network of biomolecular and environmental interactions that together bring about complex phenotypes. Understanding the functional consequences of molecular interactions is fundamental to understanding phenotypes. A highly successful approach is the use of genetic interactions. Genetic interactions describe the phenotypic consequences of combinations of genetic perturbations. Genetic interactions combined with molecular interaction data can delineate information flows through complex biochemical systems. The concept of the molecular signaling pathway owes much to this approach.

A genetic interaction comprises phenotype measurements of four genotypes: the reference genotype (wild type (WT)); a single gene perturbation A; a perturbation B of a different gene; and the double perturbation AB. By themselves, the single perturbations link individual genes to specific phenotypes and biological processes. Studying a double perturbation defines functional relationships between the perturbed genes. The relative ordering of the four phenotype measurements defines different genetic-interaction modes [1]. Genetic-interaction modes indicate one or more possible molecular relationships, for example, upstream/downstream. Networks of genetic interaction, and the molecular wiring, constrain these possibilities. In this way, genetic-interaction modes are a reflection of the underlying biochemical system.

Geneticists have formalized collections of genetic interactions into genetic-interaction networks of perturbed-gene nodes and genetic-interaction edges. Tong *et al*. [2] created a network consisting of edges representing a single type of genetic interaction, synthetic lethal. Zhang *et al*. [3] integrated this network with disparate data types, including protein-protein and protein-DNA interactions, sequence homologies, and expression correlations. In this study, network patterns were used to reduce the overall system into a thematic map of biological relationships. The E-MAP method [4,5] creates high-density genetic-interaction networks consisting of aggravating or alleviating edge types. This method has been fruitful for identifying both system-level and protein-complex-level functional modularity.

Further work has generated networks of multiple genetic-interaction modes (edge types). In Drees *et al*. [1], all possible genetic interactions were classified into nine modes, of which four are asymmetric (directed edges). A multi-mode genetic-interaction network was derived from a large set of quantitative phenotype data. This work revealed local and global genetic-interaction patterns suggesting the prevalence of information contained in the structure and distribution of genetic interactions within the network. Further network information can be extracted from such complex networks by identifying significantly repeated genetic-interaction patterns, network motifs [6-8]. In this study, we report a network-motif analysis of the dense multi-mode genetic-interaction network of Drees *et al*. [1].

## Results and discussion

### Multi-mode genetic-interaction network

In the network of Drees *et al*. [1], there are 1,760 genetic interactions among 128 perturbed genes controlling the agar-invasion phenotype of diploid budding yeast. The perturbations included gene deletions as well as overexpressers and dominant alleles. This yeast-invasiveness network contains all nine possible genetic-interaction modes, including noninteracting, epistatic, synthetic, suppressive, additive, conditional, asynthetic, nonmonotonic, and double-nonmonotonic interaction. Four of these modes (epistatic, suppressive, conditional, and nonmonotonic) are directional, giving thirteen possible edges between any pair of nodes. Note that the genetic-interaction modes discussed in this paper refer to those defined in Drees *et al*. [1], and that there are semantic differences between the Drees definitions and other genetic-interaction classifications. Example interactions for each mode are shown in Additional data file 22.

### Genetic-interaction patterns reflect the underlying molecular system

**Figure 1:**
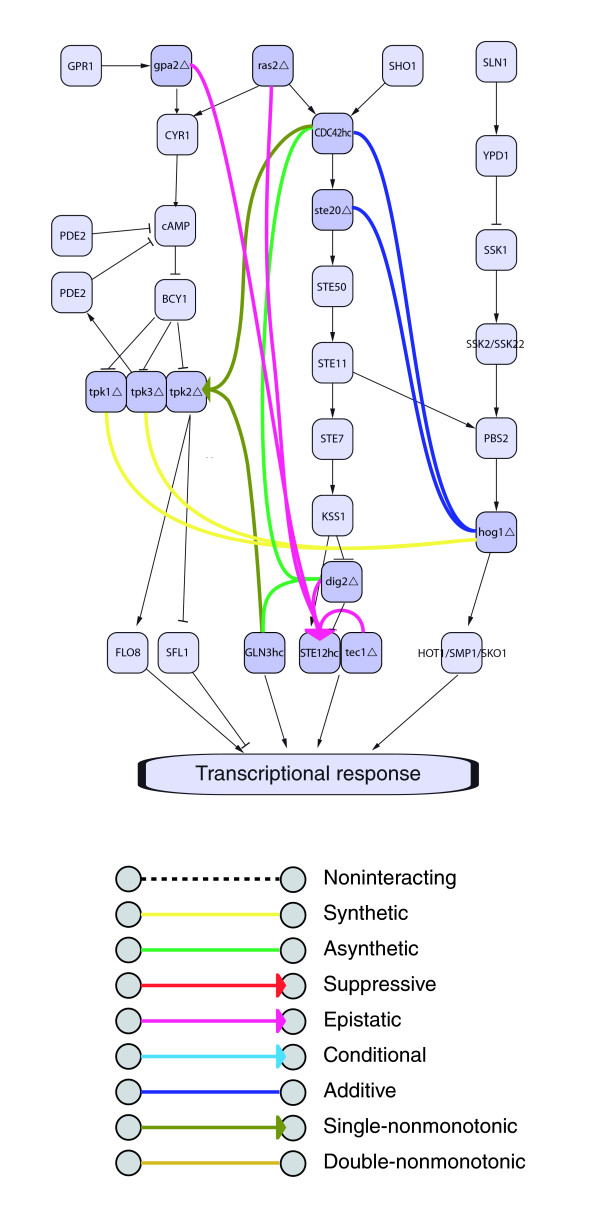
Multi-mode genetic-interaction motifs and the underlying molecular system. Genetic-interaction edges are superimposed onto a diagram of the cAMP, fMAPK, and HogMAPK signaling pathways. Gene perturbations are marked: hc, high copy overexpresser; Δ, deletion.

Prior to rigorous statistical motif analysis, we inspected the yeast-invasiveness network to discern possible patterns of genetic interactions reflecting the underlying molecular system. Figure 1 shows genetic interactions among components of three main signaling pathways controlling yeast invasiveness [9-23]. Subsequently, we investigated our preliminary observations (described below) quantitatively and globally in the network.

We initially observed that there are local patterns incorporating both edge type and network topology. For example, consider the interactions between the overexpressers of *CDC42 *and *GLN3 *and the deletions of *DIG2 *and *TPK2*. Both *CDC42 *and *GLN3 *interact asynthetically with *DIG2 *and nonmonotonically with *TPK2*, creating a two-mode bi-fan interaction pattern.

Also, we observed that patterns of genetic interaction can reflect the direction of information flow through the molecular network. For instance, epistatic interactions involving the *STE12 *overexpresser originate from upstream signaling components. Also, many genetic interaction modes occur repeatedly between parallel information paths. For instance, the *HOG1 *deletion interacts synthetically with deleted components of the cAMP pathway and additively with overexpressed components of the filamentation/invasion MAP-kinase (fMAPK) pathway.

### Statistical model of a null hypothesis

Biologically relevant genetic-interaction patterns can be identified by finding those occurring more frequently in the genetic network than expected at random. This can be done by comparing the number of times a given pattern occurs in the genetic network to the number of times it occurs in a set of properly randomized networks. The randomized networks represent a statistical null hypothesis and effectively model the level of pattern noise in the network [7,24]. In this way, significance can be assigned to each identified pattern. In this study we highlight those patterns with a significance level of *p *< 0.05/*n*, using the Bonferroni multiple-hypothesis-testing correction, where *n *is the number of patterns tested in each analysis. Algorithms were developed to create the set of randomized networks modeling a null hypothesis. The yeast-invasiveness network contains nine edge types of which four are directed. Randomized networks were generated by a Monte Carlo method iteratively selecting a pair of edges at random and swapping their edge types. See Materials and methods for details.

Randomizations were subject to specific constraints to preclude the introduction of biases to the results. Each edge represents the results of a given experiment (repeated measurement of the phenotypes of WT, A, B, and AB). Every genetic experiment creates a resulting genetic edge, with noninteracting edge types used in the cases of genetically noninteracting loci. This causes the topology of the network (the simple presence or absence of an edge of any type linking each pair of nodes) to be determined by experimental design (the set of experiments performed or not performed), not by genetics. Thus, for proper randomization the network topology is held constant. The results could also be biased by the selection of mutant alleles included in the experiments. As described in Additional data file 22, the data for a genetic interaction consist of the ordering of four phenotypes: WT, A, B, and AB. The single-mutant phenotypes could be biased by the selection of mutant alleles. To preclude this allele-selection bias, in our Monte Carlo switching we restricted edge-type swaps to those in which the two edges have the same relative ordering of A, B, and WT. Lastly, in some of the analyses below, molecular data are mapped onto the genetic network. In these cases the genetic-interaction edge types are randomized under the above constraints, while the molecular data are held constant. Note that our randomization methods are strictly conservative and restrict the number of significant motifs. Such methods are necessary to ensure that the calculated significance is due to biological significance rather than experimental design.

### Genetic-interaction network motifs

To identify genetic-interaction network patterns that reflect biological relationships such as those illustrated in Figure 1, we identified network motifs. Network motifs are small repeatedly occurring multi-element components of a network, where the repetition suggests functional significance. Such methods have been successful in extracting information from various other network types [6-8,25,26], as well as identifying general themes in the evolved organization of molecular systems [3].

**Figure 2:**
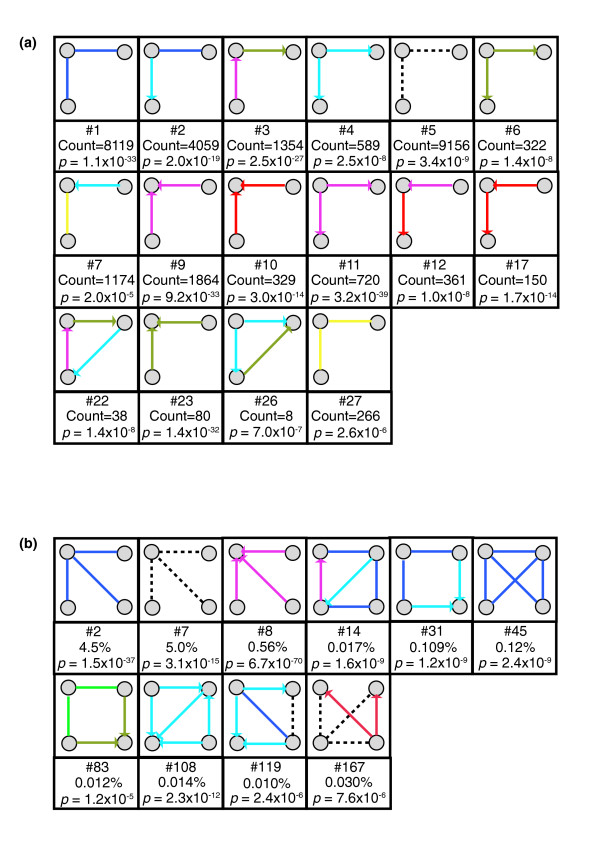
Motifs in the yeast-invasiveness genetic-interaction network. **(a) **Examples of significant 3-node motifs. The number of instances of each motif is indicated as is the *p *value. A statistical cutoff of *p *= 0.05/489 = 1.02 × 10^-4 ^was used to define significant patterns. **(b) **Examples of significant 4-node motifs. The number of occurrences is shown as the percentage of the full number of patterns sampled. *P *values are shown and a statistical cutoff of *p *= 0.05/1,505 = 3.32 × 10^-5 ^was used to define significant patterns. The full collection of motifs is in Additional data files 1 and 4.

The simplest network patterns containing information about the genetic-interaction modes and their system-level organization are 3-node motifs (3n-motifs). Using the null hypothesis method described above, we enumerated all 3n patterns in the yeast invasiveness network and tested each one for biological significance. We found 27 significant motifs among the 489 different patterns observed in the network (5.5%). Many of these motifs occur hundreds or thousands of times in the yeast-invasion network. Examples are shown in Figure 2a. The full set is found in Additional data file 1. Homogeneous-edge-type motifs were found frequently, with 9 of the 13 possible homogeneous 2-edge patterns being significant (3n-motifs 1, 4, 5, 6, 9, 10, 11, 23, 27). Examples of such motifs occur in Figure 1. Their global frequency may reflect the tendency of gene perturbations to show 'monochromatic' interaction [1,27]. Many heterogeneous motifs also were found (3n-motifs 2, 3, 7, 8, 12, and so on), as were various fully connected motifs (for example, 3n-motifs 22, 24, 25, 26, and so on).

We also identified significant 4-node patterns (4n-motifs). Because the number of pattern instances contained in a network scales combinatorially with local network density and pattern order (number of nodes in the pattern), the full enumeration of 4n pattern instances was computationally infeasible. Thus, a sampling algorithm (Materials and methods) [28] was employed. Of the 1,505 4n patterns sampled from the original network, 190 (12.6%) were repeated significantly. The full list of 4n-motifs can be found in Additional data file 4. Figure 2b shows examples. We found 4n-motifs exhibiting the edge-type homogeneity detected among 3n-motifs, as well as mixed-edge-type motifs.

**Figure 3:**
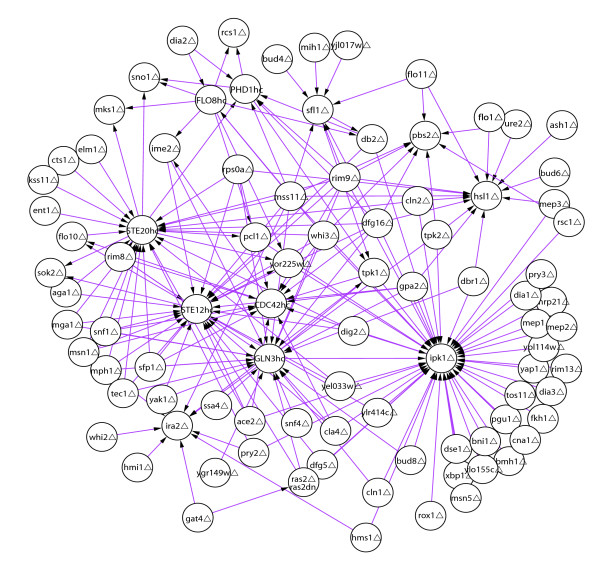
Motif subnetworks. An example of a motif subnetwork. A motif subnetwork is the union of all instances of a specific motif. Shown here is the subnetwork of 3n-motif 9. The gene perturbations comprising the genetic interactions are marked with the suffixes: hc, high copy overexpresser; Δ, deletion.

We noted that specific nodes (gene perturbations) often appear repeatedly among the numerous instances of a specific motif. This suggested that the instances of motifs are connected structural units of larger single-motif subnetworks. Such subnetworks can highlight the main perturbations contributing to a motif, and show the large scale organization of instances of the motif. Figure 3 shows an example of single-motif subnetworks, and additional examples are in Additional data file 23. In Figure 3 is the incoming epistatic motif network of 3n-motif 9. In an epistatic interaction, the phenotype of the double mutant is the same as one of the two gene perturbations, and depending on the allele type (hypermorphic or hypomorphic), orders the epistatic gene upstream or downstream (see mode definitions in Drees *et al*. [1]). In this way, epistatic interactions have been commonly used to help identify and delineate directed information flows in biochemical systems. As shown in Figure 3, the epistatic motif network is organized around six main gene perturbation hubs: the overexpressions of *STE20*, *STE12*, *CDC42 *and *GLN3*, and the deletions of *IPK1 and HSL1*. Extending the concept of single epistatic interactions, these repeated interactions suggest critical hubs of information flow, and genes whose influences are likely to flow through them.

### Molecular information and genetic-interaction network motifs

Figure 1 illustrates genetic-interaction patterns describing specific functional relationships within and between the signaling pathways. To identify significant relationships between genetic interactions and molecular-function data, we integrated these data types [1-5,29-32]. Patterns from such integrated networks can be tested for statistical significance allowing for the identification of significant network motifs. In our case, these motifs are genetic-interaction patterns that exhibit significance in the context of the molecular system [2].

Filamentation/invasion signaling is a directed system that can be characterized loosely by the molecular functions of the system components. Plasma-membrane receptors transfer information to cytoplasmic signaling components that then regulate nuclear transcription factors. These molecular functions capture a first approximation of the directionality of the system. By mapping the GoSlim [33] 'molecular function' annotations onto the nodes of the yeast-invasiveness network, we identified genetic-interaction network motifs involving these loosely directed relationships.

**Figure 4:**
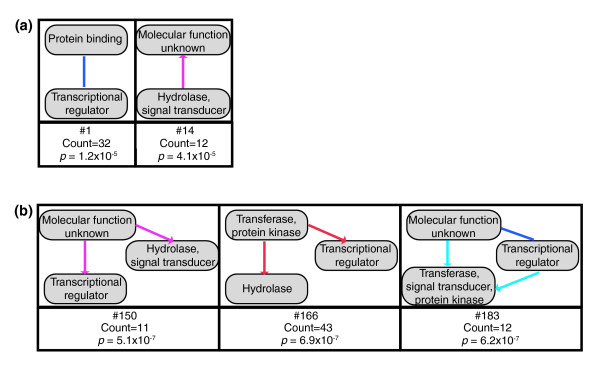
Examples of motifs integrating gene annotations. Examples of significant **(a) **2-node and **(b) **3-node motifs involve genetic-interaction edges and GOSlim molecular-function gene-annotation nodes. The number of instances and calculated *p *value of each motif is indicated. For the 2nGO-motifs a statistical cutoff of *p *= 0.05/575 = 8.7 × 10^-5 ^was used. For the 3nGO-motifs a statistical cutoff of *p *= 0.05/23,286 = 2.14 × 10^-6 ^was used. The full collection of motifs is in Additional data files 7 and 10.

Figure 4a,b shows examples of the significant 2-node and 3-node motifs for the molecular-function annotations, respectively. The full sets are found in Additional data files 7 and 10, respectively. Of the 575 observed 2-node GoSlim molecular function patterns in the original network, 6 (1.0%) were found significant (2nGO-motifs). Of the 23,286 observed 3-node molecular-function patterns, 116 (<0.5%) were found significant (3nGO-motifs). These significant patterns illustrate a correspondence between the genetic-interaction modes and the underlying biochemical system. For example, 2nGO-motif 1 (Figure 4a) shows additive interactions between perturbations of protein-binding proteins and transcriptional regulators. Among the instances of this motif are additive interactions of a deletion of *DIG2 *with overexpression of *FLO8 *and deletion of *SFL1*. The Dig2 protein binds and inhibits the Ste12 protein, a transcriptional activator of the filamentation/invasion MAP-kinase (fMAPK) pathway. *DIG2 *deletion interacts additively with perturbations of *FLO8 *and *SFL1*, encoding transcription factors of a different filamentation/invasion-promoting pathway, the cyclic-AMP pathway. The additive interaction reflects the separate contributions of these pathways. As another example, 3nGO-motif 166 (Figure 4b) shows perturbations of protein kinase/transferase activity proteins interacting supressively to transcriptional regulator proteins and to hydrolase activity proteins. In the context of filamentation signaling, environmental signals are transmitted through hydrolase (for example, GTPase) and kinase activity proteins to transcriptional regulators. In a suppressive genetic interaction, a suppressor gene perturbation ameliorates the effects of the suppressed perturbation, indicating the suppressor perturbation reverses or short-circuits the suppressed perturbation. A specific instance of this is that a deletion of the cAMP-dependent protein kinase subunit Tpk3 abrogates the effects of overexpression of both the membrane localized hydrolase Cdc42 and the transcriptional regulator Ste12. Cdc42 is an upstream activator of the fMAPK signaling pathway, and Ste12 is a downstream transcription factor of the same pathway [9,10,34,35]. This motif instance suggests that loss of *TPK3 *activity in the parallel cAMP pathway offsets the effects of overexpression of *CDC42 *or *STE12 *activity in the fMAPK pathway.

**Figure 5:**
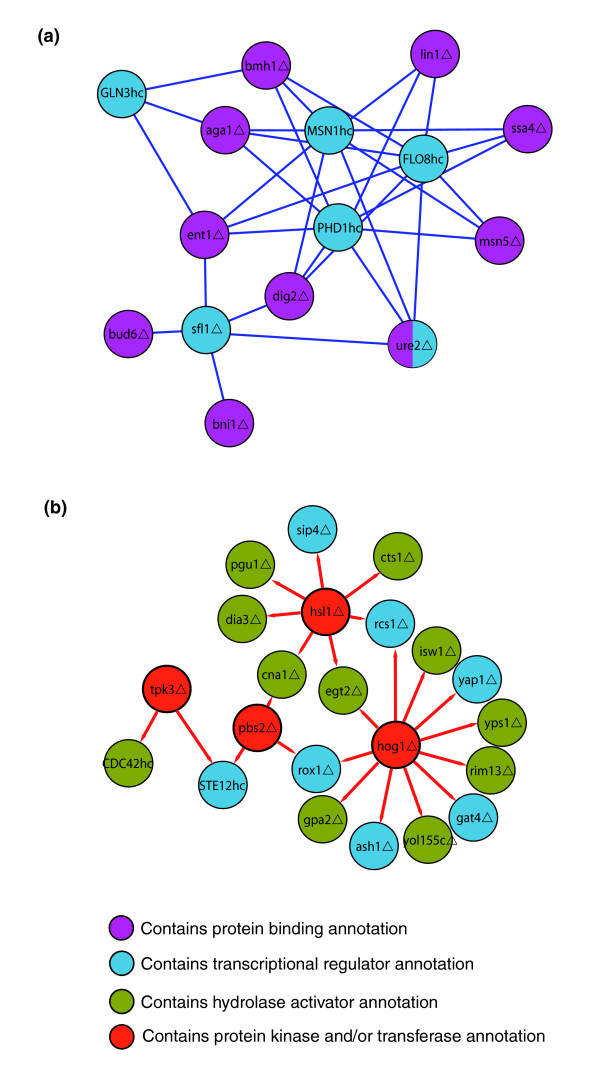
Annotation-motif subnetworks. **(a) **The union of all instances of 2nGO-motif 1, which comprises perturbations of protein binding proteins and transcriptional regulators acting additively. **(b) **The union of all instances of 3nGO-motif 166, which comprises perturbations of protein kinase/transferase activity proteins interacting supressively to transcriptional regulator proteins and to hydrolase activity proteins. Gene perturbations are marked: hc, high copy overexpresser; Δ, deletion.

To investigate the distribution of these motif examples within the full network, motif subnetworks were generated. Figure 5a,b shows the motif subnetworks for 2nGO-motif 1 and 3nGo-motif 166, respectively. The 2nGo-motif 1 network is organized around the transcription factor tri-hub *MSN1*, *PHD1*, and *FLO8*, and the two separate single transcription factor hubs, *SFL1 *and *GLN3*. This network exhibits a high degree of mutually informative genetic interactions. Each of the eight protein binding proteins that interact with the tri-hub (AGA1, BMH1, LIN1, SSA4, MSN5, URE2, DIG2, and ENT1) interacts with each tri-hub member. This suggests overlapping pathway functionality within the set of protein binding proteins and within the set of transcription factors. This motif-instance organization contrasts with that of 3nGo-motif 166. The 3nGo-motif 166 subnetwork centers on the single protein kinase/transferase hubs *TPK3*, *PBS2*, *HOG1*, and *HSL1*. These kinases are information flow constriction points in their respective signaling pathways: *TPK3 *in the cAMP pathway, *PBS2 *and *HOG1 *in the osmolarity sensing pathway, and *HSL1 *in the morphogenic checkpoint pathway. In contrast to the 2nGo-motif network, these single hubs primarily act independently of each other, with two hubs having at most only two nodes in common. This likely reflects the differing roles these pathways play in the invasion phenotype. Interestingly, the osmolarity sensing kinases Pbs2 and Hog1 show differing interaction patterns, although they are implicated in the same pathway. This possibly reflects subtly differing roles of the two kinases. These examples illustrate how the aggregation of motif information in motif subnetworks highlights biological information not present in individual motif instances.

### Comparing network patterns in a similar genetic-interaction network

The diversity of networks that can be formed from 13 edge types and large numbers of nodes is enormous. Thus, the yeast-invasiveness genetic-interaction network probably contains a sample of biologically relevant genetic-interaction motifs. To gauge the scope of our analysis we made a comparison of motifs in the yeast invasiveness network (derived from yeast diploid strains) to a similar network, a yeast diploid agar-adhesion network. The adhesion network was created in parallel to the invasion network reported in Drees *et al*. [1] (data not shown), and although the two phenotypes are related, many genetic interactions differed between the two (652 of 1,751 (37.2%)). To compare the networks, we enumerated their 3-node motifs. For consistency, we pruned the networks such that they had exactly the same topological set of nodes (128) and edges (1,751). We found 27 motifs in both the invasion network and the adhesion network out of 419 and 414 candidate patterns (6.4% and 6.5%, respectively). Of these 27 motifs, 20 (74%) were common to both. This indicates that although common genetic-interaction motifs exist in the two networks, each genetic network also contains a unique subset. The fact that these are related phenotypes underscores this observation.

To further understand the different motif sample spaces of the two networks, we compared the null hypotheses generated by the invasion and adhesion networks. Using the 378 3n patterns common to both networks, we compared the mean number of times each pattern occurred in the adhesion randomized network set to that of the invasion randomized network set. By making this comparison across all patterns, an understanding of how similar the global null hypotheses are is obtained [24]. The comparison was accomplished by calculating the correlation coefficient between the mean number of occurrences of the 378 network patterns in the adhesion and invasion randomized network, obtaining a value of 0.974. A completely correlated null hypothesis would have given a correlation coefficient close to 1, while a completely uncorrelated null hypothesis will give a value close to 0 (due to randomization). This shows that though the networks contain different motif sets, they display similar null hypotheses. These observations demonstrate the significance of the network comparison and suggest that there is no universal set of genetic-interaction motifs that will apply uniformly to all genetic-interaction networks. Rather, analyses of each network will be necessary.

### Open source software

To facilitate the application of the analyses used in this study to other networks, we developed an open source software package entitled Network Motif Finder. Network Motif Finder was designed to identify motifs in any network type, and to include any number of edge and node types. Network Motif Finder acts as a plugin to the network analysis platform Cytoscape [36], and identifies significant multi-mode genetic interaction patterns. In addition, Network Motif Finder has the functionality of extracting motif sub-networks as shown in Figures 3 and 5. The plugin is available as open source, with a user manual, at [37].

## Conclusion

In this study we develop methods to address the challenges of analyzing complex genetic-interaction networks. Specifically, we use statistical techniques to identify biologically significant multi-mode genetic interaction network patterns, network motifs. Utilizing randomized null hypotheses of the genetic network, those patterns that occur more frequently than randomly expected can be identified. These motifs highlight biologically informative network patterns of the genetic network. Further, the union of all instances of a motif forms a motif subnetwork. These subnetworks illustrate the distribution of the motif instances within the full genetic network. This allows for the identification of all genes involved in such a motif and can highlight those genes that dominate the motif's occurrence. In this way, motif subnetworks extract the biological information that was identified by motif analysis.

We also identified network motifs that reflect the underlying biochemical network. This was done by integrating our genetic network with gene-annotation data. In this way, we describe an unbiased approach to understand how genetic interactions reflect the biological properties of the underlying system. Lastly, this analysis has been developed into an open source plugin to the network analysis software Cytoscape, allowing users to analyze their own multi-mode genetic-interaction network datasets.

## Materials and methods

### Network randomization

Statistical significance of each network pattern was calculated by comparing the number of times the pattern occurred in the observed genetic-interaction network, to a set of randomized networks. The randomized networks represent the null hypothesis. To ensure that pattern significance was due solely to the genetics of the system and not experimental design, we constrained our randomizations in the following way. First, as described in the text, the topology of the genetic interaction network defines which genetic interaction experiments were conducted, while the interaction types describe the genetic results. Thus, in all our randomizations, the topology of the network is held constant and the genetic interaction types (edge colors) are switched. Second, as described in Drees *et al*. [1] and Additional data file 22, each genetic interaction consists of the four phenotypes: ΦWT, ΦA, ΦB, ΦAB. These quantitative phenotypes are ordered into 1 of 75 possible genetic interaction inequalities, and the inequalities are grouped into 9 possible genetic interaction types. As the phenotypes of the single genetic perturbations (ΦA, ΦB) are dependent on experimental allele selection, it is necessary to avoid randomizing these single-gene phenotypes to prevent allele-selection bias in the results. Thus, in our Monte Carlo switching we strictly maintain the ordering of each edge's single-perturbation and wild-type phenotypes (ΦWT, ΦA, ΦB). In all randomizations we uniformly chose a random pair of ordered edges and exchanged their genetic interaction types only if the inequality relationship of ΦWT, ΦA, and ΦB (regardless of ΦAB) was identical for both edges. In the case of nonidentical inequality relationships, we retested after swapping the positions of ΦA and ΦB in the inequality of the second edge of the pair and exchanged only if the resulting edge inequality relationship of ΦWT, ΦA, and ΦB was identical. These methods conserve the total number of each genetic interaction edge type in all randomizations and ensure that statistical significance does not depend on initial experimental design or allele selection.

We employed a Monte Carlo method of genetic-interaction edge-type switching for the randomization algorithm. Each edge was switched in the Monte Carlo algorithm at least ten times per randomization. This level of switching has been shown to provide good mixing [24]. A sample size of 1,000 randomized networks to represent the null hypothesis was used for each analysis unless specified below. Modifications to this scheme were employed for the motifs involving annotation data and are described below. All algorithms are implemented in our open-source software package, Network Motif Finder.

In the motif analyses including GOSlim annotations, the positions of the GOSlim node annotations were held constant, and only the genetic interaction types were randomized as described above. This ensures that the underlying molecular structure of the system remains constant, while only the resulting genetic relationships are randomized. As well, we identified both 2-node and 3-node motifs. In the enumeration of 3-node network pattern instances the total number of 2-node network pattern instances was held constant. This ensures that the significance of a 3-node pattern is due to its 3-node architecture and not because it contained a significant 2-node pattern. Edge directions are conserved in this restriction. Also, the relationships between node annotations and the single gene perturbation data were maintained. Due to the extra calculations that are made during these randomizations this algorithm was much slower, particularly for the 3-node analysis. To compensate, we reduced the sample size representing the null hypothesis in the 3-node analysis from 1,000 to 500. This null hypothesis reduction was conducted for the dual invasion/adhesion network comparison as well.

Lastly, to avoid significance due to multiple testing, we corrected our significance threshold by applying the conservative Bonferroni correction. Specifically, a statistical threshold of *p *< 0.05/*n *was used, where *n *is the total number of patterns tested for significance in each analysis. For the 3n-motifs, 4n-motifs, 2nGO-motifs, and 3nGo-motifs, *n *was 489, 1,505, 575, and 23,286, respectively. To obtain a *p *value resolution greater than what is possible empirically (*p *< 1 × 10^-3 ^for a 1,000 randomized network set), we parametrically fit the null hypothesis network pattern distributions to Gaussian (or Poisson when the pattern's mean count was <3). Please see Additional data files 3, 6, 9, 20 and 21 for the network pattern distributions and parametric fits.

### Motif enumeration techniques

In all analyses except those containing 4-node patterns, a full enumeration of the network pattern instances was conducted. However, this was not computationally feasible for the 4-node patterns, and a sampling algorithm was employed [28]. There are >3 × 10^6 ^individual 4-node network pattern instances in our analyzed network; we sampled 100,000 without replacement. This sample rate is comparable to those used in other sampling studies [38].

In enumerating network patterns involving GoSlim annotations, we needed to account for genes having multiple annotations. For instance, a particular GoSlim molecular function gene may be annotated as both a transferase and a protein kinase. In enumerating a specific network pattern, we allowed genes sharing a single common annotation to be considered equal. For instance, consider the set of 1-node patterns annotated transferase, transferase/protein kinase, and protein kinase, respectively. In our scheme, we would have three patterns (transferase, transferase/protein kinase, and protein kinase), containing two, three, and two instances, respectively.

In the general motif analysis we identified motifs containing purely noninteracting edge types. It is possible that these motifs occur due to gene perturbations irrelevant to the filamentation phenotype. In our analyses using GoSlim annotations, we included such motifs when stating the percentage of significant patterns, but removed them from the Additional data files to avoid highlighting relatively uninformative patterns.

### GoSlim molecular function annotations

The GoSlim molecular function annotations were downloaded on 5 June 2006 from the Saccharomyces Genome Database [39].

## Additional data files

The following additional data are available with the online version of this paper. Additional data file 1 is a table listing the full collection of 3n-motifs. Additional data file 2 is an xml file listing the network pattern structure, significance, and number of instances of each 3node network pattern found in the genetic network. Additional data file 3 is a table listing the random distribution, parametric fit, and significance of the top 100 significant 3-node network patterns found in the genetic network. Additional data file 4 is a table listing the full collection of 4n-motifs. Additional data file 5 is an xml file listing the network pattern structure, significance, and number of instances of each 4-node network pattern found in the genetic network. Additional data file 6 is a table listing the random distribution, parametric fit, and significance of the top 100 significant 4-node network patterns found in the genetic network. Additional data file 7 is a table listing the full collection of 2nGO-motifs. Additional data file 8 is an xml file listing the network pattern structure, significance, and number of instances of each 2nGO network pattern found in the genetic network. Additional data file 9 is a table listing the random distribution, parametric fit, and significance of the top 100 significant 2nGO network patterns found in the genetic network. Additional data file 10 is a table listing the full collection of 3nGO-motifs. Additional data files 11 and 12 are xml files listing the network pattern structure, significance, and number of instances of each 3nGO network pattern found in the genetic network. Additional data file 13 is an xml document describing the genetic perturbations used to construct the genetic interaction network. Additional data file 14 is a Cytoscape attribute file containing the genetic inequality relationships for all edges of the genetic interaction network Additional data file 15 is a Cytoscape network file of the genetic interaction network. Additional data file 16 is a Cytoscape network file containing a subset of the full genetic interaction network. Additional file 17 is a Cytoscape attribute file containing GOSlim molecular function attributes. Additional file 18 is the NetworkMotifFinder Cytoscape plugin file. Additional file 19 is a software tutorial for the NetworkMotifFinder plugin. Additional data files 20 and 21 are table listings of the random distribution, parametric fit, and significance of the top 200 significant 3nGO network patterns found in the genetic network. Additional data files 22, 23, 24, 25 contain the supplemental figures. Additional data file 22 contains supplemental Figure 1, which defines the genetic interactions used in this study. Additional data file 23 contains supplemental Figure 2a,b, which gives further examples of 3n-motif subnetworks. Additional data file 24 contains supplemental Figure 2c,d, which give further examples of 3n-motif subnetworks. Additional data file 25 contains supplemental Figure 2e, which gives further examples of 4n-motifs.

## Abbreviations

ΦA = phenotype of genetic perturbation A; ΦAB = phenotype of the double A and B genetic perturbation; ΦB = phenotype of genetic perturbation B; ΦWT = wild type phenotype; 2nGO-motifs = significant 2-node network patterns identified from the genetic network integrated with GOSLIM molecular function annotations; 3nGO-motifs = significant 3-node network patterns identified from the genetic network integrated with GOSLIM molecular function annotations; 3n-motifs = significant 3-node network patterns; 4n-motifs = significant 4-node network patterns; fMAPK = filamentation/invasion MAP-kinase pathway.

## Authors' contributions

RJT collaborated in study design, performed all analyses, developed the software, and drafted the manuscript. AFS collaborated in statistical analyses. TG conceived of the study, collaborated in its design, and revised the manuscript.

## Supplementary Material

Additional data file 1 Full collection of 3n-motifs. Click here for file

Additional data file 2 Network pattern structure, significance, and number of instances of each 3node network pattern found in the genetic network. Click here for file

Additional data file 3 Random distribution, parametric fit, and significance of the top 100 significant 3-node network patterns found in the genetic network. Click here for file

Additional data file 4 Full collection of 4n-motifs. Click here for file

Additional data file 5 Network pattern structure, significance, and number of instances of each 4-node network pattern found in the genetic network. Click here for file

Additional data file 6 Random distribution, parametric fit, and significance of the top 100 significant 4-node network patterns found in the genetic network. Click here for file

Additional data file 7 Full collection of 2nGO-motifs. Click here for file

Additional data file 8 Network pattern structure, significance, and number of instances of each 2nGO network pattern found in the genetic network. Click here for file

Additional data file 9 Random distribution, parametric fit, and significance of the top 100 significant 2nGO network patterns found in the genetic network. Click here for file

Additional data file 10 Full collection of 3nGO-motifs. Click here for file

Additional data file 11 Network pattern structure, significance, and number of instances of each 3nGO network pattern found in the genetic network. Click here for file

Additional data file 12 Network pattern structure, significance, and number of instances of each 3nGO network pattern found in the genetic network. Click here for file

Additional data file 13 Description of the genetic perturbations used to construct the genetic interaction network. Click here for file

Additional data file 14 Cytoscape attribute file containing the genetic inequality relationships for all edges of the genetic interaction network. Click here for file

Additional data file 15 Cytoscape network file of the genetic interaction network Click here for file

Additional data file 16 Cytoscape network file containing a subset of the full genetic interaction network. Click here for file

Additional data file 17 Cytoscape attribute file containing GOSlim molecular function attributes. Click here for file

Additional data file 18 NetworkMotifFinder Cytoscape plugin file. Click here for file

Additional data file 19 Software tutorial for the NetworkMotifFinder plugin. Click here for file

Additional data file 20 Random distribution, parametric fit, and significance of the top 200 significant 3nGO network patterns found in the genetic network. Click here for file

Additional data file 21 Random distribution, parametric fit, and significance of the top 200 significant 3nGO network patterns found in the genetic network. Click here for file

Additional data file 22 Supplemental Figure 1: definition of the genetic interactions used in this study. Click here for file

Additional data file 23 Supplemental Figure 2a,b: further examples of 3n-motif subnetworks. Click here for file

Additional data file 24 Supplemental Figure 2c,d: further examples of 3n-motif subnetworks. Click here for file

Additional data file 25 Supplemental Figure 2e: further examples of 4n-motif subnetworks. Click here for file
