# Supplementary material for: Network motif analysis of a multi-mode genetic-interaction network
Source: Genome Biol. 2007 Aug 2;8(8):R160. doi: 10.1186/gb-2007-8-8-r160 (PMC2374991; doi:10.1186/gb-2007-8-8-r160)
Supplement: Additional data file 1 — Full collection of 3n-motifs. [file gb-2007-8-8-r160-S1.pdf]

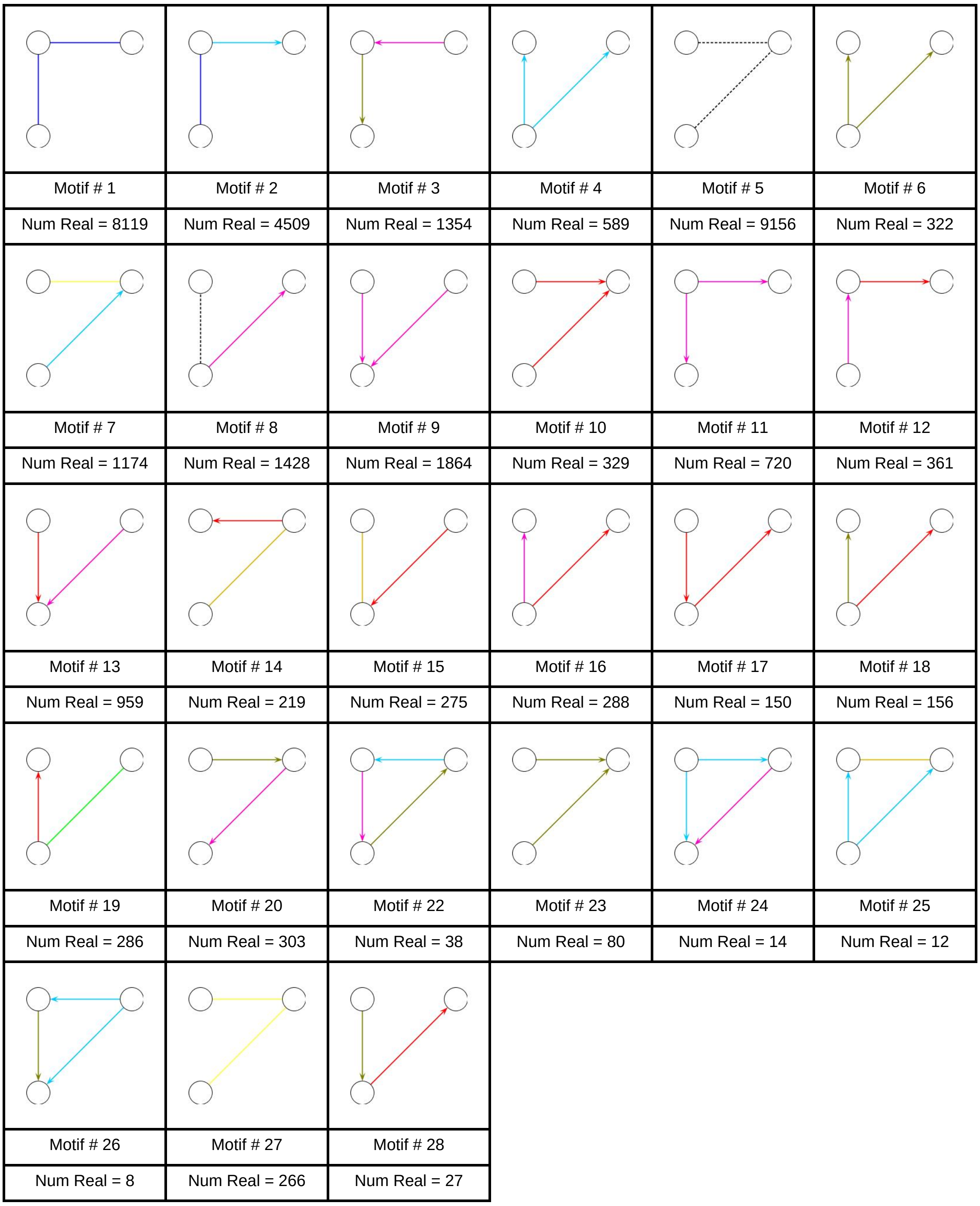
Motif # 1
Num Real = 8119
Motif # 2
Num Real = 4509
Motif # 3
Num Real = 1354
Motif # 4
Num Real = 589
Motif # 5
Num Real = 9156
Motif # 6
Num Real = 322
Motif # 7
Num Real = 1174
Motif # 8
Num Real = 1428
Motif # 9
Num Real = 1864
Motif # 10
Num Real = 329
Motif # 11
Num Real = 720
Motif # 12
Num Real = 361
Motif # 13
Num Real = 959
Motif # 14
Num Real = 219
Motif # 15
Num Real = 275
Motif # 16
Num Real = 288
Motif # 17
Num Real = 150
Motif # 18
Num Real = 156
Motif # 19
Num Real = 286
Motif # 20
Num Real = 303
Motif # 22
Num Real = 38
Motif # 23
Num Real = 80
Motif # 24
Num Real = 14
Motif # 25
Num Real = 12
Motif # 26
Num Real = 8
Motif # 27
Num Real = 266
Motif # 28
Num Real = 27
