# Supplementary material for: Network motif analysis of a multi-mode genetic-interaction network
Source: Genome Biol. 2007 Aug 2;8(8):R160. doi: 10.1186/gb-2007-8-8-r160 (PMC2374991; doi:10.1186/gb-2007-8-8-r160)
Supplement: Additional data file 3 — Random distribution, parametric fit, and significance of the top 100 significant 3-node network patterns found in the genetic network. [file gb-2007-8-8-r160-S3.pdf]

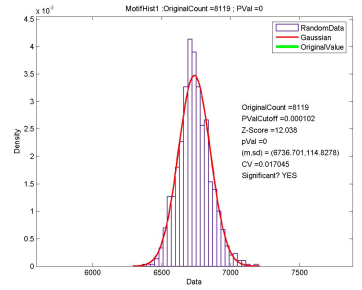

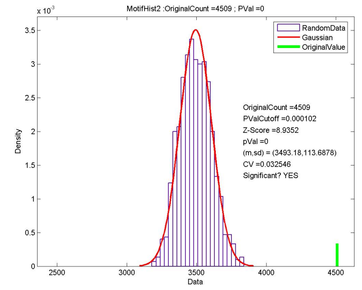

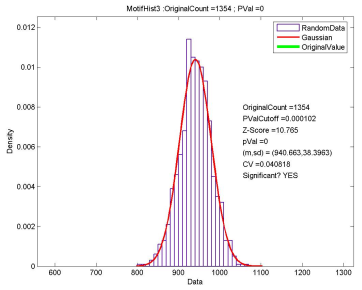

Motif # 1	Motif # 2	Motif # 3
zScore = 12.038	zScore = 8.9352	zScore = 10.765

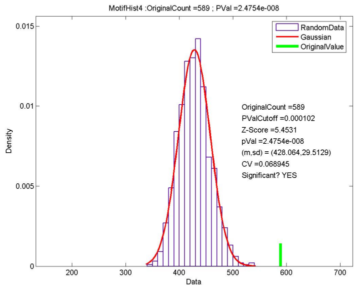

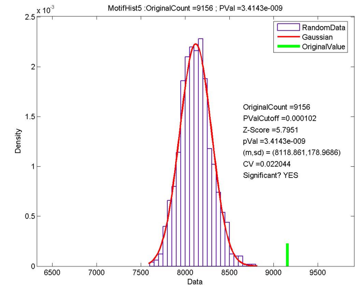

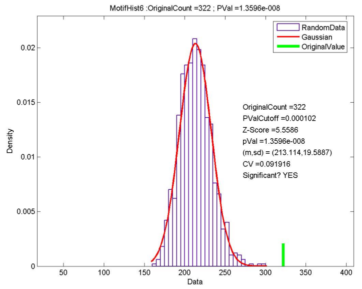

Motif # 4	Motif # 5	Motif # 6
zScore = 5.4531	zScore = 5.7951	zScore = 5.5586

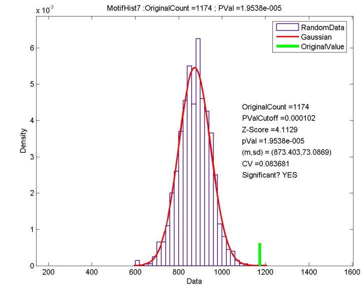

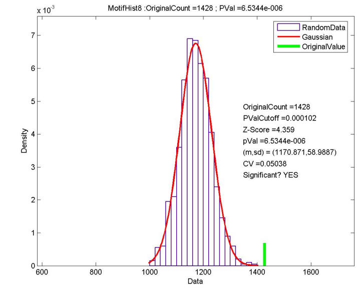

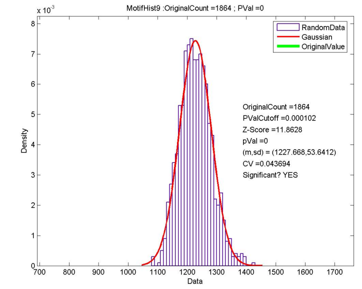

Motif # 7	Motif # 8	Motif # 9
zScore = 4.1129	zScore = 4.359	zScore = 11.8628

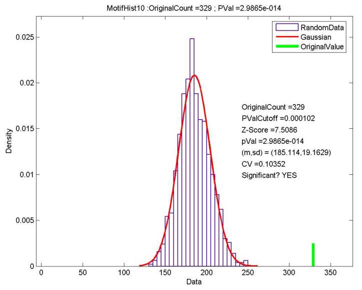

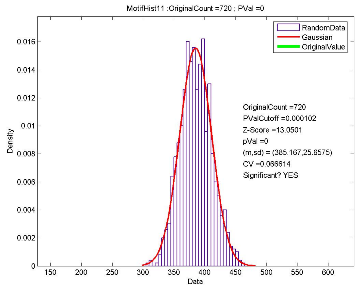

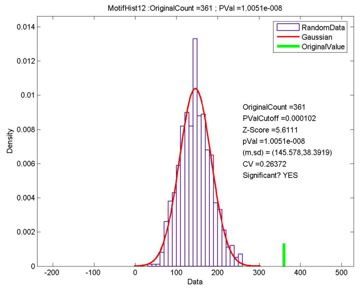

Motif # 10	Motif # 11	Motif # 12
zScore = 7.5086	zScore = 13.0501	zScore = 5.6111

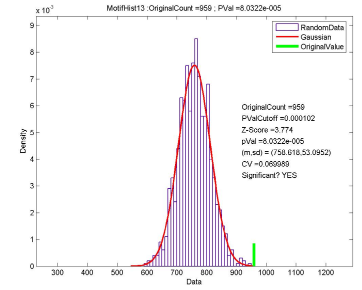

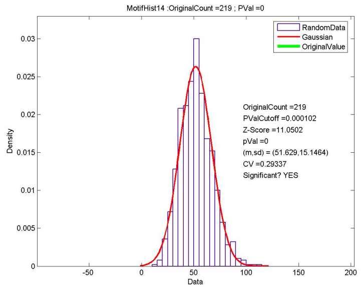

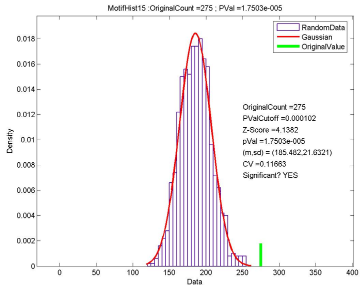

Motif # 13	Motif # 14	Motif # 15

<table>
<tr><td>zScore = 3.774</td><td>zScore = 11.0502</td><td>zScore = 4.1382</td></tr>
<tr><td>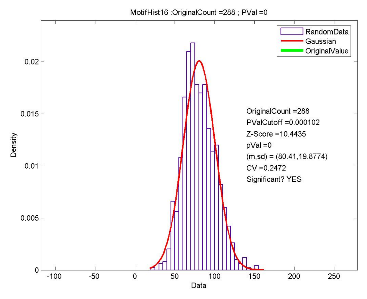
</td><td>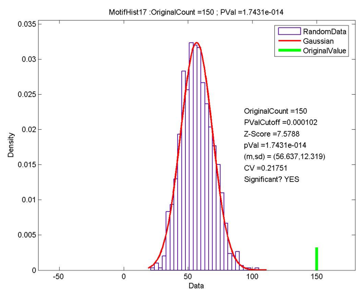
</td><td>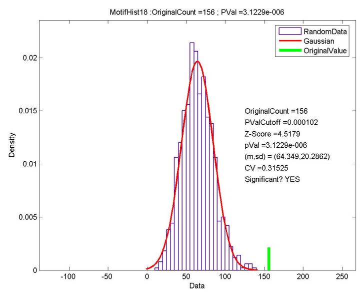
</td></tr>
<tr><td>Motif # 16</td><td>Motif # 17</td><td>Motif # 18</td></tr>
<tr><td>zScore = 10.4435</td><td>zScore = 7.5788</td><td>zScore = 4.5179</td></tr>
<tr><td>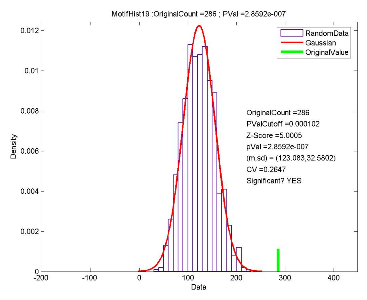
</td><td>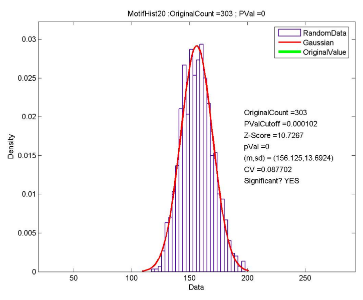
</td><td>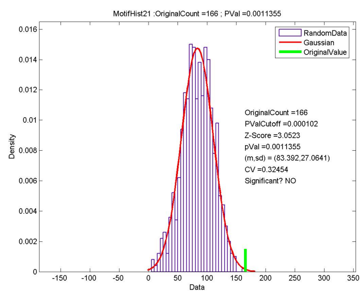
</td></tr>
<tr><td>Motif # 19</td><td>Motif # 20</td><td>Motif # 21</td></tr>
<tr><td>zScore = 5.0005</td><td>zScore = 10.7267</td><td>zScore = 3.0523</td></tr>
<tr><td>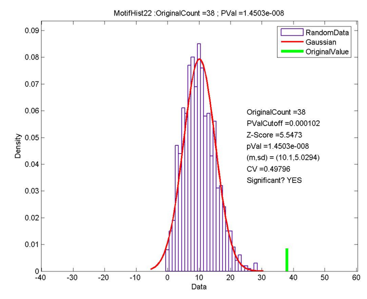
</td><td>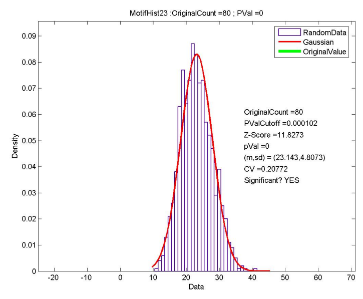
</td><td>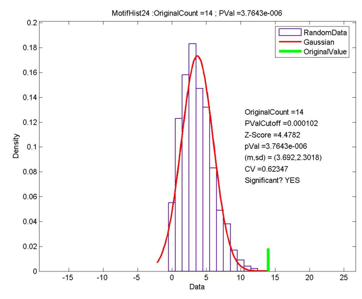
</td></tr>
<tr><td>Motif # 22</td><td>Motif # 23</td><td>Motif # 24</td></tr>
<tr><td>zScore = 5.5473</td><td>zScore = 11.8273</td><td>zScore = 4.4782</td></tr>
<tr><td>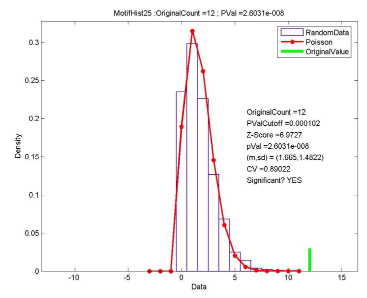
</td><td>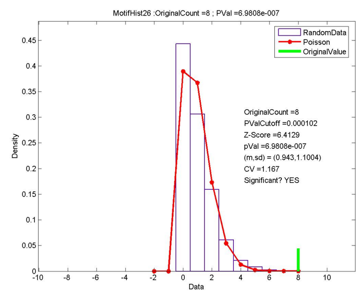
</td><td>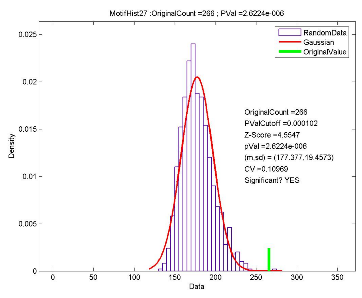
</td></tr>
<tr><td>Motif # 25</td><td>Motif # 26</td><td>Motif # 27</td></tr>
<tr><td>zScore = 6.9727</td><td>zScore = 6.4129</td><td>zScore = 4.5547</td></tr>
<tr><td>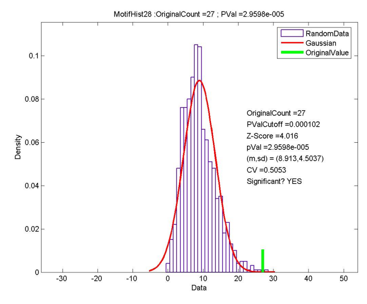
</td><td>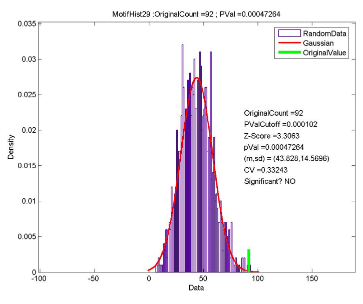
</td><td>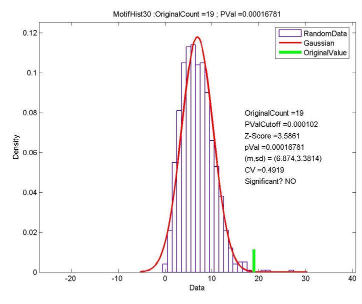
</td></tr>
</table>

Motif # 28	Motif # 29	Motif # 30
zScore = 4.016	zScore = 3.3063	zScore = 3.5861

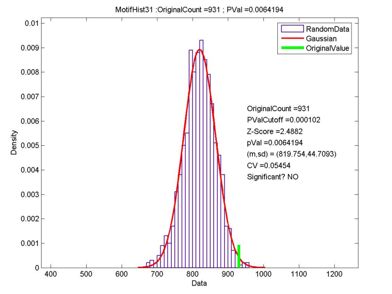

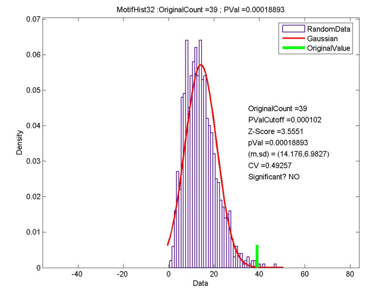

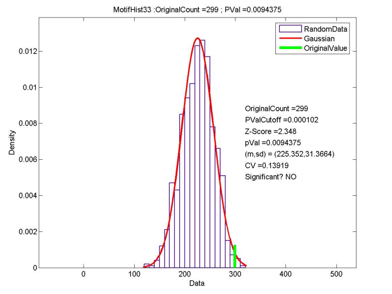

Motif # 31	Motif # 32	Motif # 33
zScore = 2.4882	zScore = 3.5551	zScore = 2.348

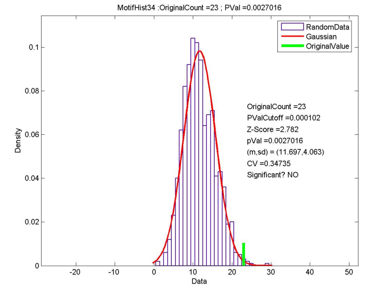

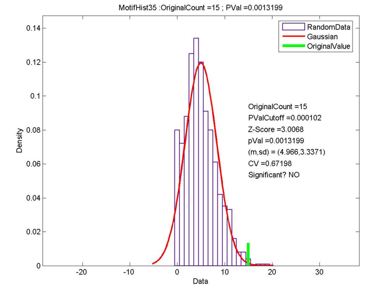

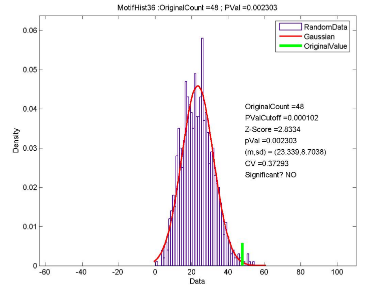

Motif # 34	Motif # 35	Motif # 36
zScore = 2.782	zScore = 3.0068	zScore = 2.8334

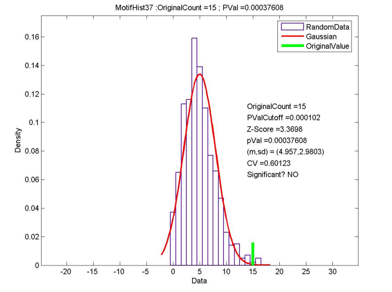

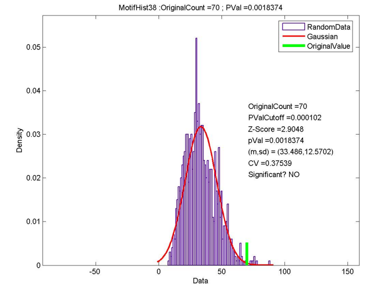

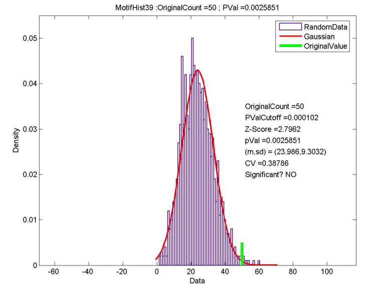

Motif # 37	Motif # 38	Motif # 39
zScore = 3.3698	zScore = 2.9048	zScore = 2.7962

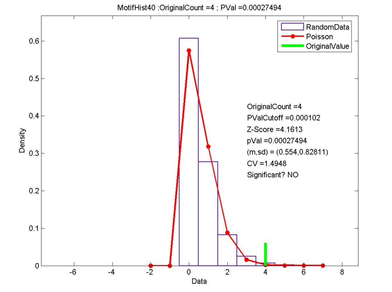

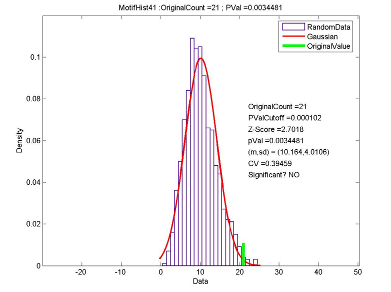

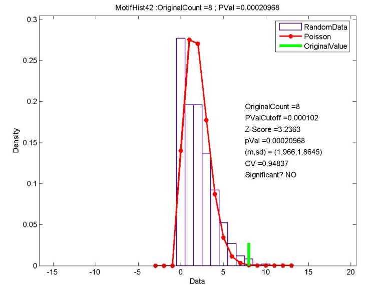

Motif # 40	Motif # 41	Motif # 42
zScore = 4.1613	zScore = 2.7018	zScore = 3.2363

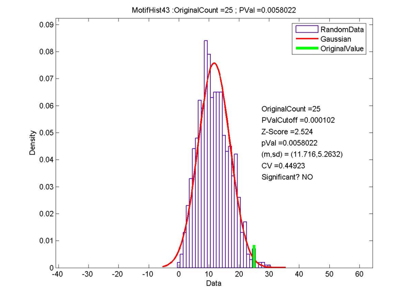

Motif # 43

zScore = 2.524

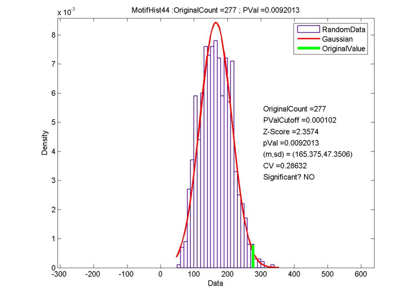

Motif # 44

zScore = 2.3574

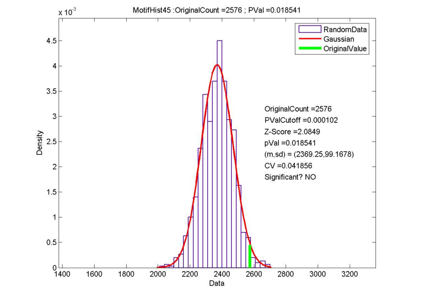

Motif # 45

zScore = 2.0849

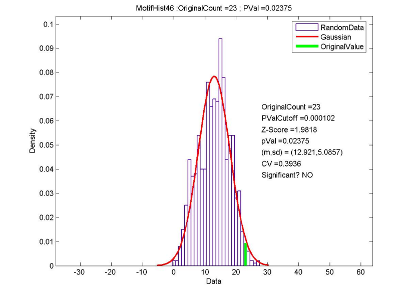

Motif # 46

zScore = 1.9818

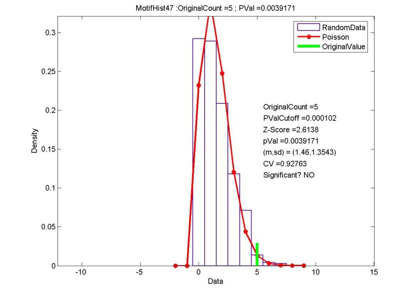

Motif # 47

zScore = 2.6138

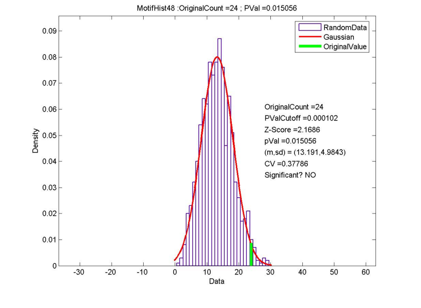

Motif # 48

zScore = 2.1686

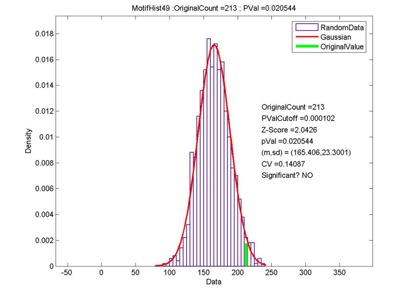

Motif # 49

zScore = 2.0426

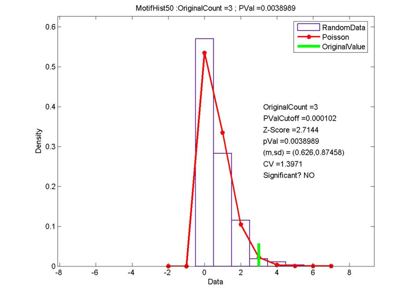

Motif # 50

zScore = 2.7144

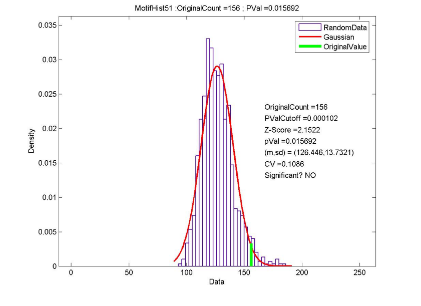

Motif # 51

zScore = 2.1522

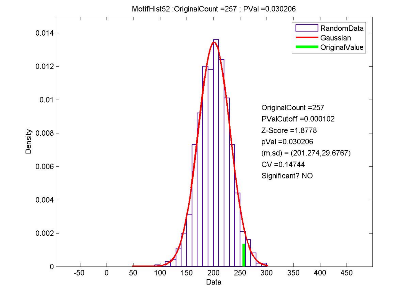

Motif # 52

zScore = 1.8778

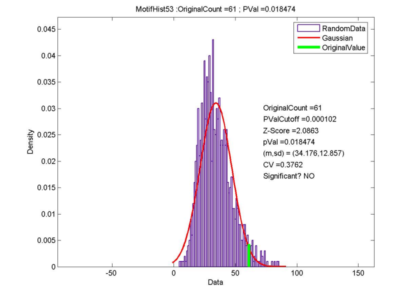

Motif # 53

zScore = 2.0863

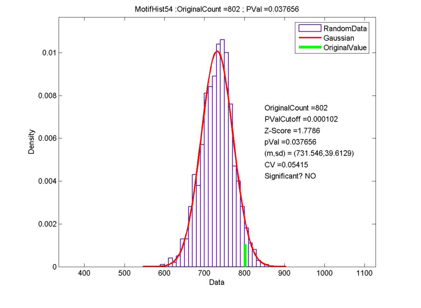

Motif # 54

zScore = 1.7786

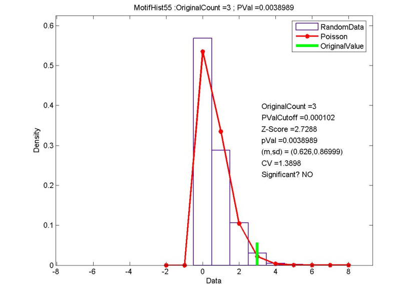

Motif # 55

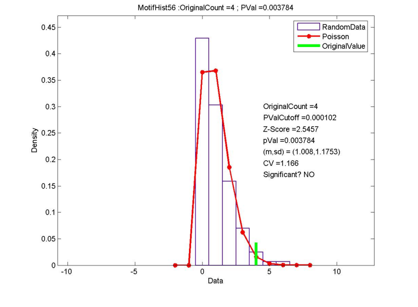

Motif # 56

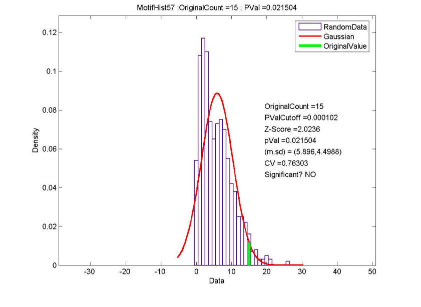

Motif # 57

zScore = 2.7288

zScore = 2.5457

zScore = 2.0236

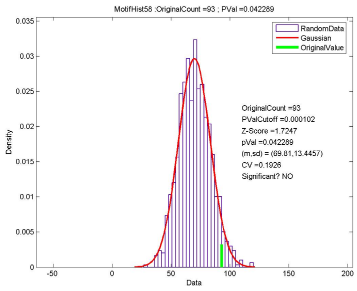

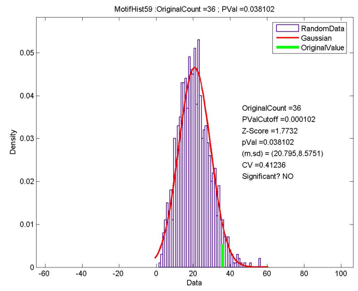

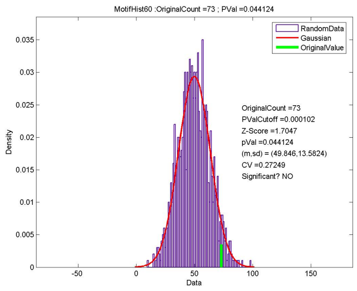

Motif # 58

Motif # 59

Motif # 60

zScore = 1.7247

zScore = 1.7732

zScore = 1.7047

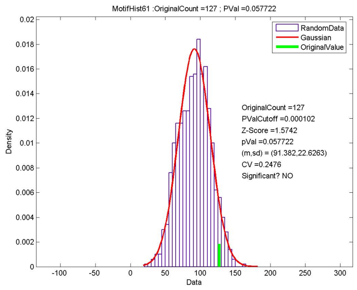

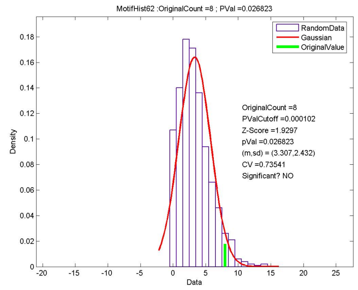

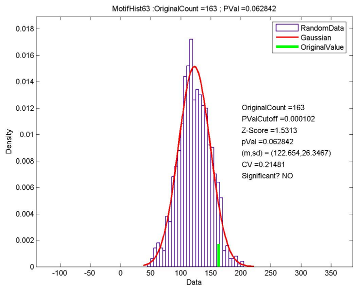

Motif # 61

Motif # 62

Motif # 63

zScore = 1.5742

zScore = 1.9297

zScore = 1.5313

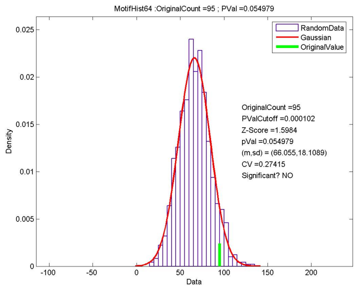

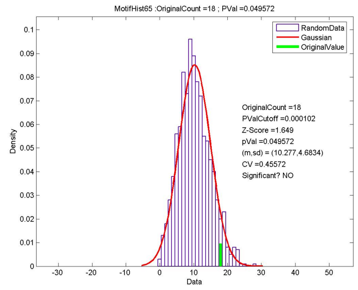

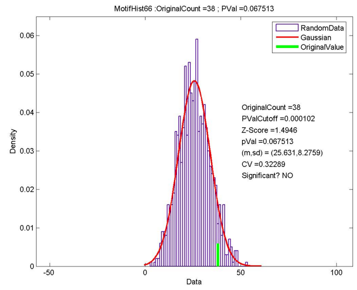

Motif # 64

Motif # 65

Motif # 66

zScore = 1.5984

zScore = 1.649

zScore = 1.4946

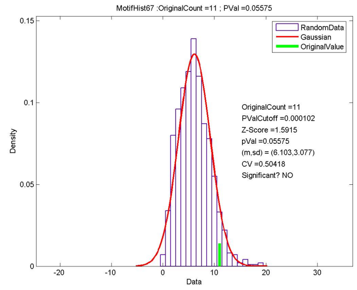

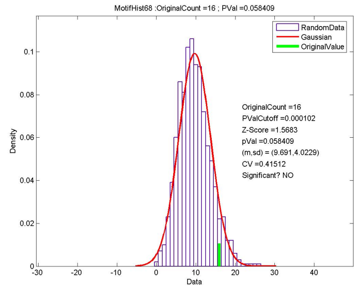

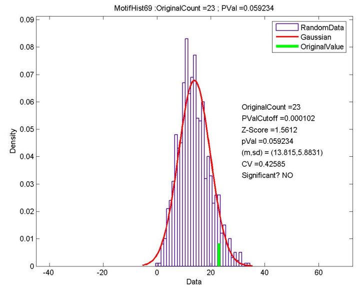

Motif # 67

Motif # 68

Motif # 69

zScore = 1.5915

zScore = 1.5683

zScore = 1.5612

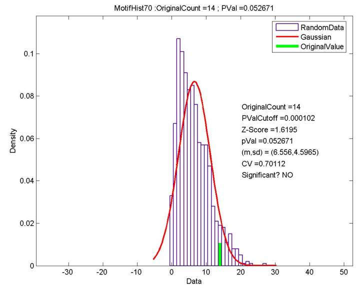

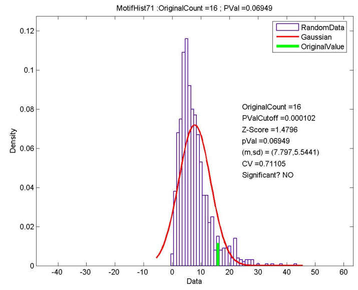

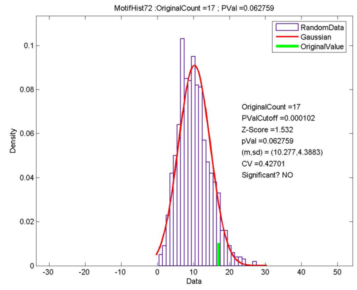

Motif # 70	Motif # 71	Motif # 72
zScore = 1.6195	zScore = 1.4796	zScore = 1.532

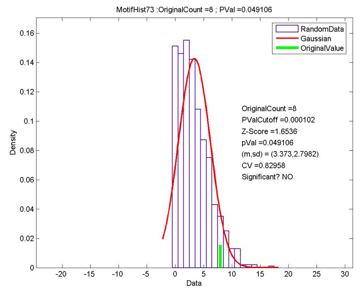

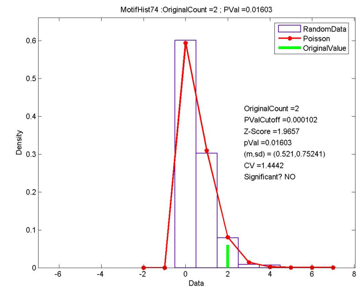

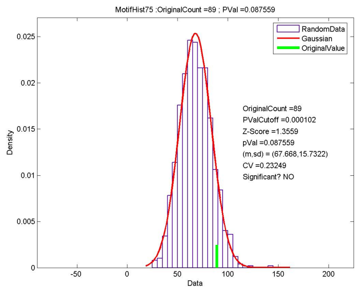

Motif # 73	Motif # 74	Motif # 75
zScore = 1.6536	zScore = 1.9657	zScore = 1.3559

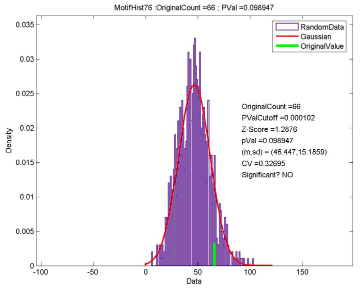

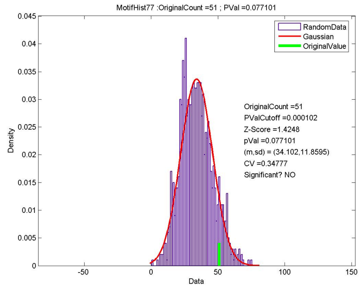

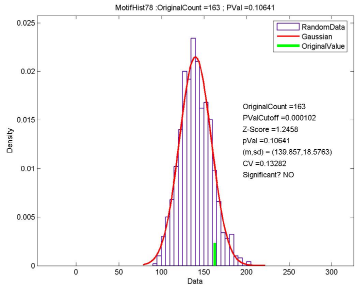

Motif # 76	Motif # 77	Motif # 78
zScore = 1.2876	zScore = 1.4248	zScore = 1.2458

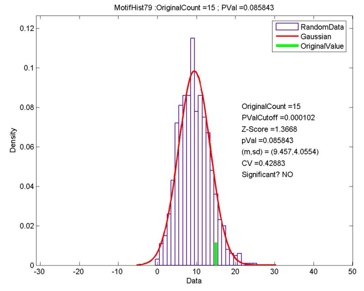

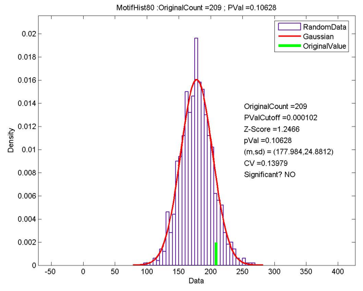

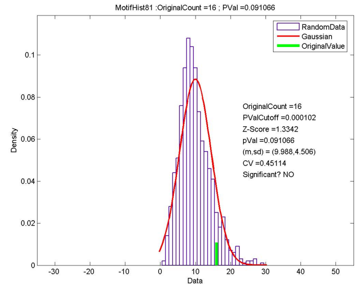

Motif # 79	Motif # 80	Motif # 81
zScore = 1.3668	zScore = 1.2466	zScore = 1.3342

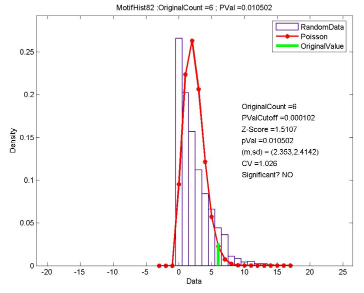

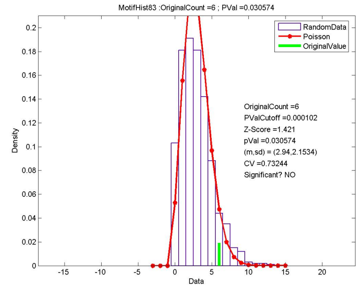

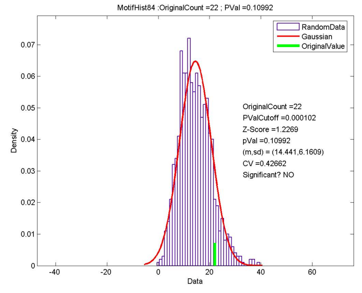

Motif # 82	Motif # 83	Motif # 84
zScore = 1.5107	zScore = 1.421	zScore = 1.2269

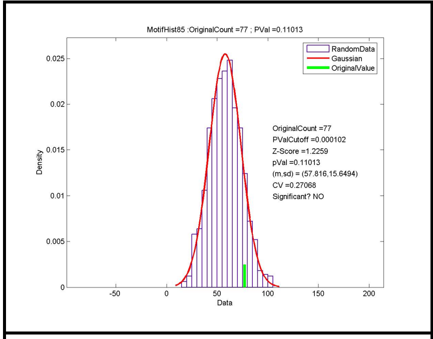

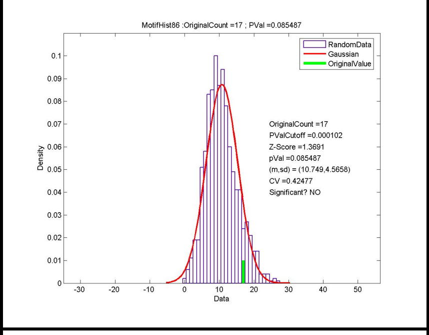

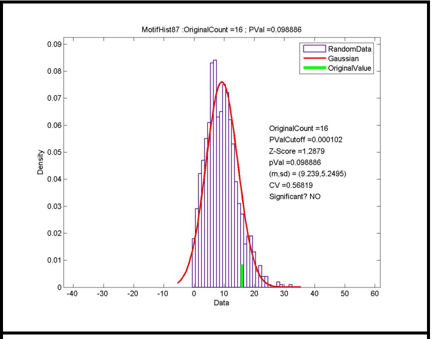

Motif # 85	Motif # 86	Motif # 87
zScore = 1.2259	zScore = 1.3691	zScore = 1.2879

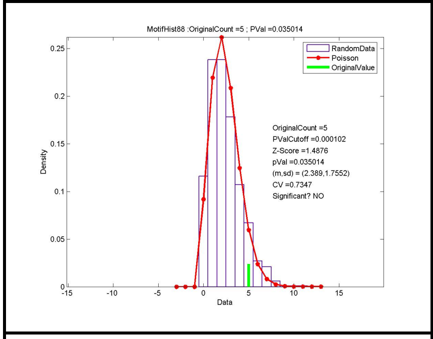

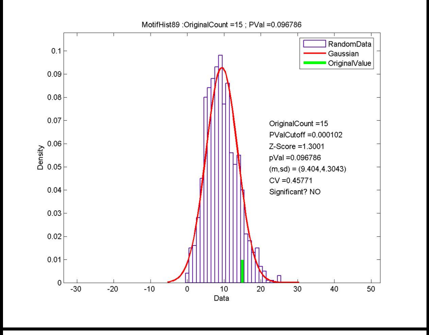

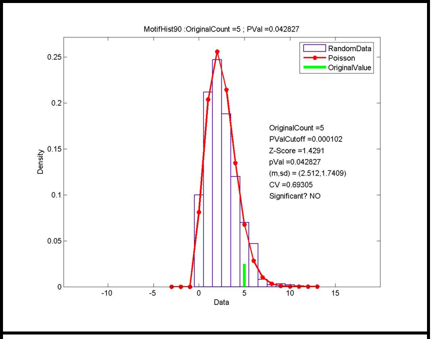

Motif # 88	Motif # 89	Motif # 90
zScore = 1.4876	zScore = 1.3001	zScore = 1.4291

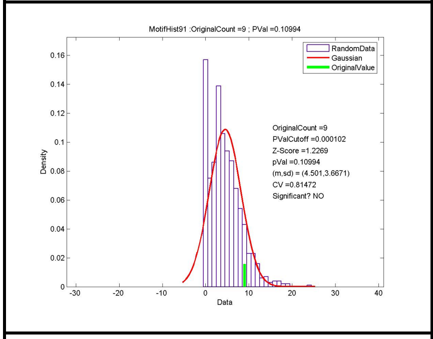

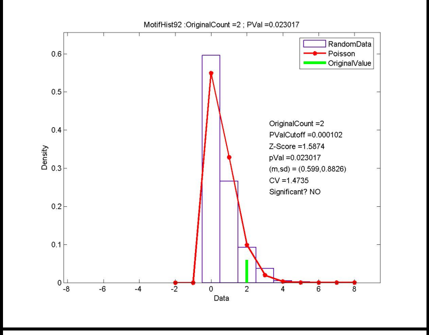

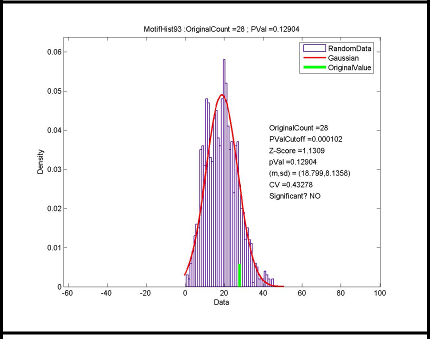

Motif # 91	Motif # 92	Motif # 93
zScore = 1.2269	zScore = 1.5874	zScore = 1.1309

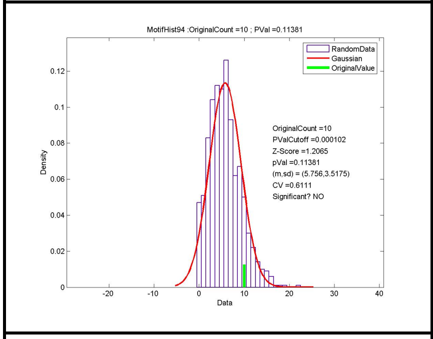

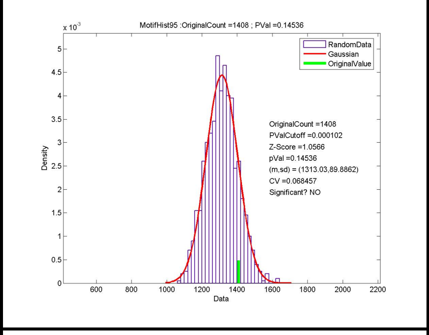

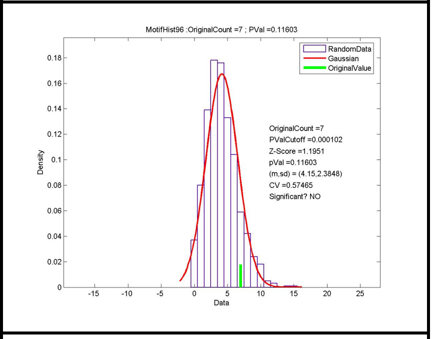

Motif # 94	Motif # 95	Motif # 96
zScore = 1.2065	zScore = 1.0566	zScore = 1.1951

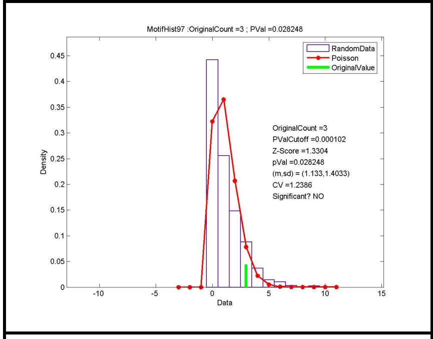

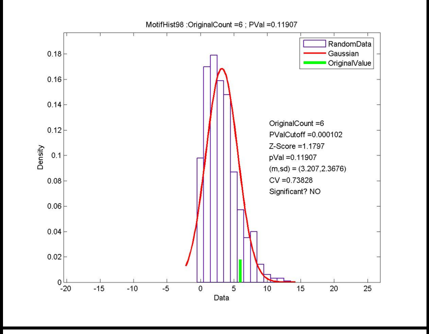

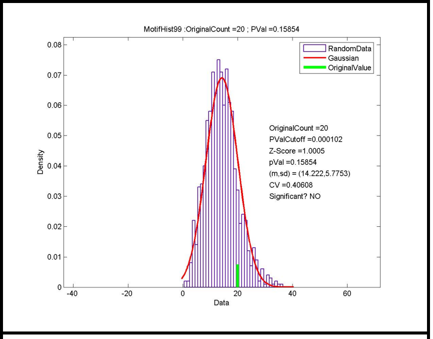

Motif # 97	Motif # 98	Motif # 99

zScore = 1.3304	zScore = 1.1797	zScore = 1.0005

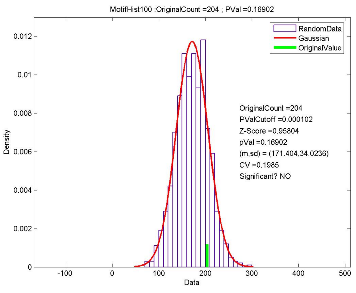

Motif # 100
zScore = 0.95804
