# Supplementary material for: Network motif analysis of a multi-mode genetic-interaction network
Source: Genome Biol. 2007 Aug 2;8(8):R160. doi: 10.1186/gb-2007-8-8-r160 (PMC2374991; doi:10.1186/gb-2007-8-8-r160)
Supplement: Additional data file 4 — Full collection of 4n-motifs. [file gb-2007-8-8-r160-S4.pdf]

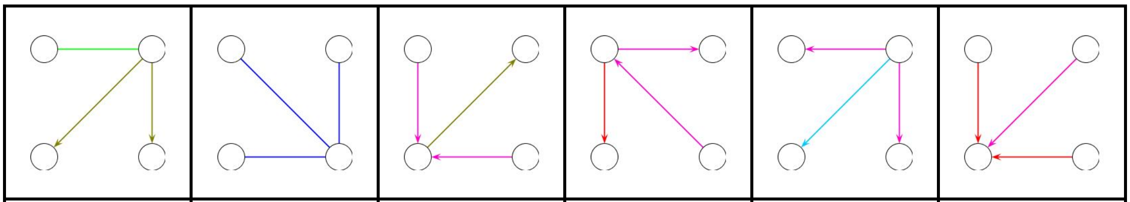

Motif # 1	Motif # 2	Motif # 3	Motif # 4	Motif # 5	Motif # 6
Num Real = 187	Num Real = 4548	Num Real = 547	Num Real = 103	Num Real = 114	Num Real = 133

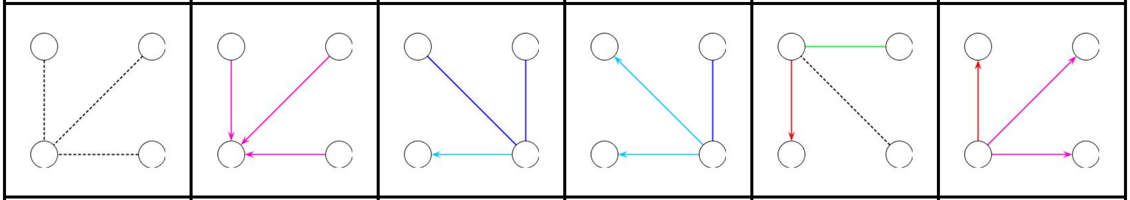

Motif # 7	Motif # 8	Motif # 9	Motif # 10	Motif # 11	Motif # 12
Num Real = 4969	Num Real = 558	Num Real = 3694	Num Real = 963	Num Real = 167	Num Real = 44

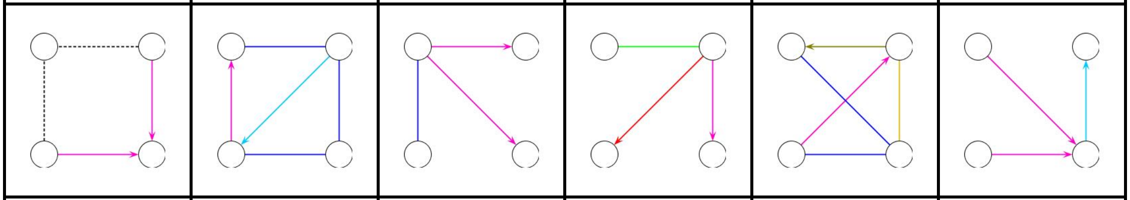

Motif # 13	Motif # 14	Motif # 15	Motif # 16	Motif # 17	Motif # 18
Num Real = 52	Num Real = 17	Num Real = 371	Num Real = 95	Num Real = 20	Num Real = 425

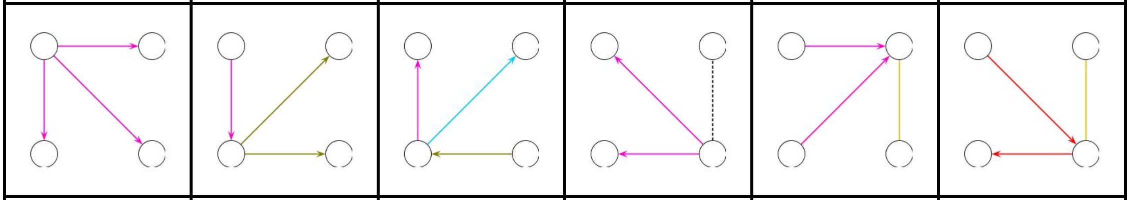

Motif # 19	Motif # 20	Motif # 21	Motif # 22	Motif # 23	Motif # 24
Num Real = 110	Num Real = 227	Num Real = 66	Num Real = 240	Num Real = 296	Num Real = 35

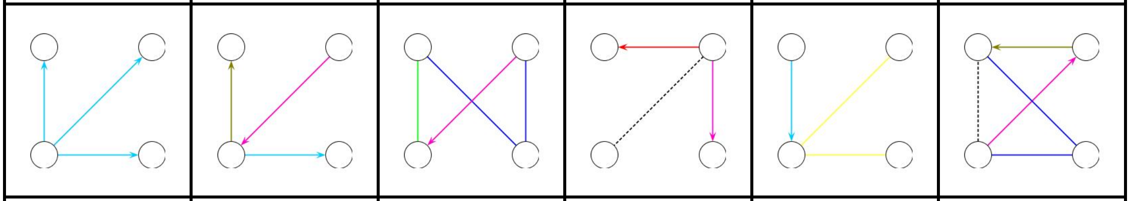

Motif # 25	Motif # 26	Motif # 27	Motif # 28	Motif # 29	Motif # 30
Num Real = 86	Num Real = 189	Num Real = 97	Num Real = 135	Num Real = 172	Num Real = 7

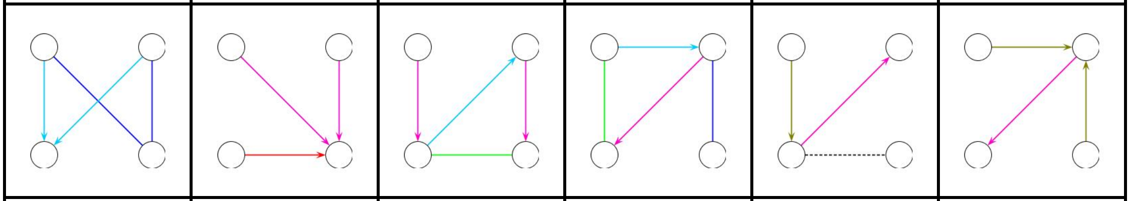

Motif # 31	Motif # 32	Motif # 33	Motif # 34	Motif # 35	Motif # 36
Num Real = 109	Num Real = 404	Num Real = 8	Num Real = 7	Num Real = 80	Num Real = 23

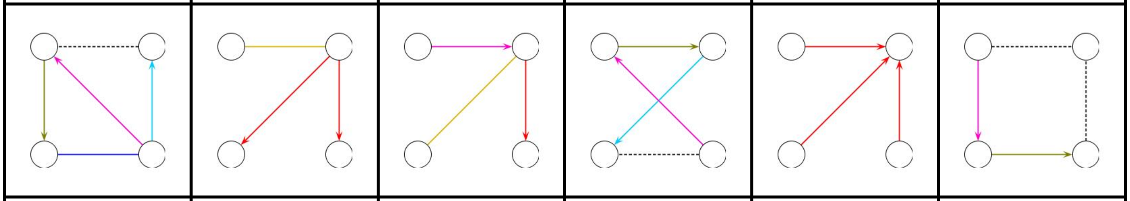

Motif # 37	Motif # 39	Motif # 40	Motif # 41	Motif # 42	Motif # 43
Num Real = 20	Num Real = 22	Num Real = 78	Num Real = 25	Num Real = 52	Num Real = 33

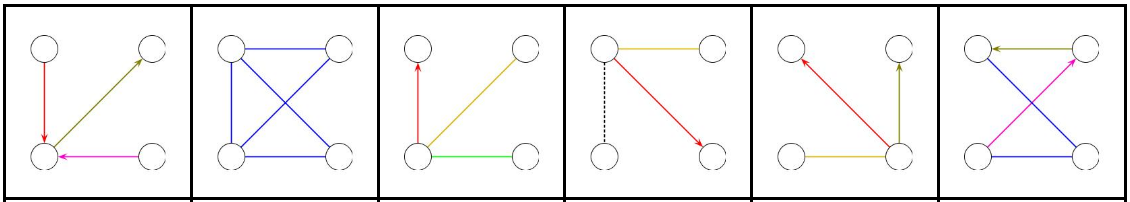

Motif # 44	Motif # 45	Motif # 46	Motif # 47	Motif # 48	Motif # 49
Num Real = 177	Num Real = 120	Num Real = 55	Num Real = 109	Num Real = 39	Num Real = 78

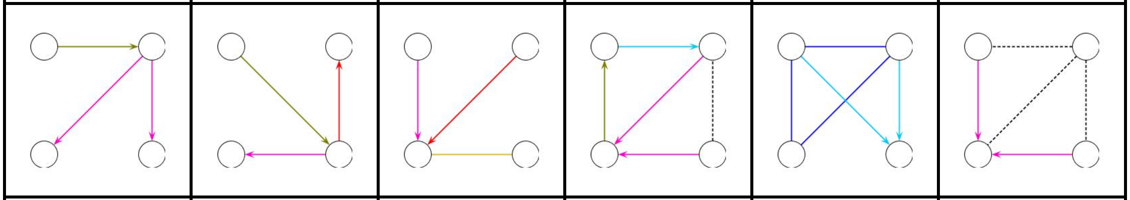

Motif # 51	Motif # 52	Motif # 53	Motif # 54	Motif # 55	Motif # 56
Num Real = 105	Num Real = 24	Num Real = 146	Num Real = 11	Num Real = 46	Num Real = 23

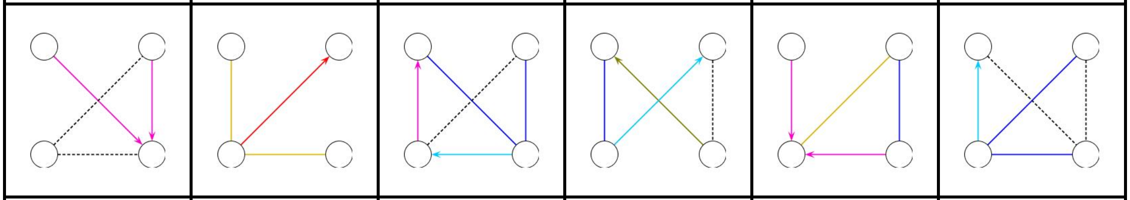

Motif # 57	Motif # 58	Motif # 59	Motif # 60	Motif # 61	Motif # 62
Num Real = 33	Num Real = 40	Num Real = 17	Num Real = 45	Num Real = 20	Num Real = 55

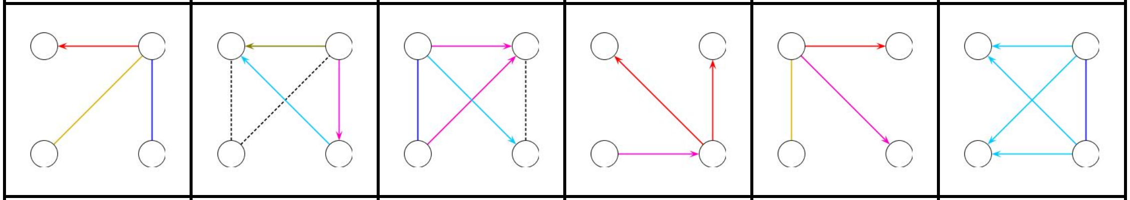

Motif # 63	Motif # 64	Motif # 65	Motif # 66	Motif # 67	Motif # 68
Num Real = 40	Num Real = 12	Num Real = 25	Num Real = 33	Num Real = 52	Num Real = 9

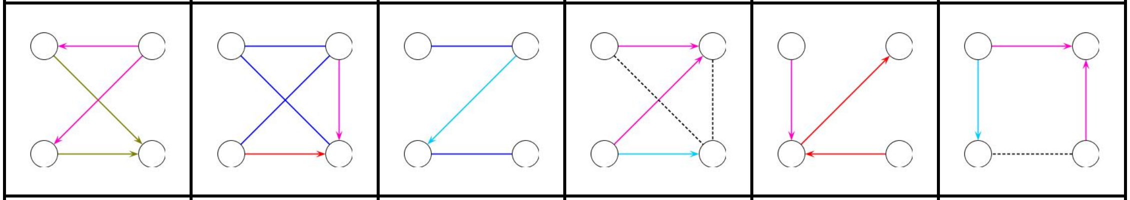

Motif # 70	Motif # 71	Motif # 72	Motif # 73	Motif # 74	Motif # 75
Num Real = 13	Num Real = 11	Num Real = 35	Num Real = 16	Num Real = 53	Num Real = 58

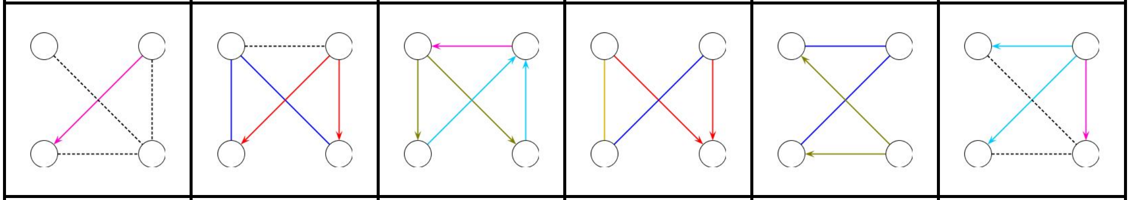

Motif # 77	Motif # 78	Motif # 79	Motif # 80	Motif # 81	Motif # 82
Num Real = 38	Num Real = 27	Num Real = 8	Num Real = 5	Num Real = 23	Num Real = 15

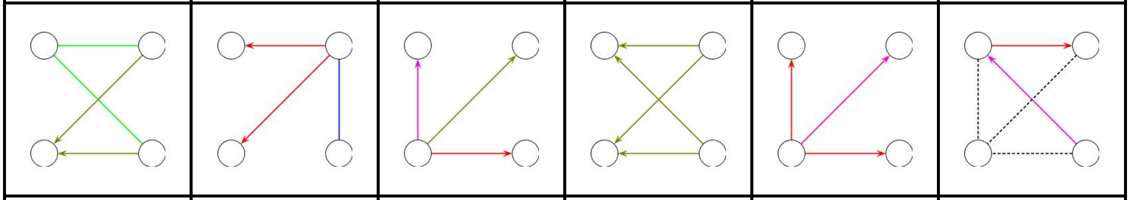

Motif # 83	Motif # 84	Motif # 85	Motif # 86	Motif # 87	Motif # 88
Num Real = 12	Num Real = 18	Num Real = 44	Num Real = 5	Num Real = 29	Num Real = 10

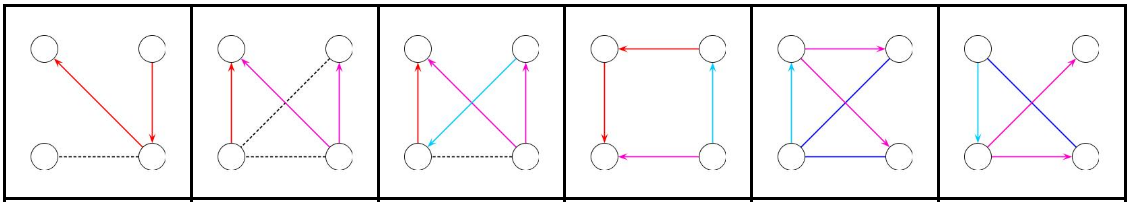

Motif # 89	Motif # 90	Motif # 91	Motif # 92	Motif # 93	Motif # 94
Num Real = 50	Num Real = 8	Num Real = 6	Num Real = 10	Num Real = 8	Num Real = 5

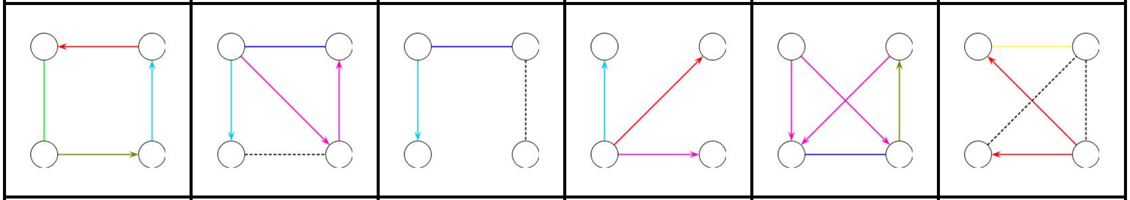

Motif # 95	Motif # 96	Motif # 97	Motif # 98	Motif # 99	Motif # 100
Num Real = 23	Num Real = 18	Num Real = 30	Num Real = 39	Num Real = 8	Num Real = 11

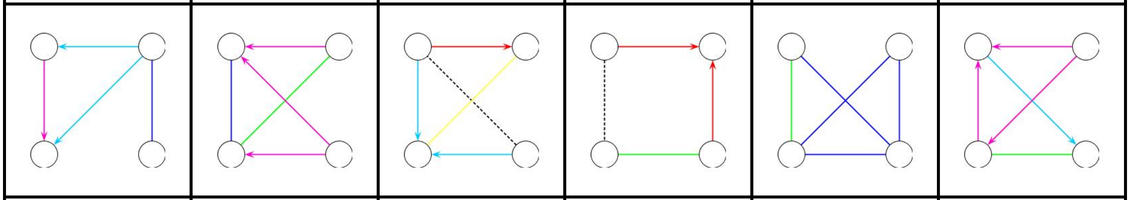

Motif # 101	Motif # 102	Motif # 103	Motif # 104	Motif # 105	Motif # 106
Num Real = 6	Num Real = 5	Num Real = 10	Num Real = 9	Num Real = 13	Num Real = 5

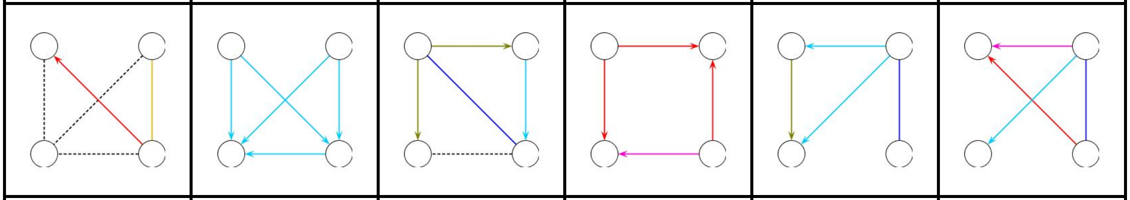

Motif # 107	Motif # 108	Motif # 109	Motif # 110	Motif # 111	Motif # 112
Num Real = 8	Num Real = 14	Num Real = 6	Num Real = 6	Num Real = 5	Num Real = 5

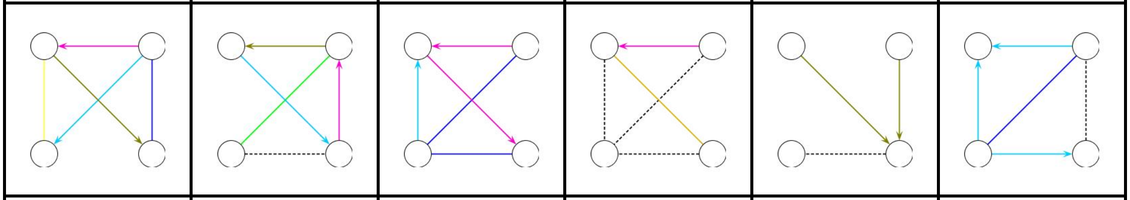

Motif # 113	Motif # 114	Motif # 115	Motif # 116	Motif # 117	Motif # 119
Num Real = 7	Num Real = 10	Num Real = 15	Num Real = 16	Num Real = 10	Num Real = 10

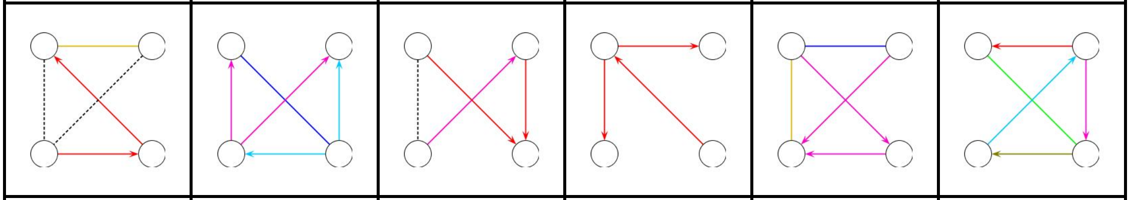

Motif # 120	Motif # 122	Motif # 124	Motif # 125	Motif # 126	Motif # 127
Num Real = 5	Num Real = 9	Num Real = 9	Num Real = 14	Num Real = 10	Num Real = 13

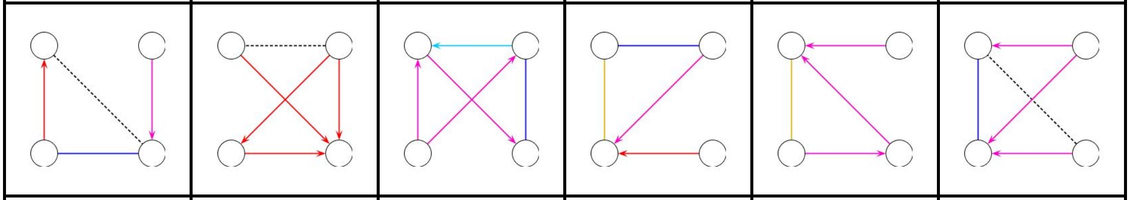

Motif # 128	Motif # 129	Motif # 130	Motif # 131	Motif # 132	Motif # 133
Num Real = 7	Num Real = 5	Num Real = 6	Num Real = 8	Num Real = 6	Num Real = 7

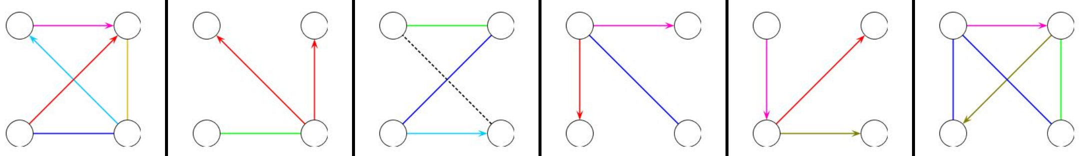

Motif # 135	Motif # 136	Motif # 137	Motif # 138	Motif # 139	Motif # 140
Num Real = 6	Num Real = 20	Num Real = 71	Num Real = 71	Num Real = 78	Num Real = 22

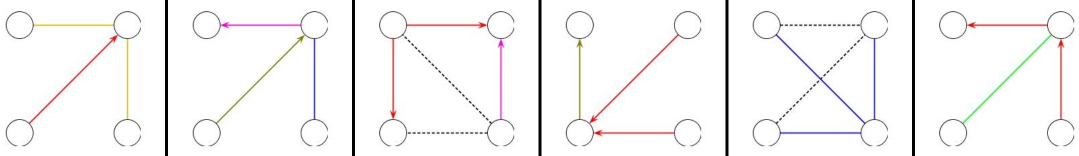

Motif # 142	Motif # 144	Motif # 145	Motif # 146	Motif # 147	Motif # 149
Num Real = 30	Num Real = 169	Num Real = 7	Num Real = 28	Num Real = 68	Num Real = 29

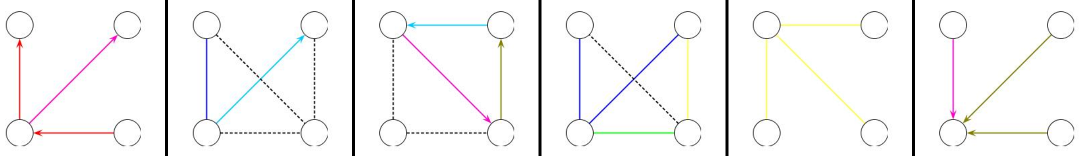

Motif # 150	Motif # 151	Motif # 153	Motif # 155	Motif # 156	Motif # 158
Num Real = 28	Num Real = 45	Num Real = 18	Num Real = 6	Num Real = 33	Num Real = 14

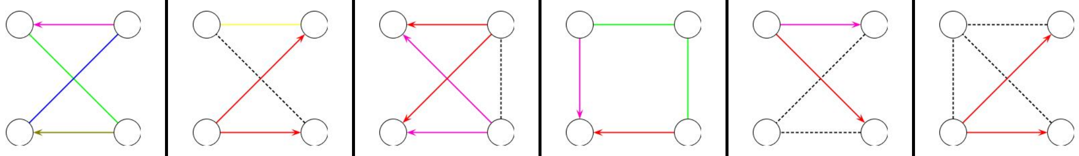

Motif # 159	Motif # 161	Motif # 162	Motif # 165	Motif # 166	Motif # 167
Num Real = 18	Num Real = 12	Num Real = 5	Num Real = 6	Num Real = 7	Num Real = 30

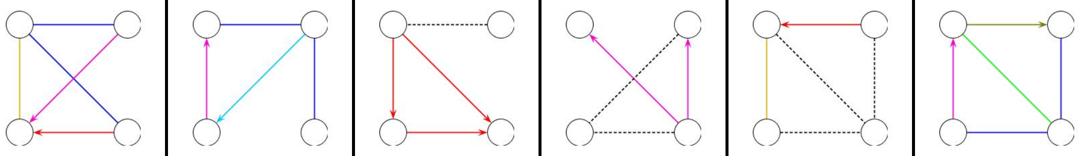

Motif # 168	Motif # 169	Motif # 170	Motif # 171	Motif # 172	Motif # 173
Num Real = 8	Num Real = 17	Num Real = 11	Num Real = 11	Num Real = 8	Num Real = 5

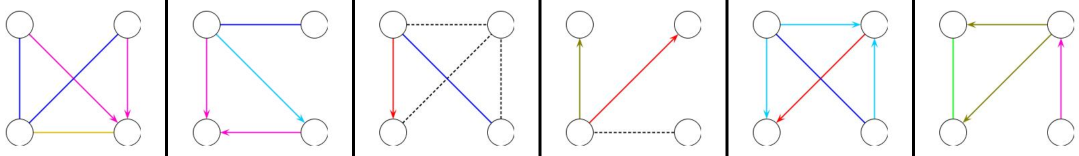

Motif # 176	Motif # 177	Motif # 179	Motif # 183	Motif # 184	Motif # 185
Num Real = 15	Num Real = 7	Num Real = 7	Num Real = 102	Num Real = 9	Num Real = 6

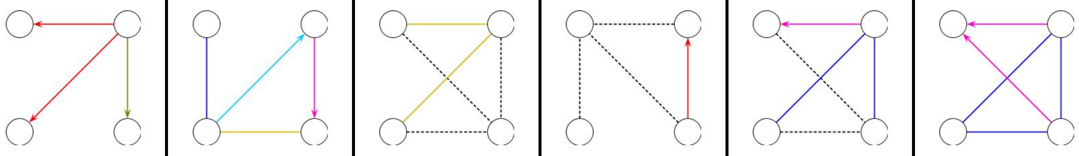

Motif # 190	Motif # 195	Motif # 196	Motif # 199	Motif # 200	Motif # 201
Num Real = 13	Num Real = 7	Num Real = 5	Num Real = 48	Num Real = 25	Num Real = 11

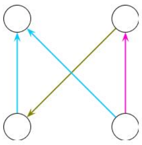	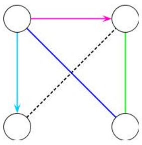	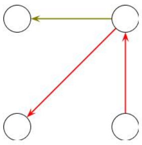	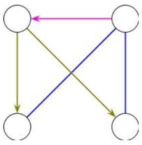	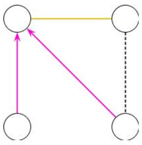	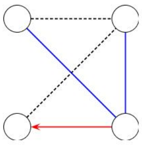
Motif # 202	Motif # 206	Motif # 209	Motif # 210	Motif # 212	Motif # 217
Num Real = 21	Num Real = 23	Num Real = 21	Num Real = 8	Num Real = 8	Num Real = 5
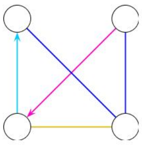	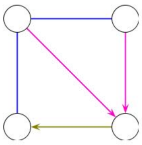	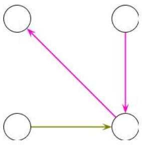	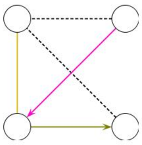	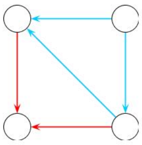	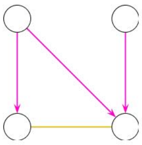
Motif # 218	Motif # 221	Motif # 223	Motif # 227	Motif # 228	Motif # 230
Num Real = 8	Num Real = 21	Num Real = 58	Num Real = 7	Num Real = 10	Num Real = 5
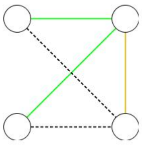	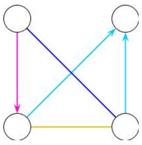	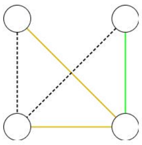	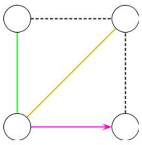	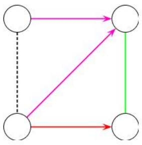	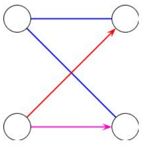
Motif # 232	Motif # 234	Motif # 239	Motif # 245	Motif # 252	Motif # 254
Num Real = 7	Num Real = 10	Num Real = 5	Num Real = 6	Num Real = 11	Num Real = 18
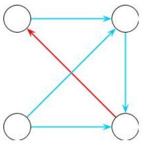	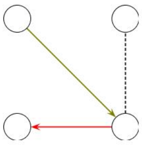	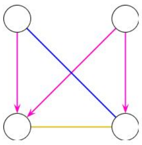	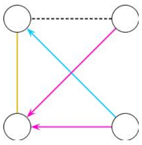		
Motif # 261	Motif # 272	Motif # 274	Motif # 306		
Num Real = 10	Num Real = 8	Num Real = 8	Num Real = 6		
