# Supplementary material for: Network motif analysis of a multi-mode genetic-interaction network
Source: Genome Biol. 2007 Aug 2;8(8):R160. doi: 10.1186/gb-2007-8-8-r160 (PMC2374991; doi:10.1186/gb-2007-8-8-r160)
Supplement: Additional data file 6 — Random distribution, parametric fit, and significance of the top 100 significant 4-node network patterns found in the genetic network. [file gb-2007-8-8-r160-S6.pdf]

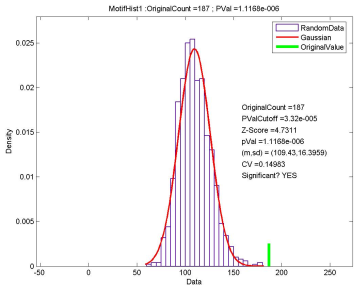

Motif # 1

zScore = 4.7311

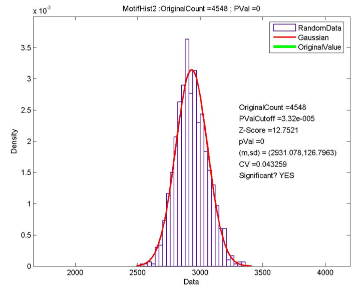

Motif # 2

zScore = 12.7521

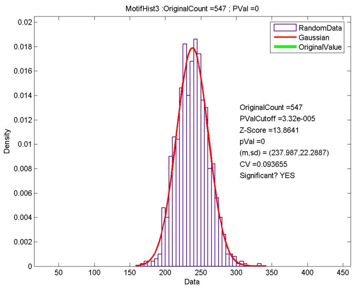

Motif # 3

zScore = 13.8641

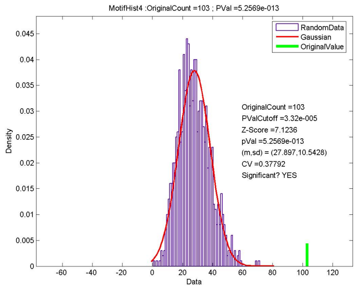

Motif # 4

zScore = 7.1236

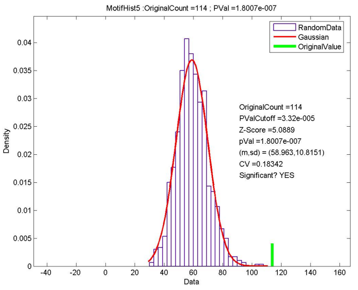

Motif # 5

zScore = 5.0889

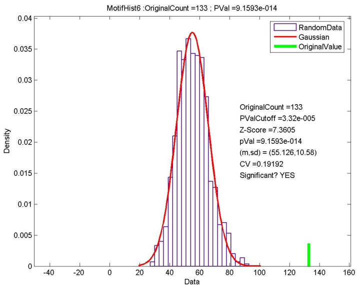

Motif # 6

zScore = 7.3605

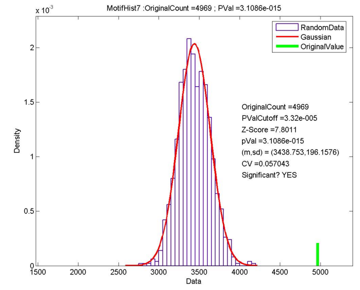

Motif # 7

zScore = 7.8011

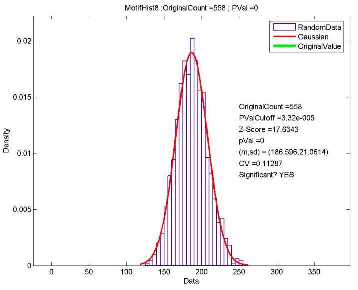

Motif # 8

zScore = 17.6343

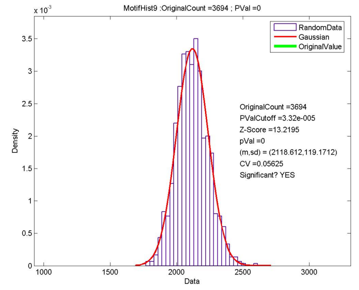

Motif # 9

zScore = 13.2195

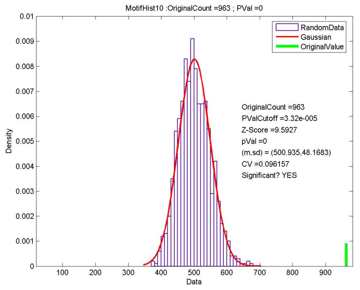

Motif # 10

zScore = 9.5927

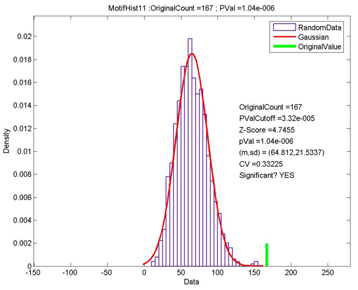

Motif # 11

zScore = 4.7455

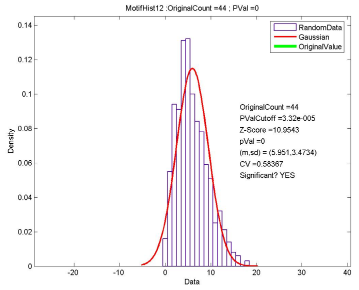

Motif # 12

zScore = 10.9543

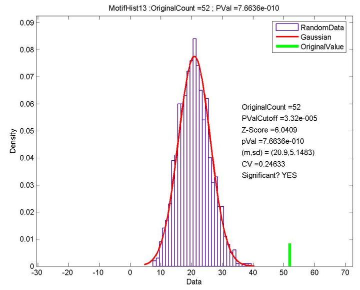

Motif # 13

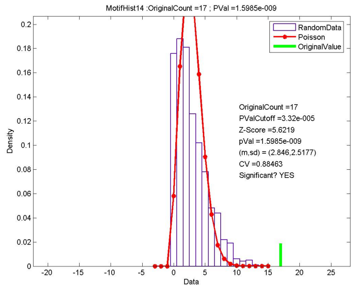

Motif # 14

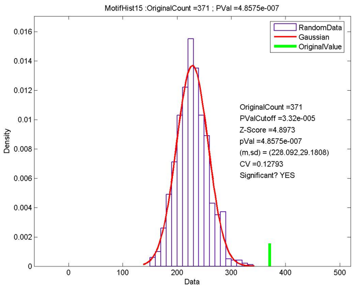

Motif # 15

zScore = 6.0409	zScore = 5.6219	zScore = 4.8973

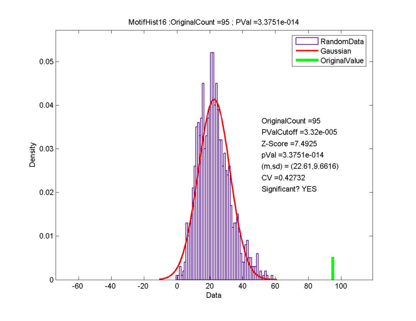

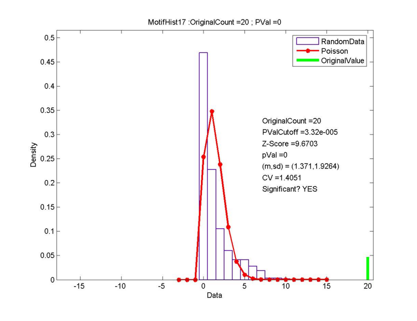

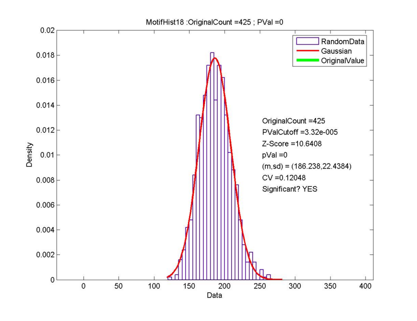

Motif # 16	Motif # 17	Motif # 18

zScore = 7.4925	zScore = 9.6703	zScore = 10.6408

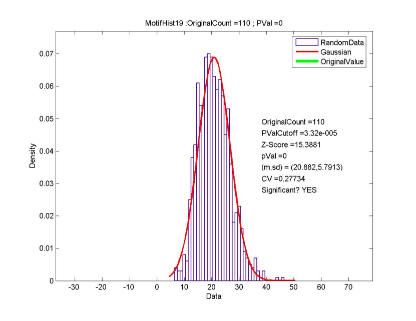

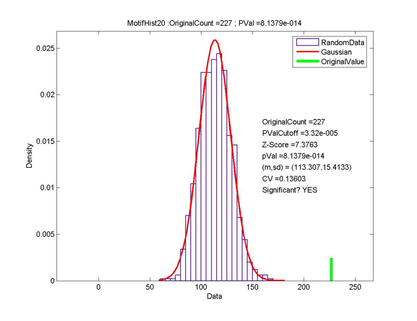

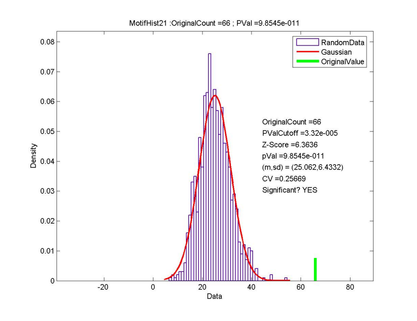

Motif # 19	Motif # 20	Motif # 21

zScore = 15.3881	zScore = 7.3763	zScore = 6.3636

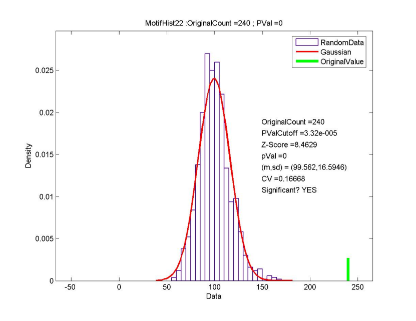

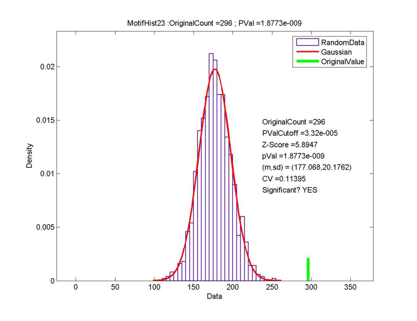

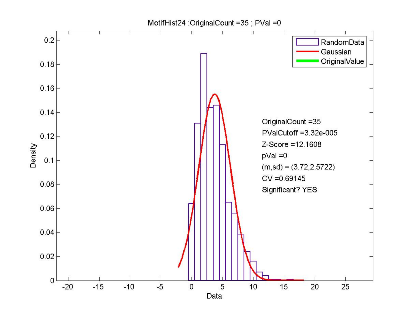

Motif # 22	Motif # 23	Motif # 24

zScore = 8.4629	zScore = 5.8947	zScore = 12.1608

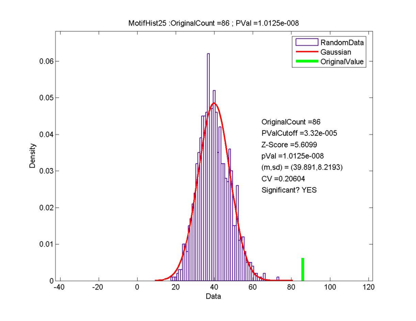

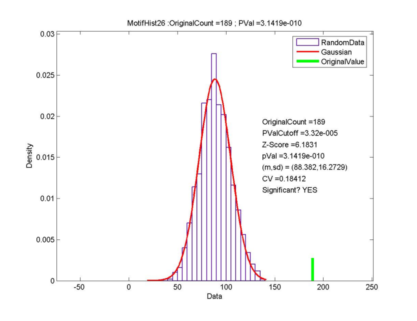

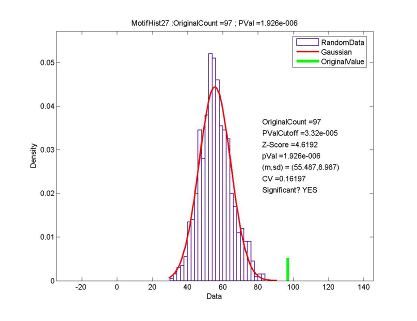

Motif # 25	Motif # 26	Motif # 27

zScore = 5.6099	zScore = 6.1831	zScore = 4.6192

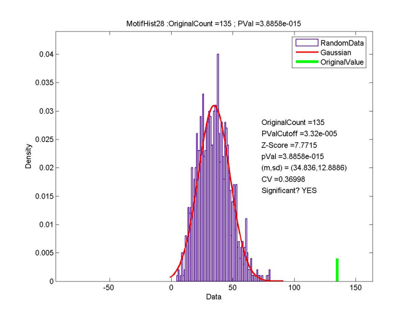

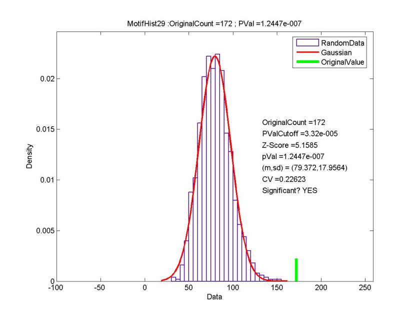

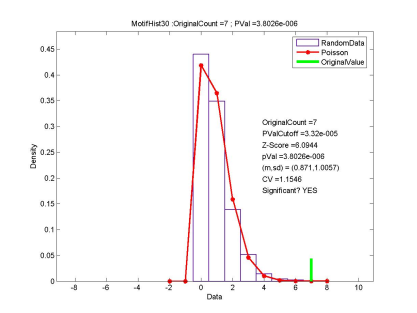

Motif # 28	Motif # 29	Motif # 30
zScore = 7.7715	zScore = 5.1585	zScore = 6.0944

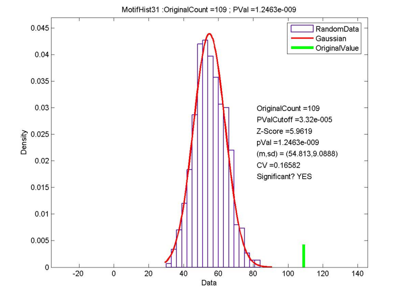

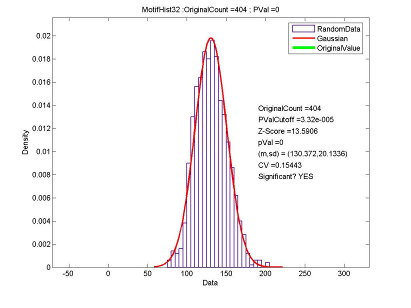

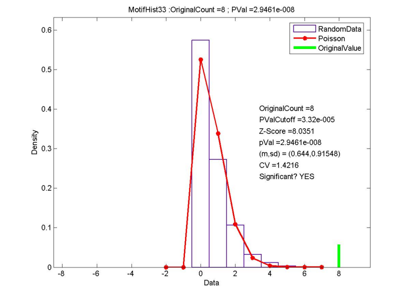

Motif # 31	Motif # 32	Motif # 33
zScore = 5.9619	zScore = 13.5906	zScore = 8.0351

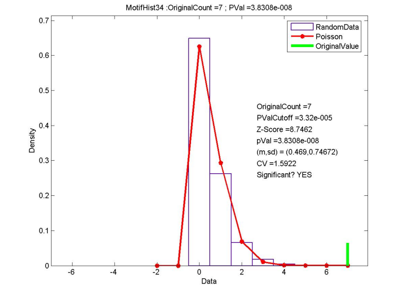

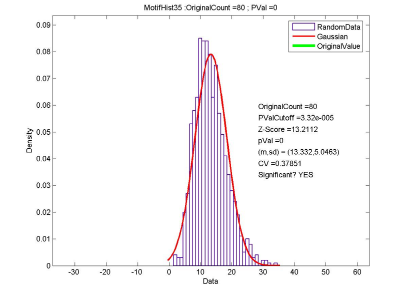

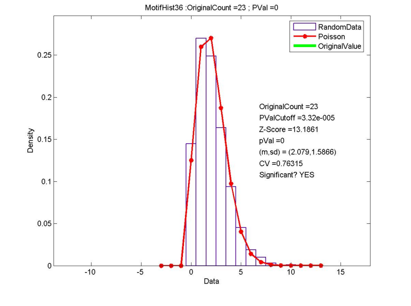

Motif # 34	Motif # 35	Motif # 36
zScore = 8.7462	zScore = 13.2112	zScore = 13.1861

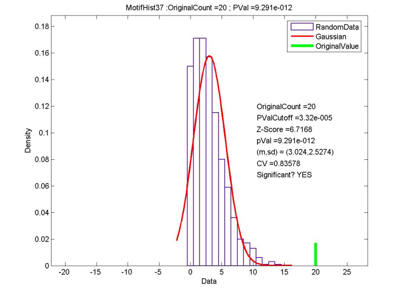

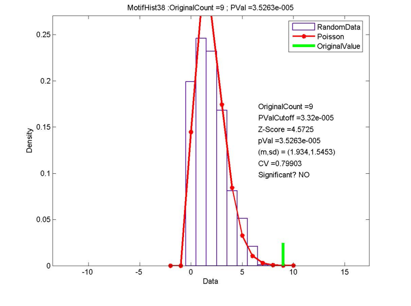

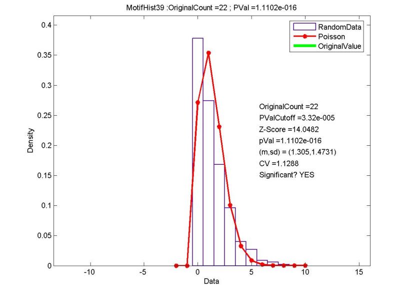

Motif # 37	Motif # 38	Motif # 39
zScore = 6.7168	zScore = 4.5725	zScore = 14.0482

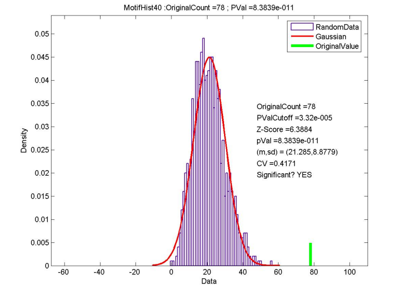

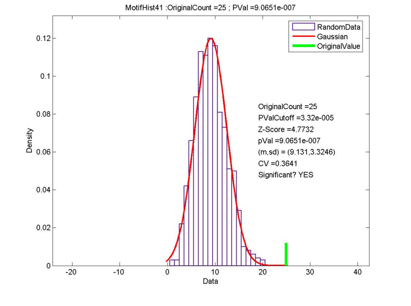

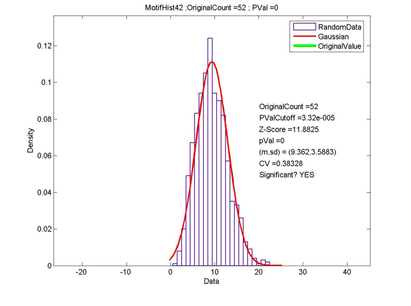

Motif # 40	Motif # 41	Motif # 42
zScore = 6.3884	zScore = 4.7732	zScore = 11.8825

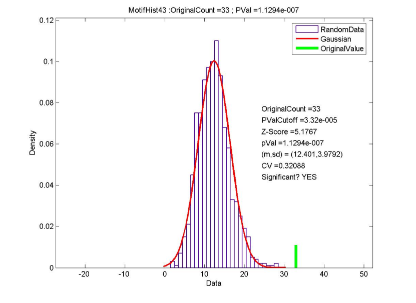

Motif # 43

zScore = 5.1767

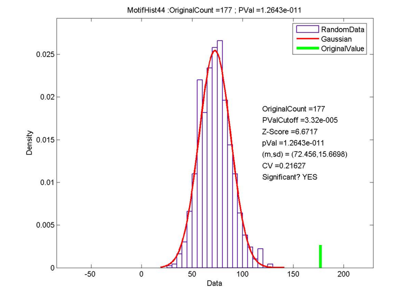

Motif # 44

zScore = 6.6717

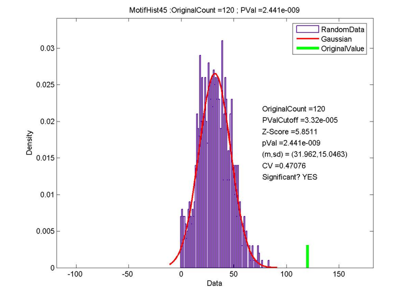

Motif # 45

zScore = 5.8511

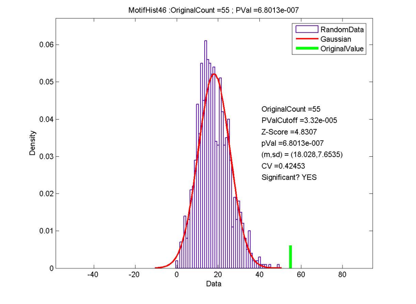

Motif # 46

zScore = 4.8307

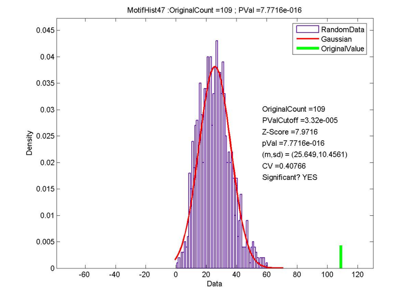

Motif # 47

zScore = 7.9716

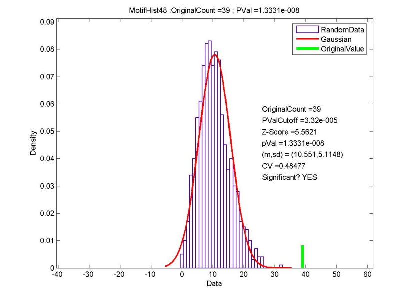

Motif # 48

zScore = 5.5621

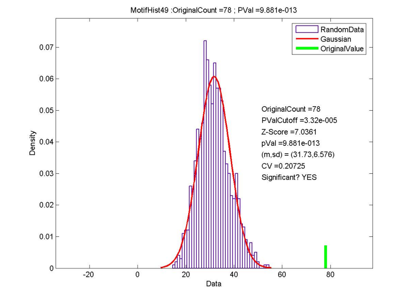

Motif # 49

zScore = 7.0361

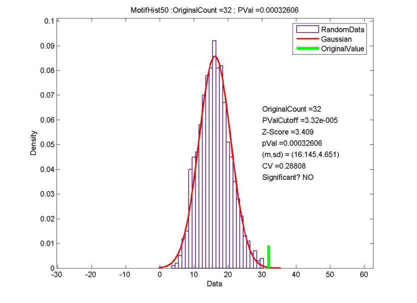

Motif # 50

zScore = 3.409

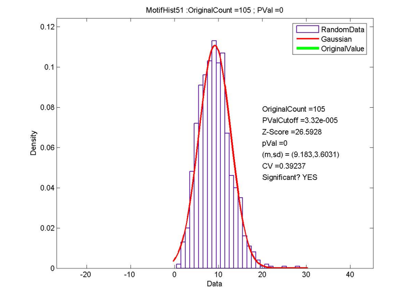

Motif # 51

zScore = 26.5928

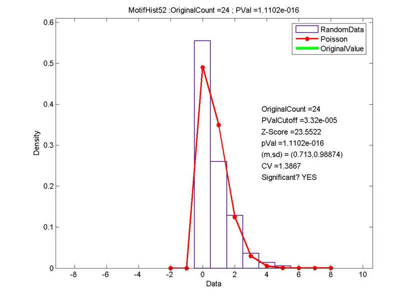

Motif # 52

zScore = 23.5522

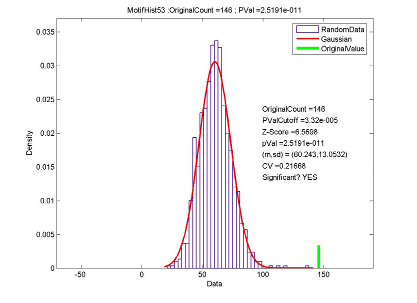

Motif # 53

zScore = 6.5698

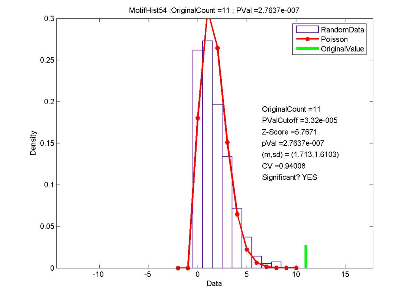

Motif # 54

zScore = 5.7671

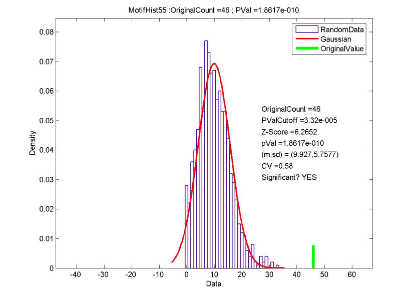

Motif # 55

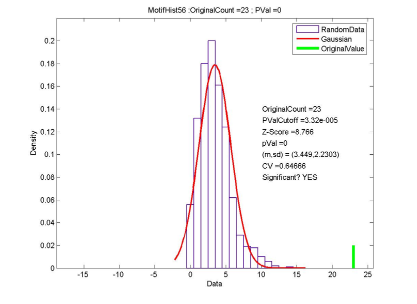

Motif # 56

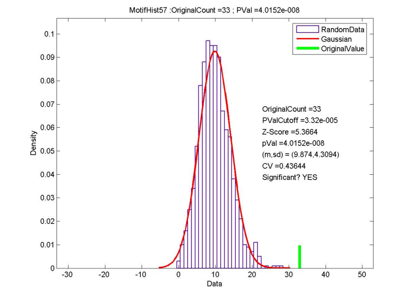

Motif # 57

zScore = 6.2652 | zScore = 8.766 | zScore = 5.3664

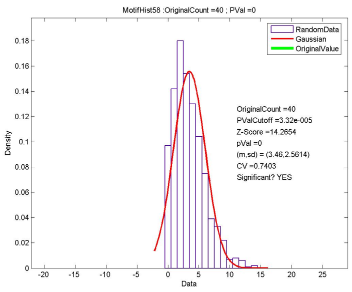

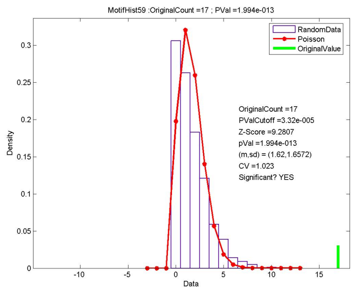

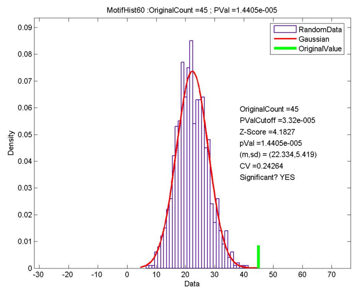

Motif # 58 | Motif # 59 | Motif # 60

zScore = 14.2654 | zScore = 9.2807 | zScore = 4.1827

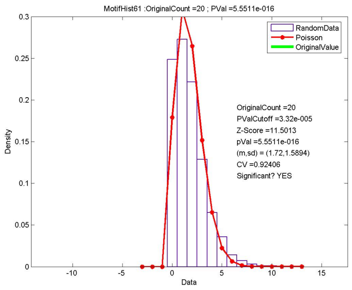

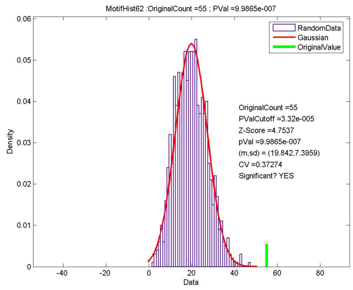

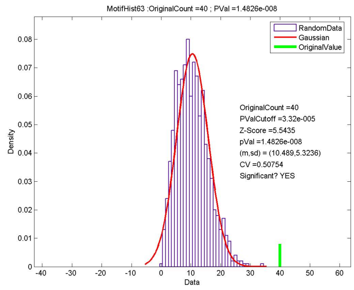

Motif # 61 | Motif # 62 | Motif # 63

zScore = 11.5013 | zScore = 4.7537 | zScore = 5.5435

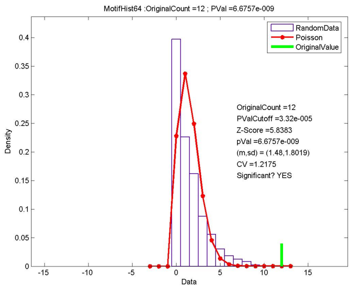

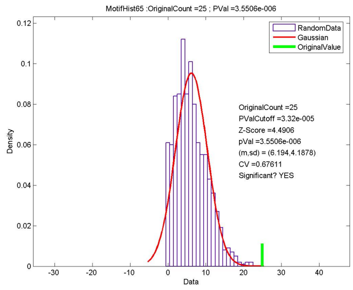

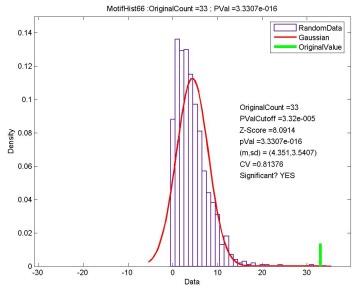

Motif # 64 | Motif # 65 | Motif # 66

zScore = 5.8383 | zScore = 4.4906 | zScore = 8.0914

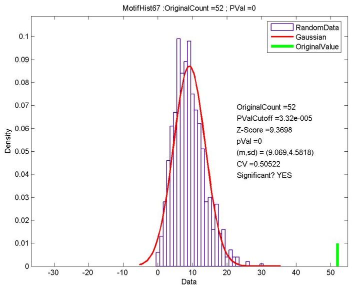

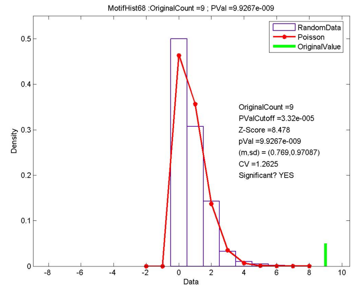

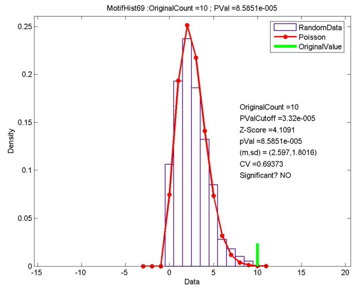

Motif # 67 | Motif # 68 | Motif # 69

zScore = 9.3698 | zScore = 8.478 | zScore = 4.1091

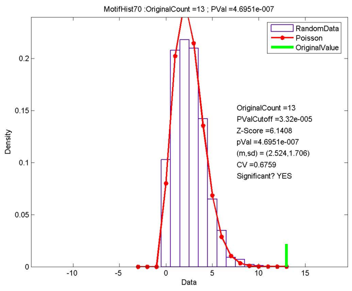

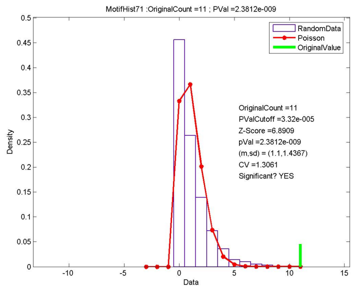

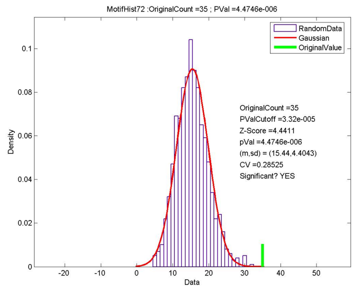

<table>
<tr><td>Motif # 70</td><td>Motif # 71</td><td>Motif # 72</td></tr>
<tr><td>zScore = 6.1408</td><td>zScore = 6.8909</td><td>zScore = 4.4411</td></tr>
<tr><td>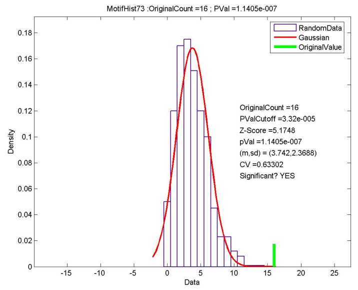
</td><td>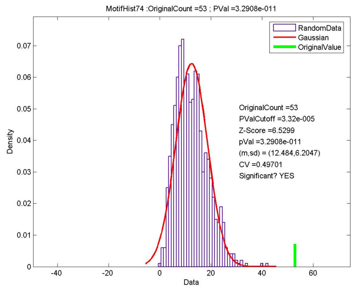
</td><td>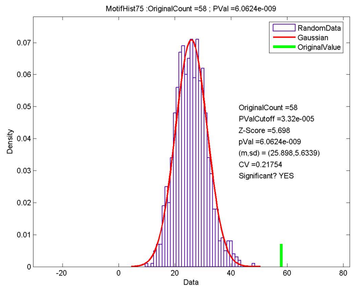
</td></tr>
<tr><td>Motif # 73</td><td>Motif # 74</td><td>Motif # 75</td></tr>
<tr><td>zScore = 5.1748</td><td>zScore = 6.5299</td><td>zScore = 5.698</td></tr>
<tr><td>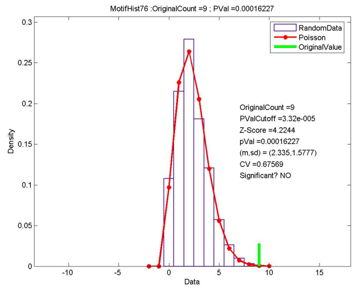
</td><td>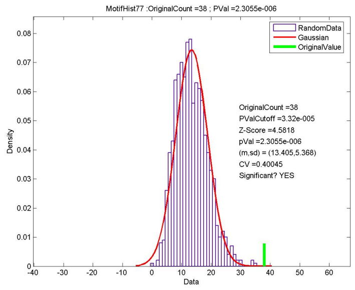
</td><td>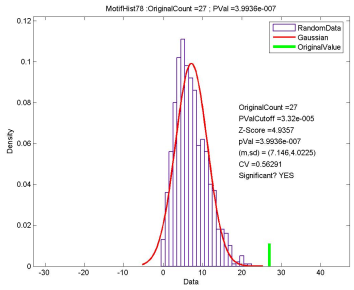
</td></tr>
<tr><td>Motif # 76</td><td>Motif # 77</td><td>Motif # 78</td></tr>
<tr><td>zScore = 4.2244</td><td>zScore = 4.5818</td><td>zScore = 4.9357</td></tr>
<tr><td>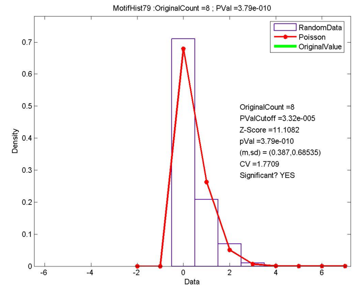
</td><td>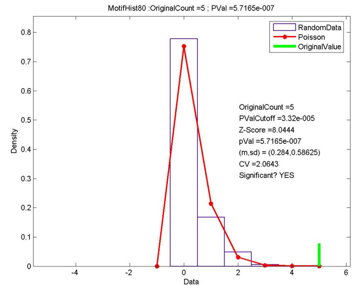
</td><td>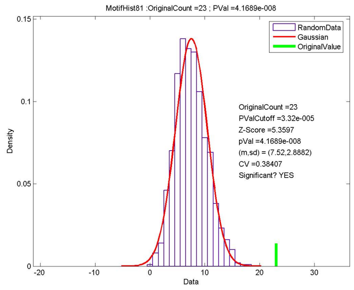
</td></tr>
<tr><td>Motif # 79</td><td>Motif # 80</td><td>Motif # 81</td></tr>
<tr><td>zScore = 11.1082</td><td>zScore = 8.0444</td><td>zScore = 5.3597</td></tr>
<tr><td>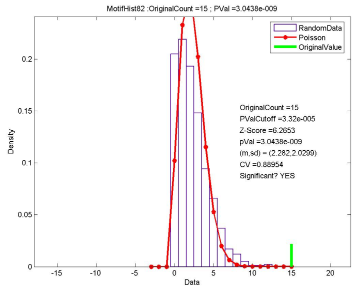
</td><td>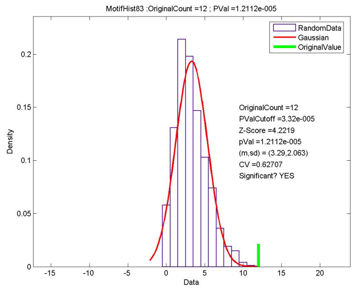
</td><td>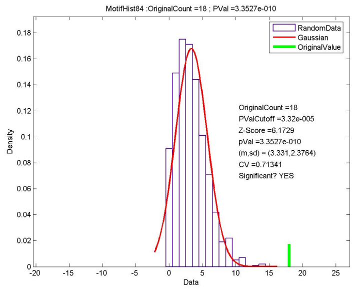
</td></tr>
<tr><td>Motif # 82</td><td>Motif # 83</td><td>Motif # 84</td></tr>
<tr><td>zScore = 6.2653</td><td>zScore = 4.2219</td><td>zScore = 6.1729</td></tr>
</table>

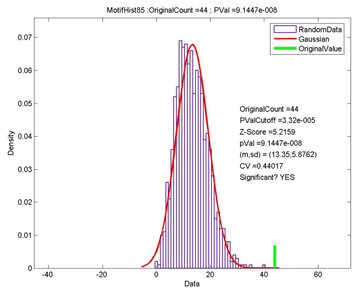

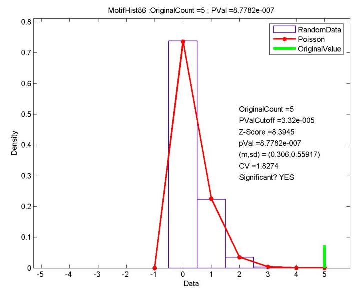

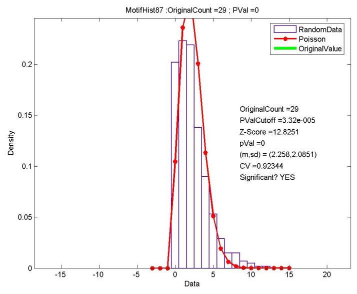

Motif # 85	Motif # 86	Motif # 87
zScore = 5.2159	zScore = 8.3945	zScore = 12.8251

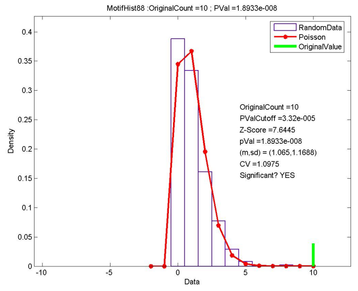

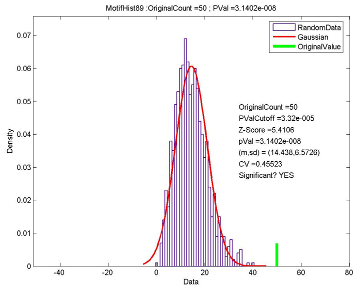

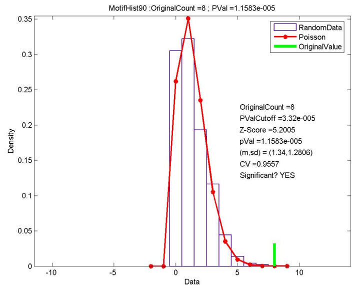

Motif # 88	Motif # 89	Motif # 90
zScore = 7.6445	zScore = 5.4106	zScore = 5.2005

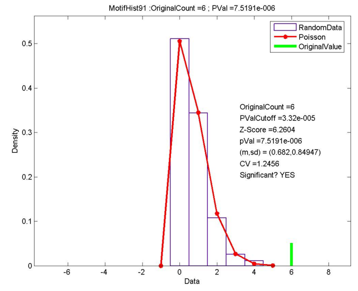

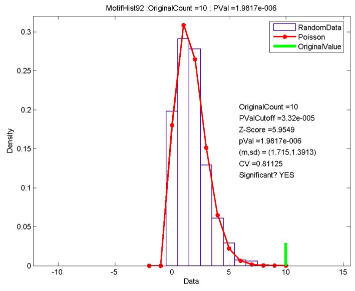

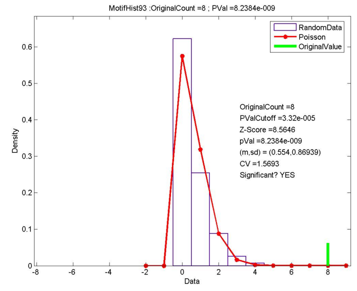

Motif # 91	Motif # 92	Motif # 93
zScore = 6.2604	zScore = 5.9549	zScore = 8.5646

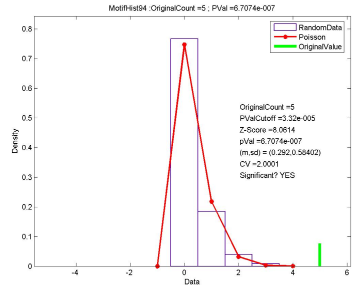

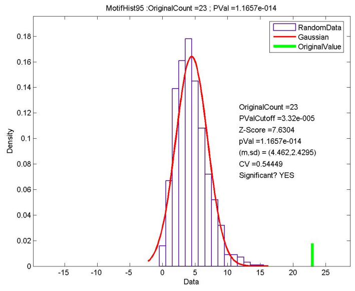

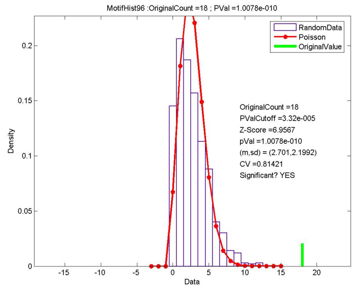

Motif # 94	Motif # 95	Motif # 96
zScore = 8.0614	zScore = 7.6304	zScore = 6.9567

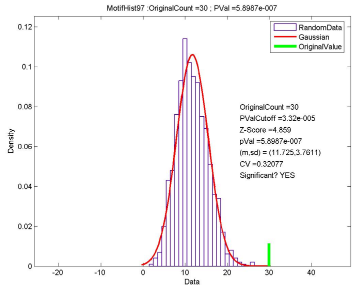

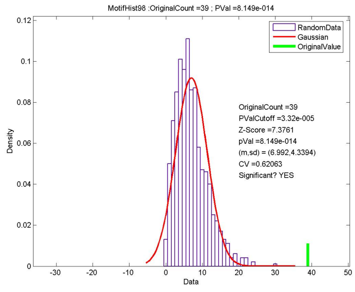

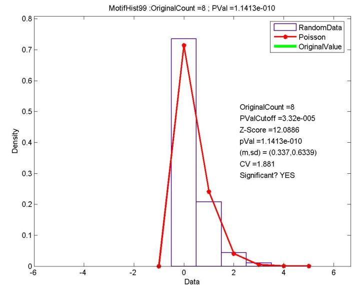

Motif # 97	Motif # 98	Motif # 99

zScore = 4.859	zScore = 7.3761	zScore = 12.0886

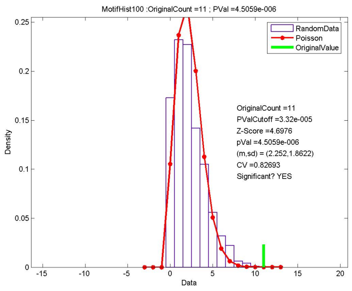

Motif # 100

zScore = 4.6976
