# Supplementary figures and images for: Network motif analysis of a multi-mode genetic-interaction network
Source: Genome Biol. 2007 Aug 2;8(8):R160. doi: 10.1186/gb-2007-8-8-r160 (PMC2374991; doi:10.1186/gb-2007-8-8-r160)

|                                                                                   |                                                                                   |                                                                                   |                                                                                   |                                                                                    |                                                                                     |
|-----------------------------------------------------------------------------------|-----------------------------------------------------------------------------------|-----------------------------------------------------------------------------------|-----------------------------------------------------------------------------------|------------------------------------------------------------------------------------|-------------------------------------------------------------------------------------|
| 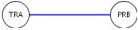 | 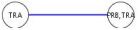 | 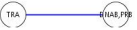 | 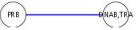 | 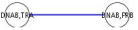 | 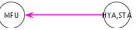 |
| Motif # 1                                                                         | Motif # 5                                                                         | Motif # 8                                                                         | Motif # 9                                                                         | Motif # 13                                                                         | Motif # 14                                                                          |
| Num Real = 32                                                                     | Num Real = 73                                                                     | Num Real = 56                                                                     | Num Real = 32                                                                     | Num Real = 56                                                                      | Num Real = 12                                                                       |

Supplement: Additional data file 7 — Full collection of 2nGO-motifs. [file gb-2007-8-8-r160-S7.pdf]

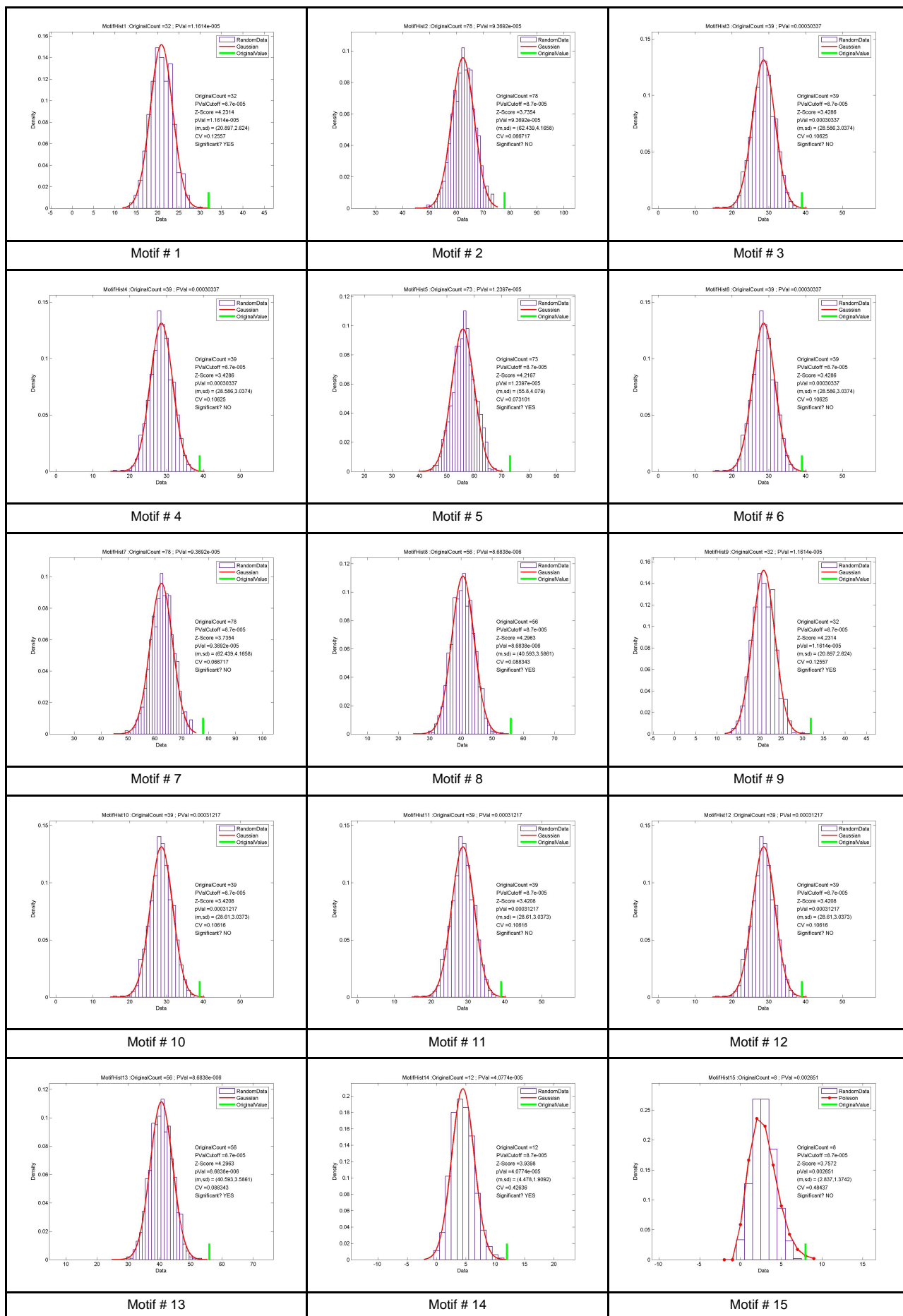

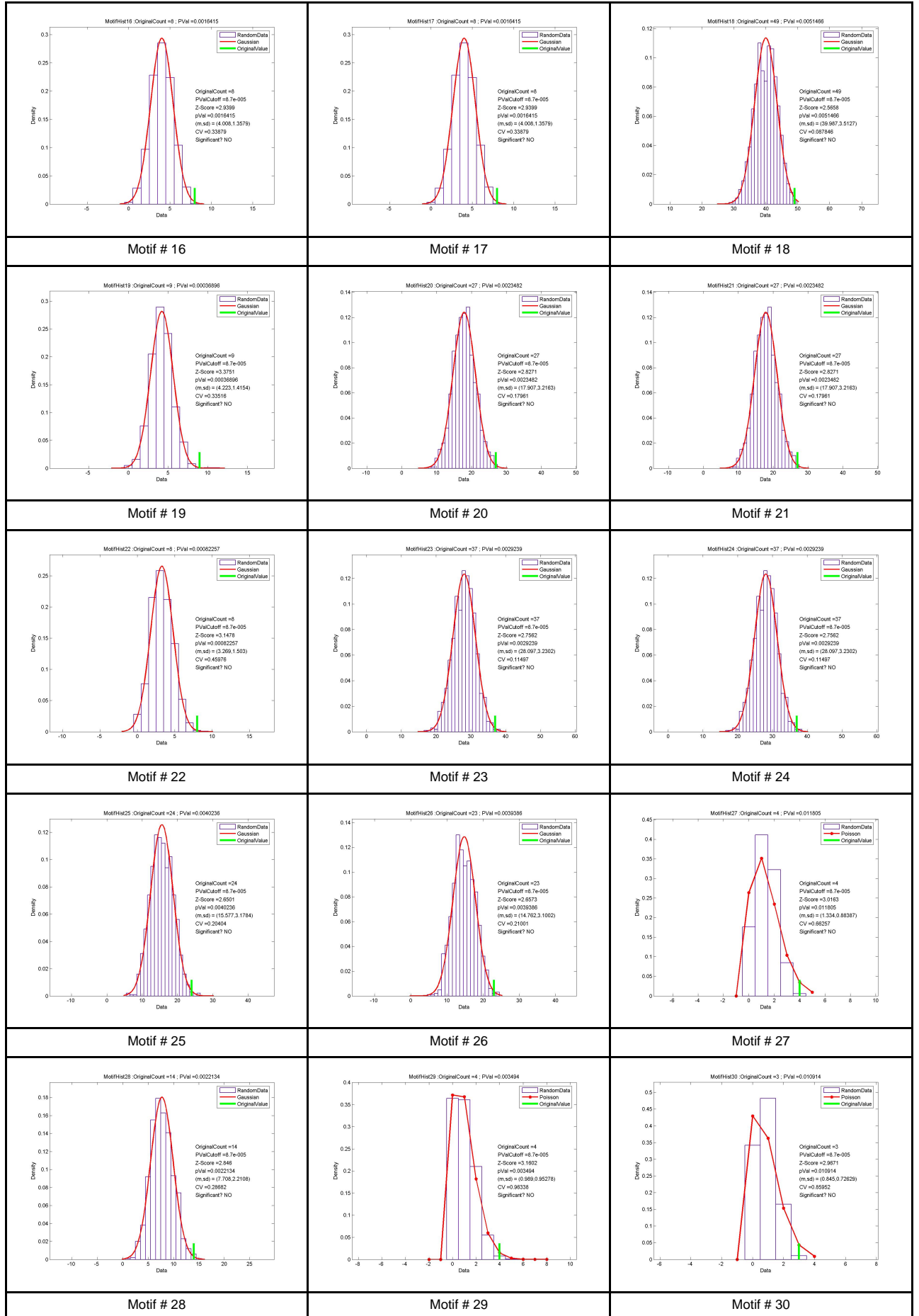

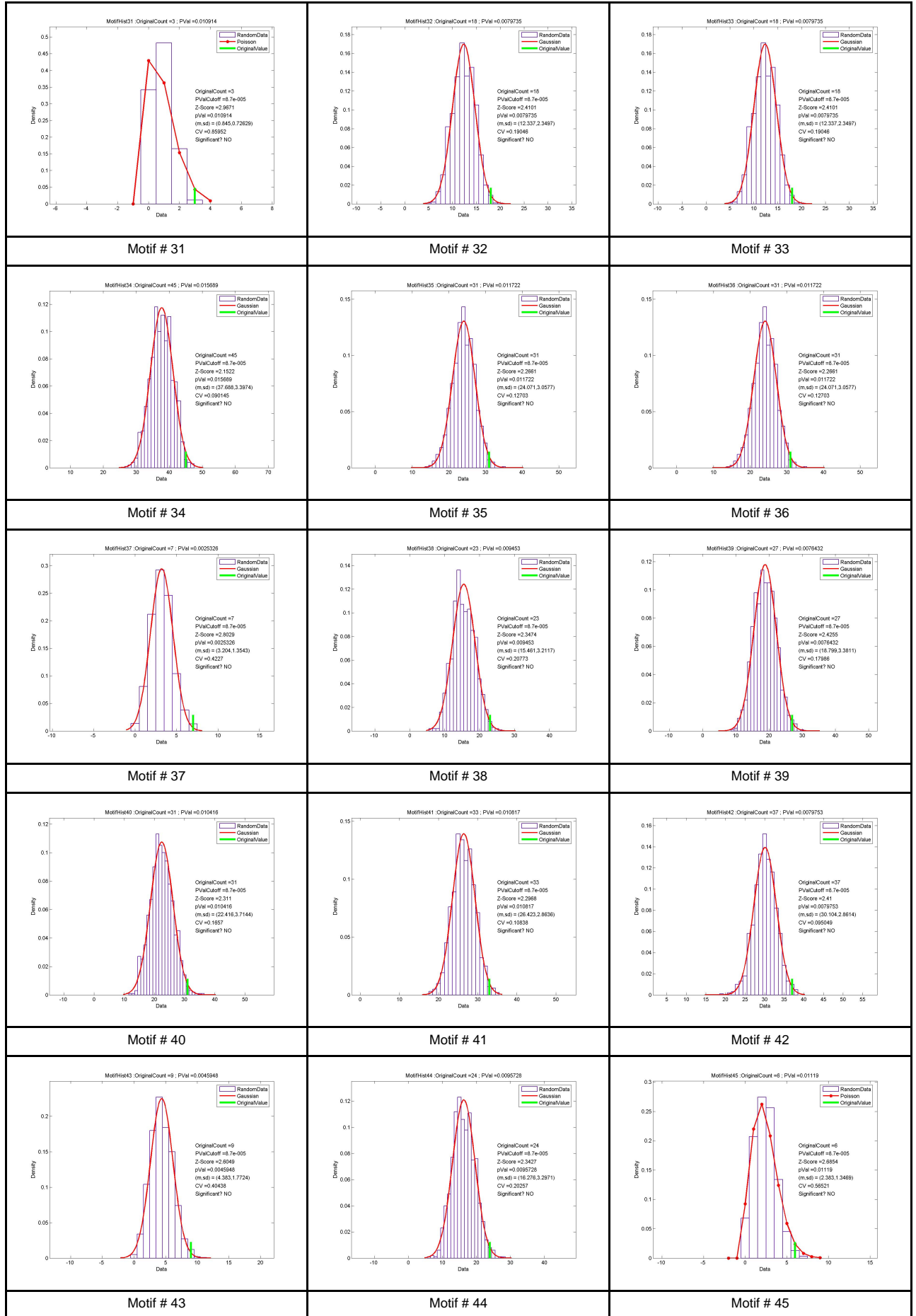

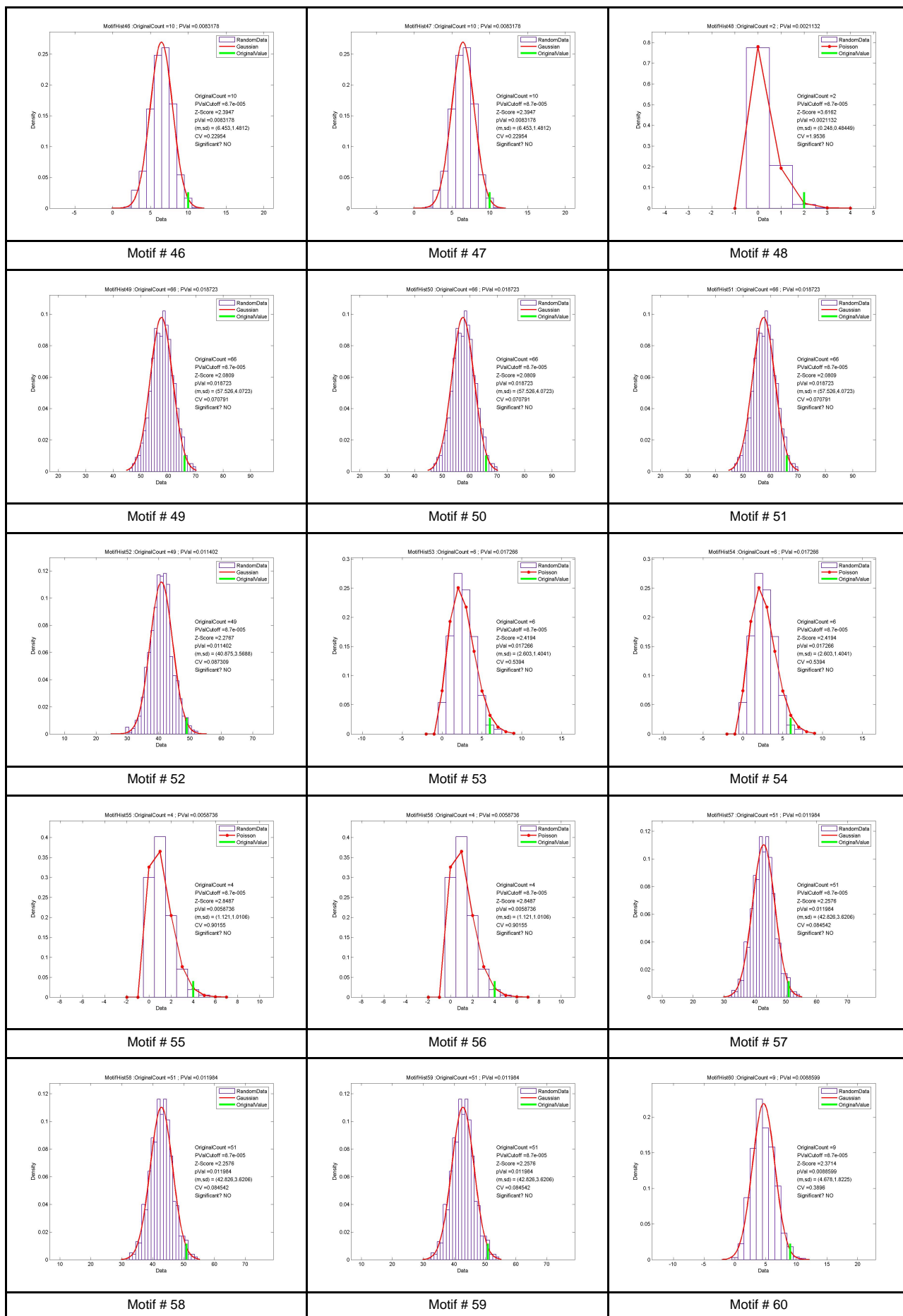

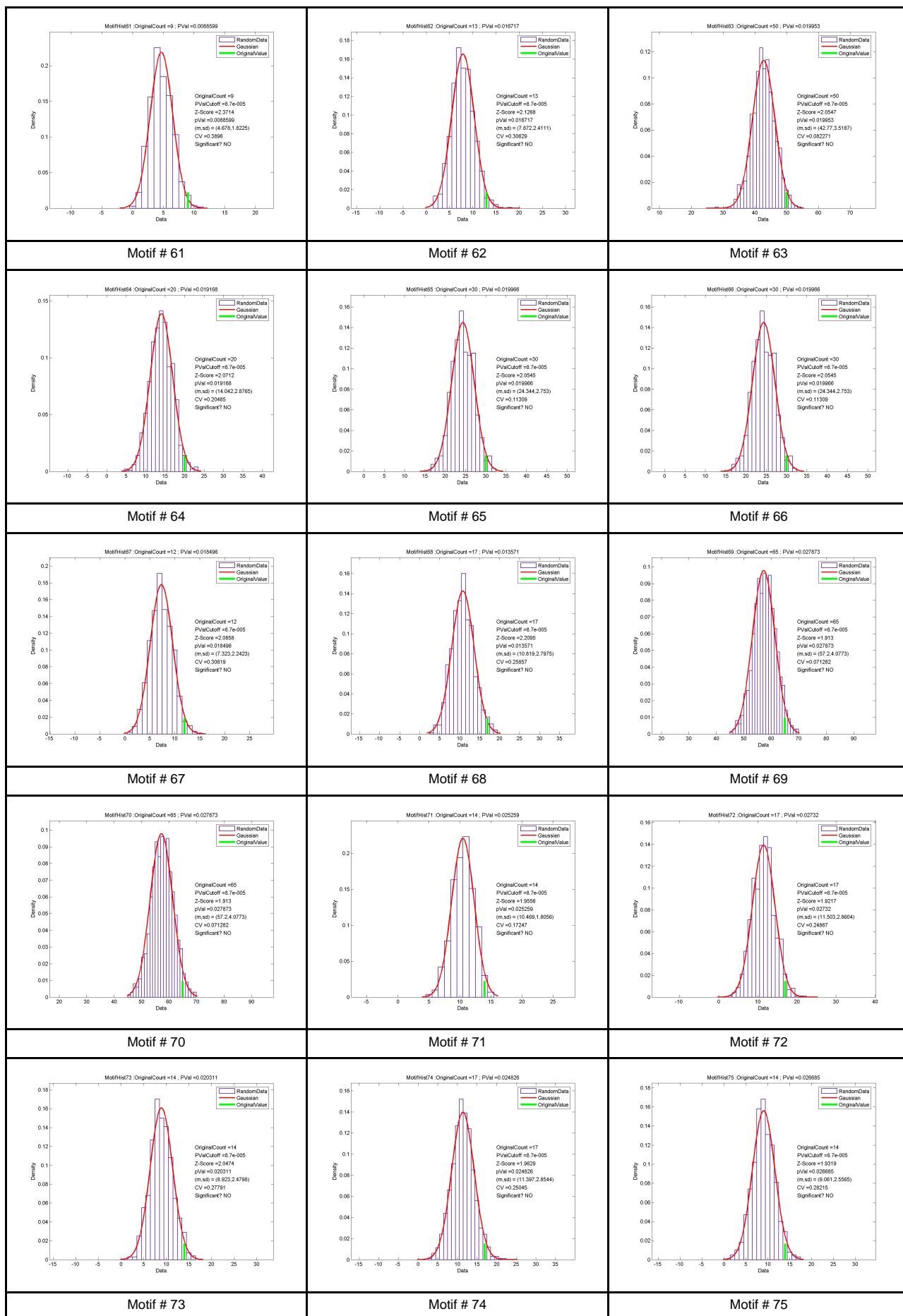

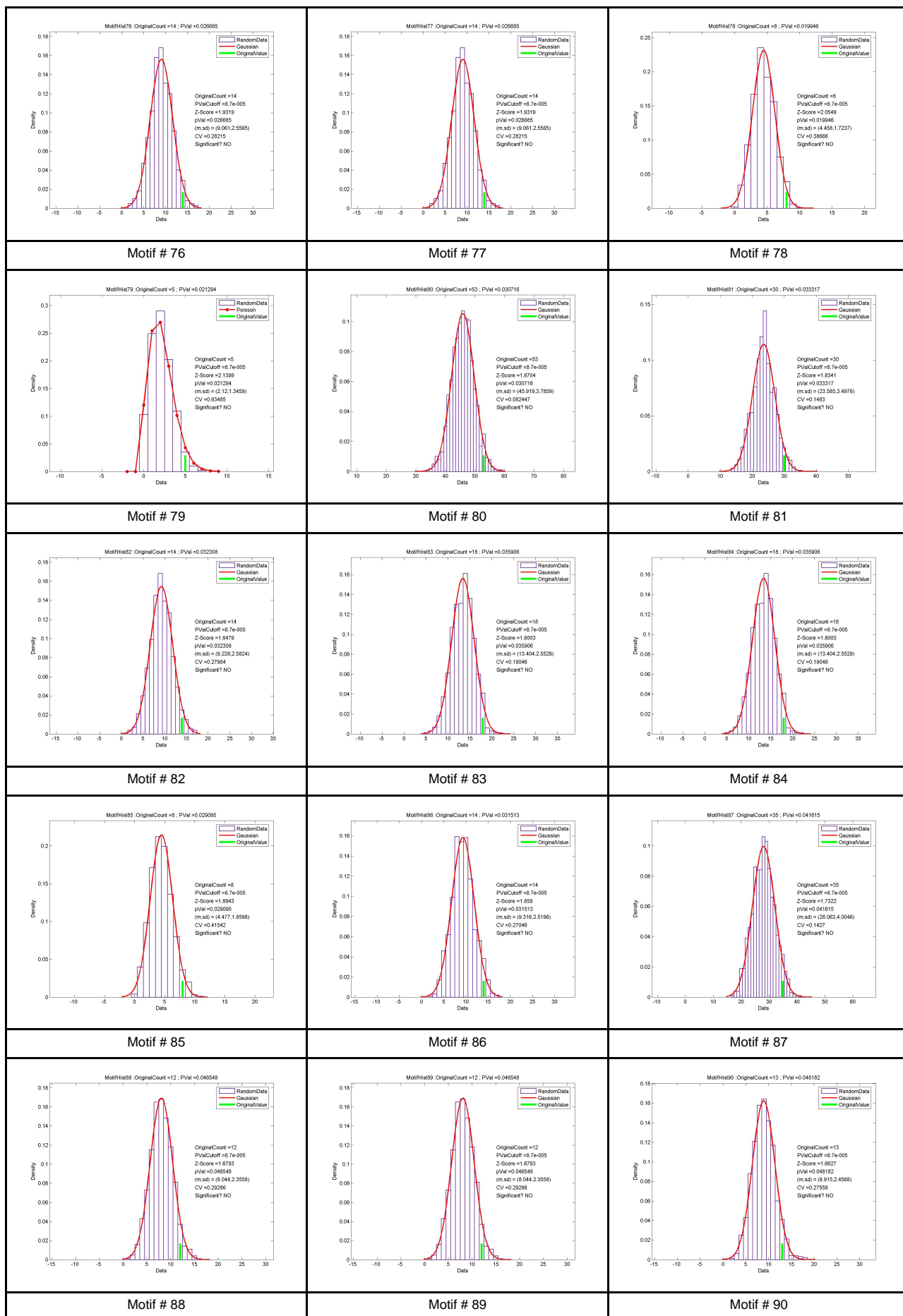

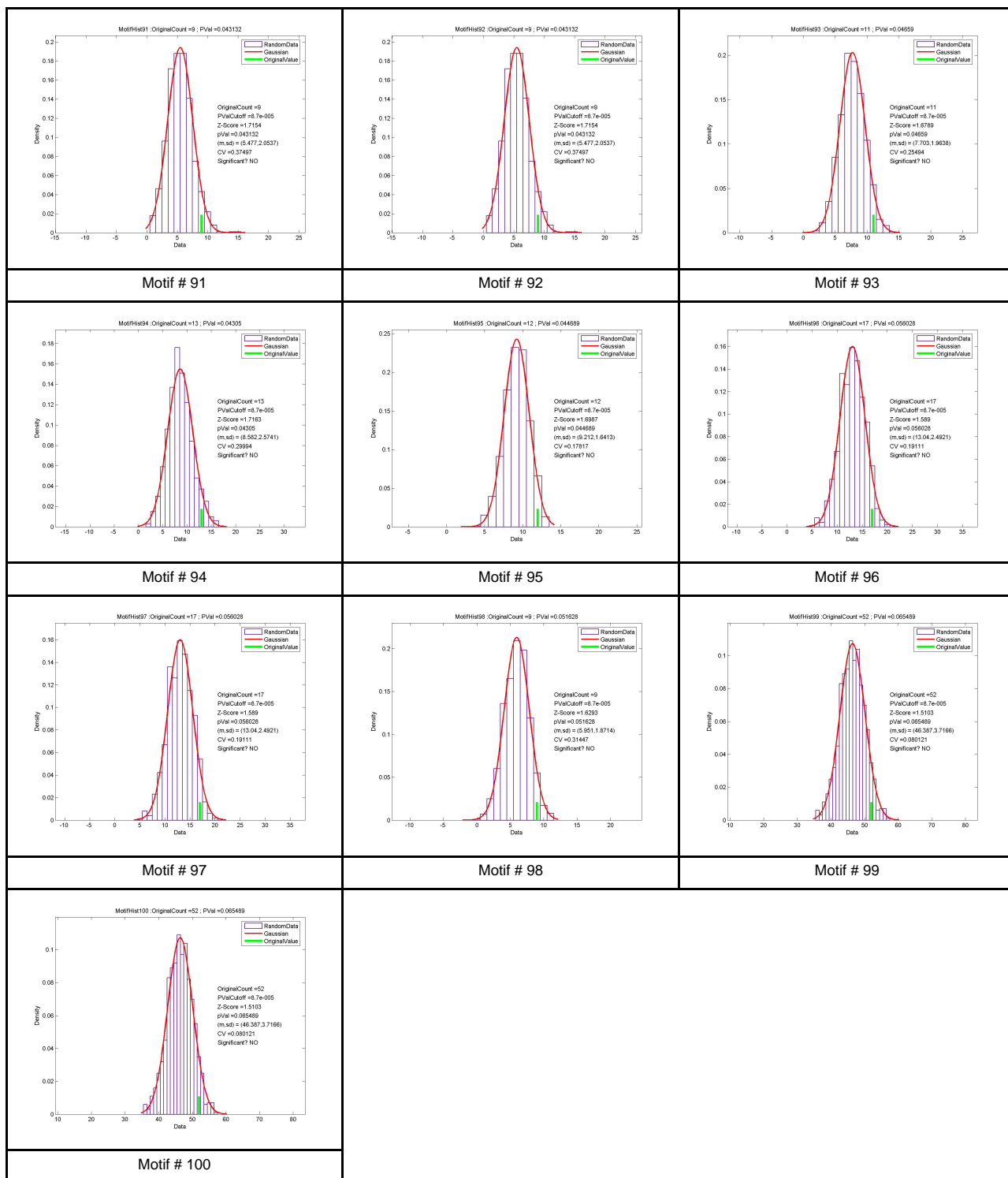

Supplement: Additional data file 9 — Random distribution, parametric fit, and significance of the top 100 significant 2nGO network patterns found in the genetic network. [file gb-2007-8-8-r160-S9.pdf]

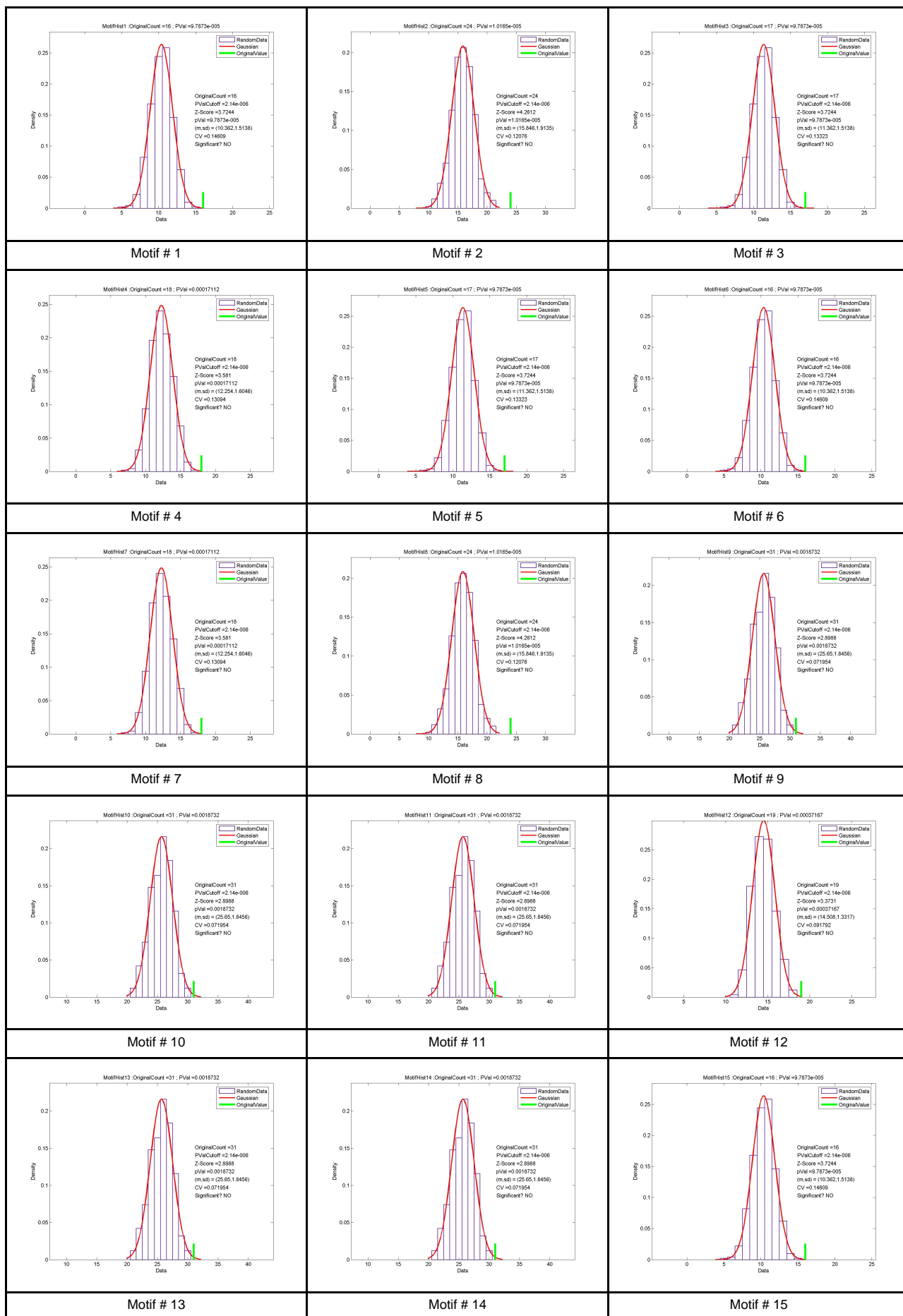

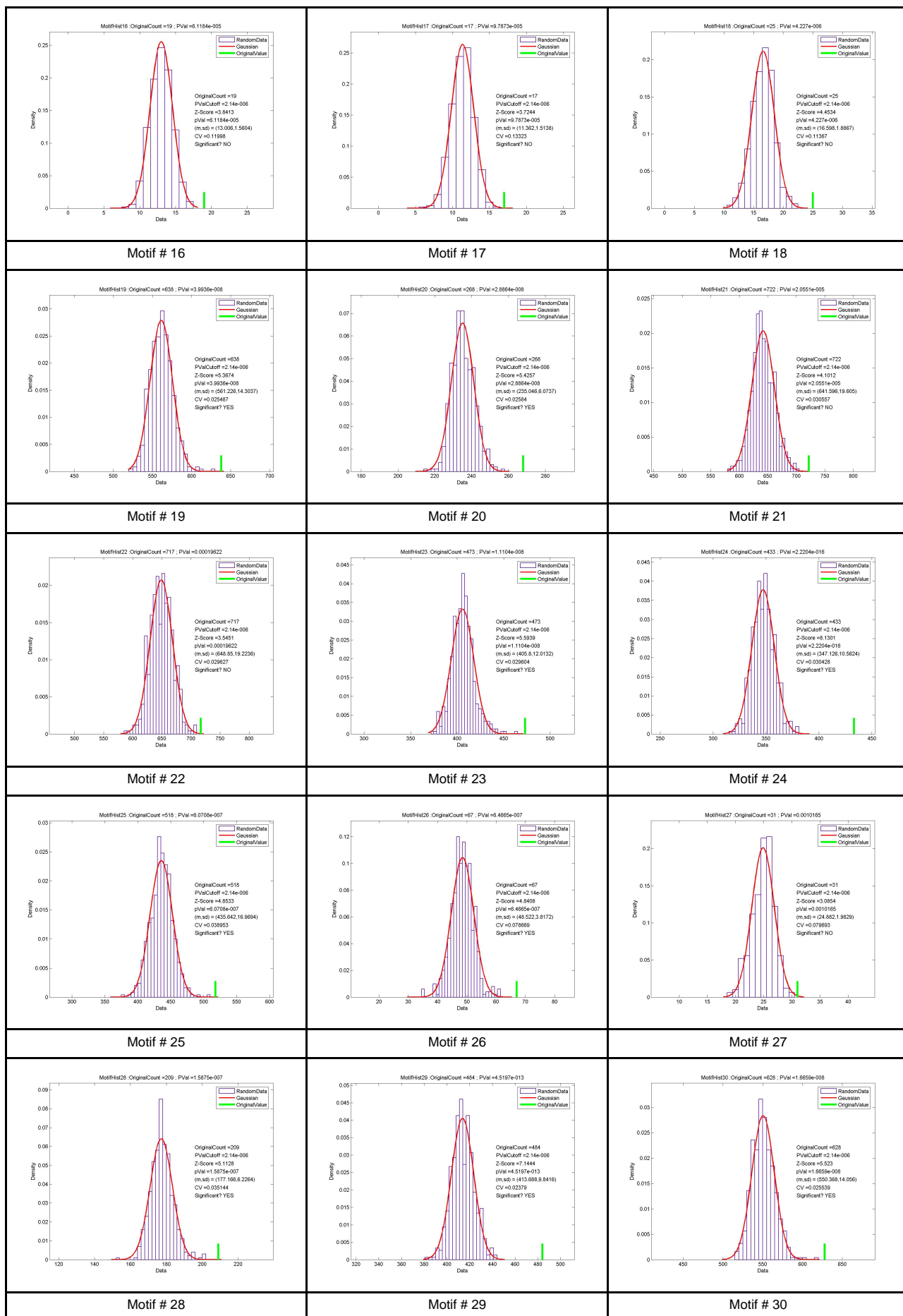

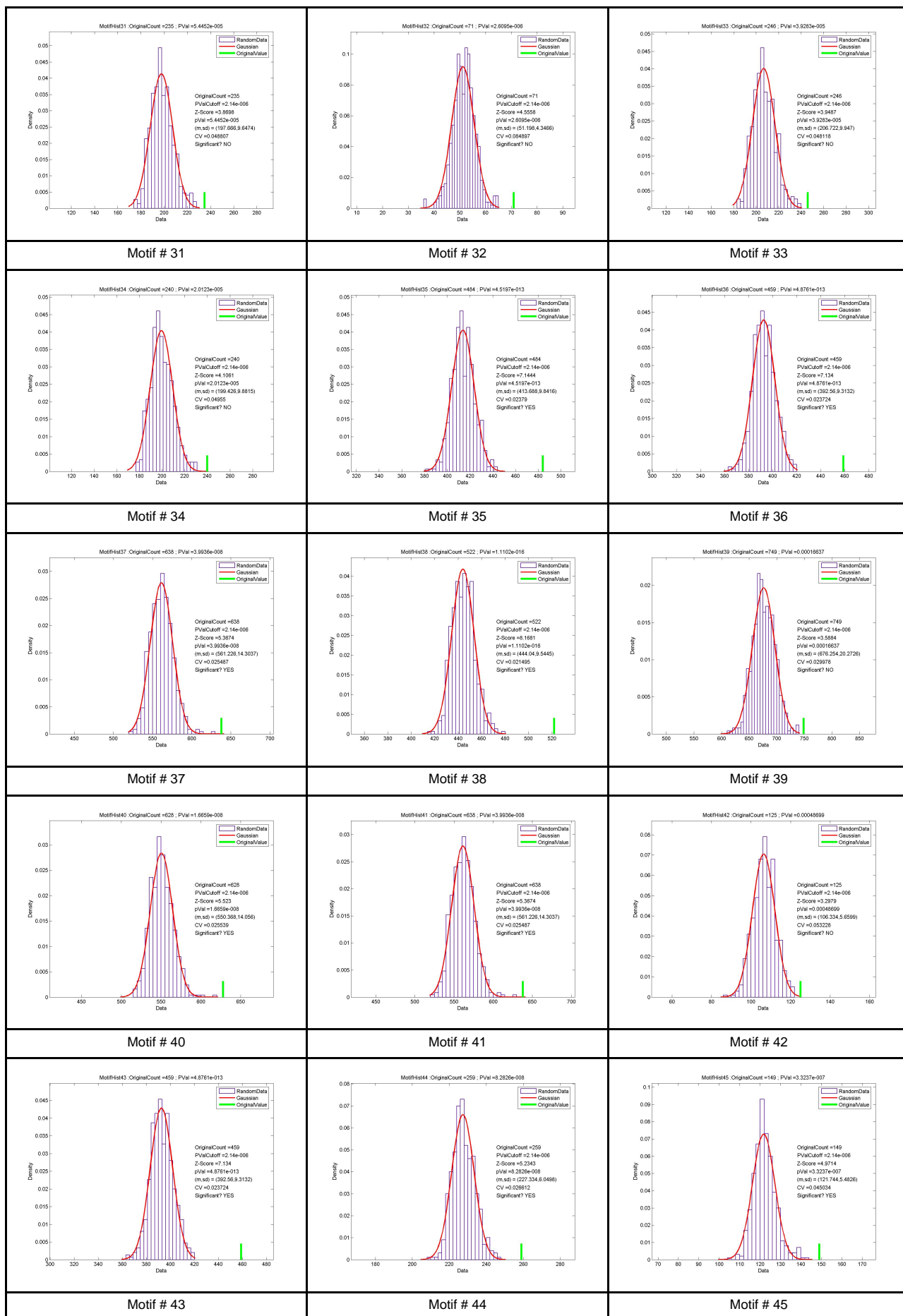

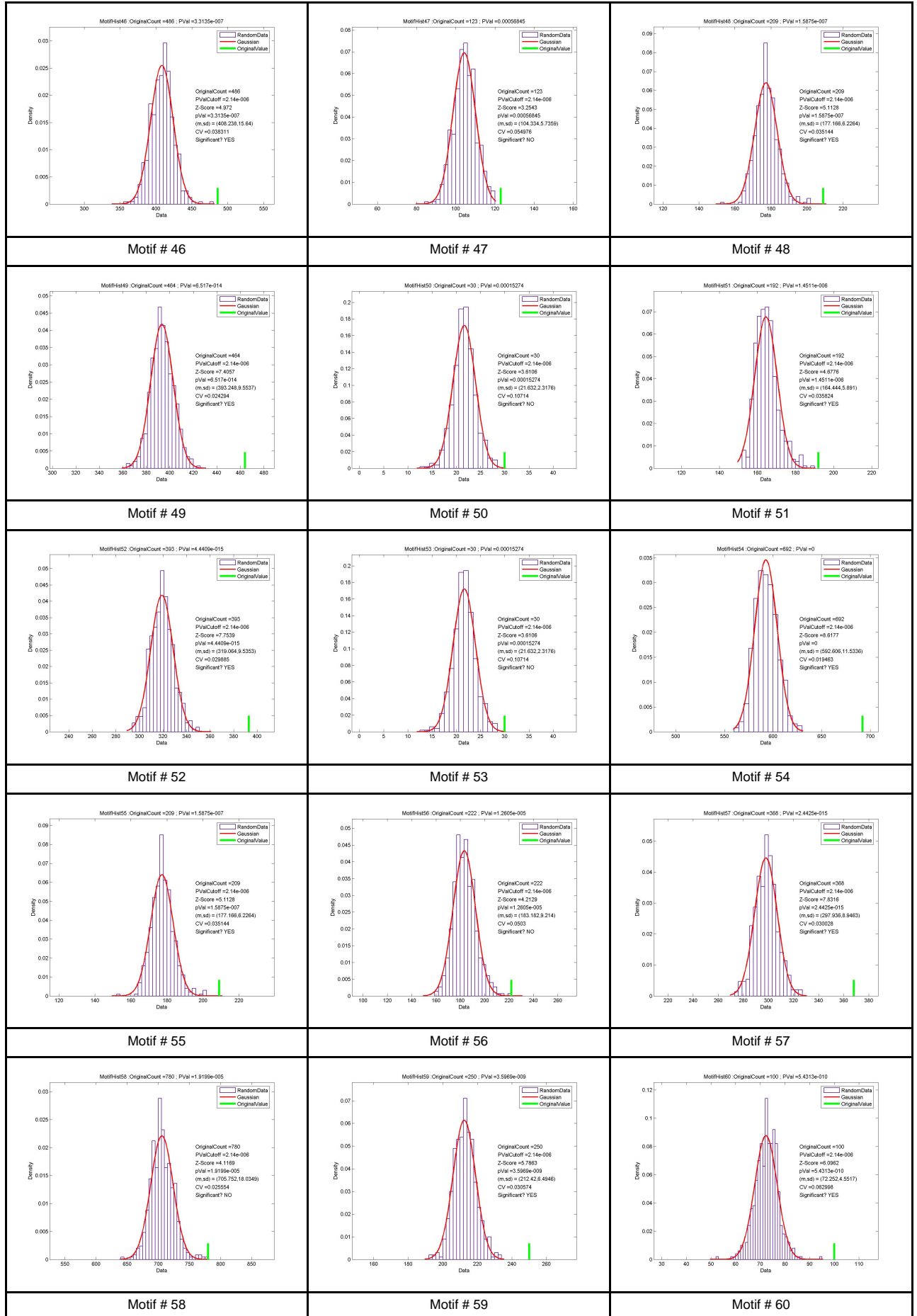

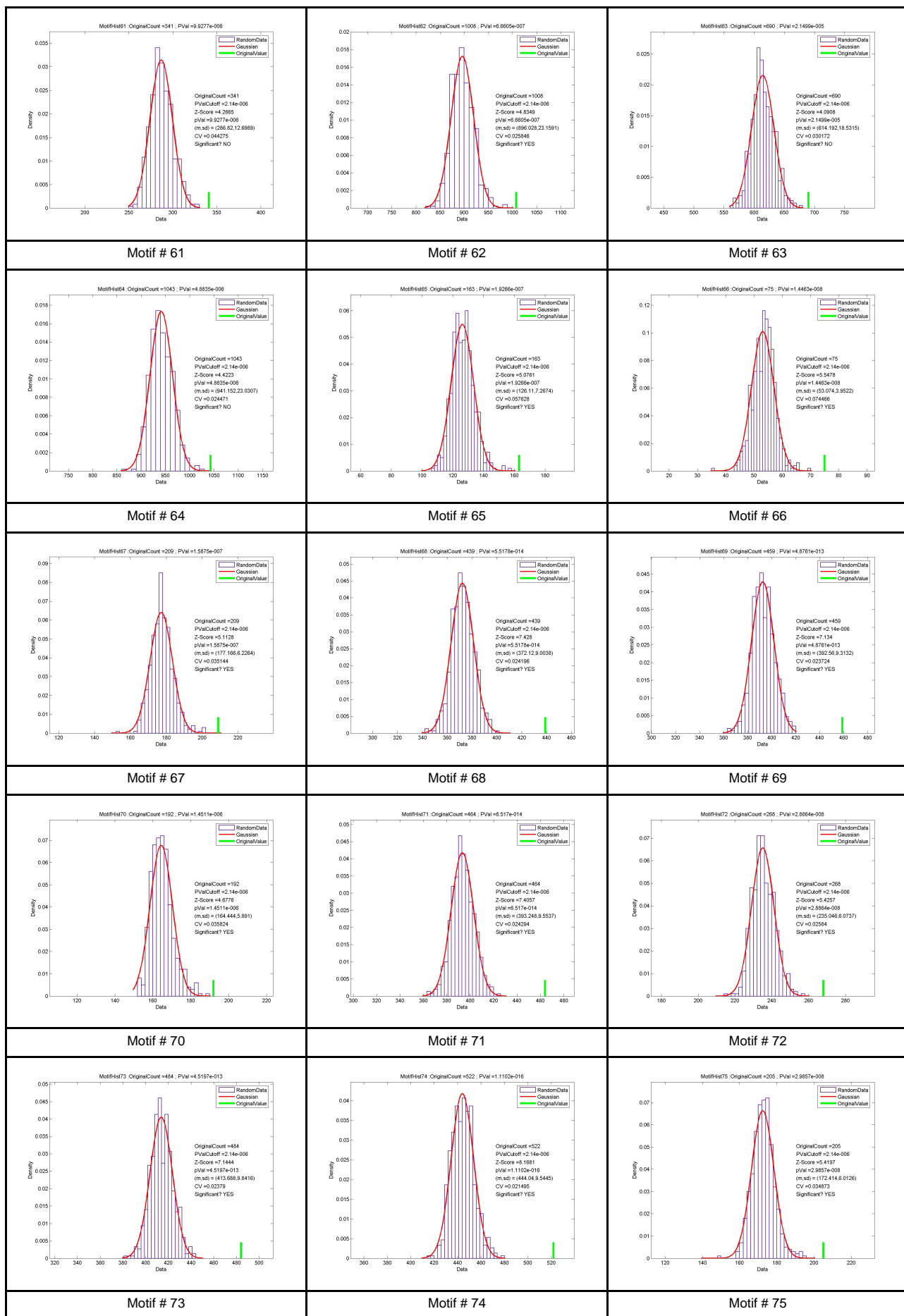

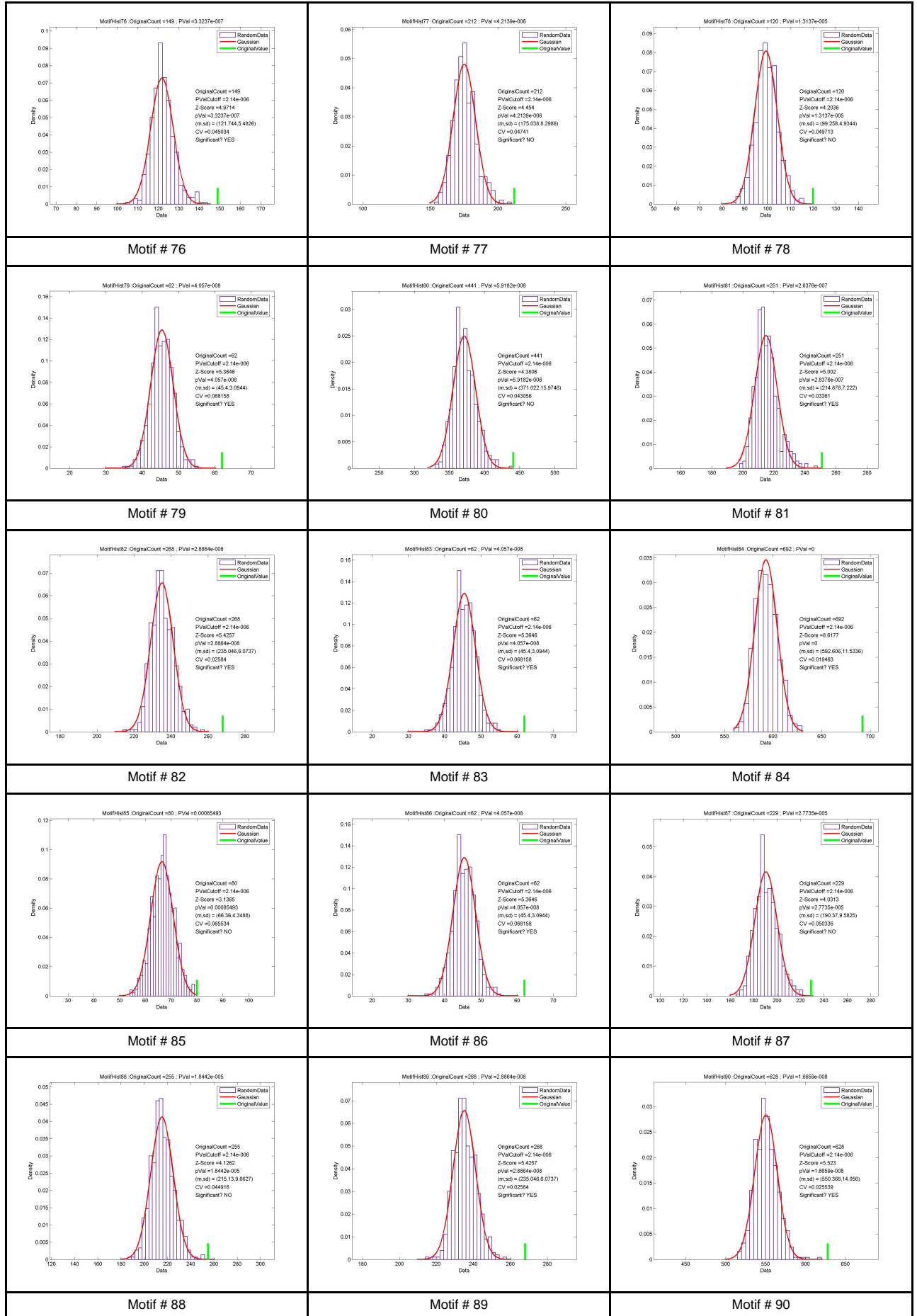

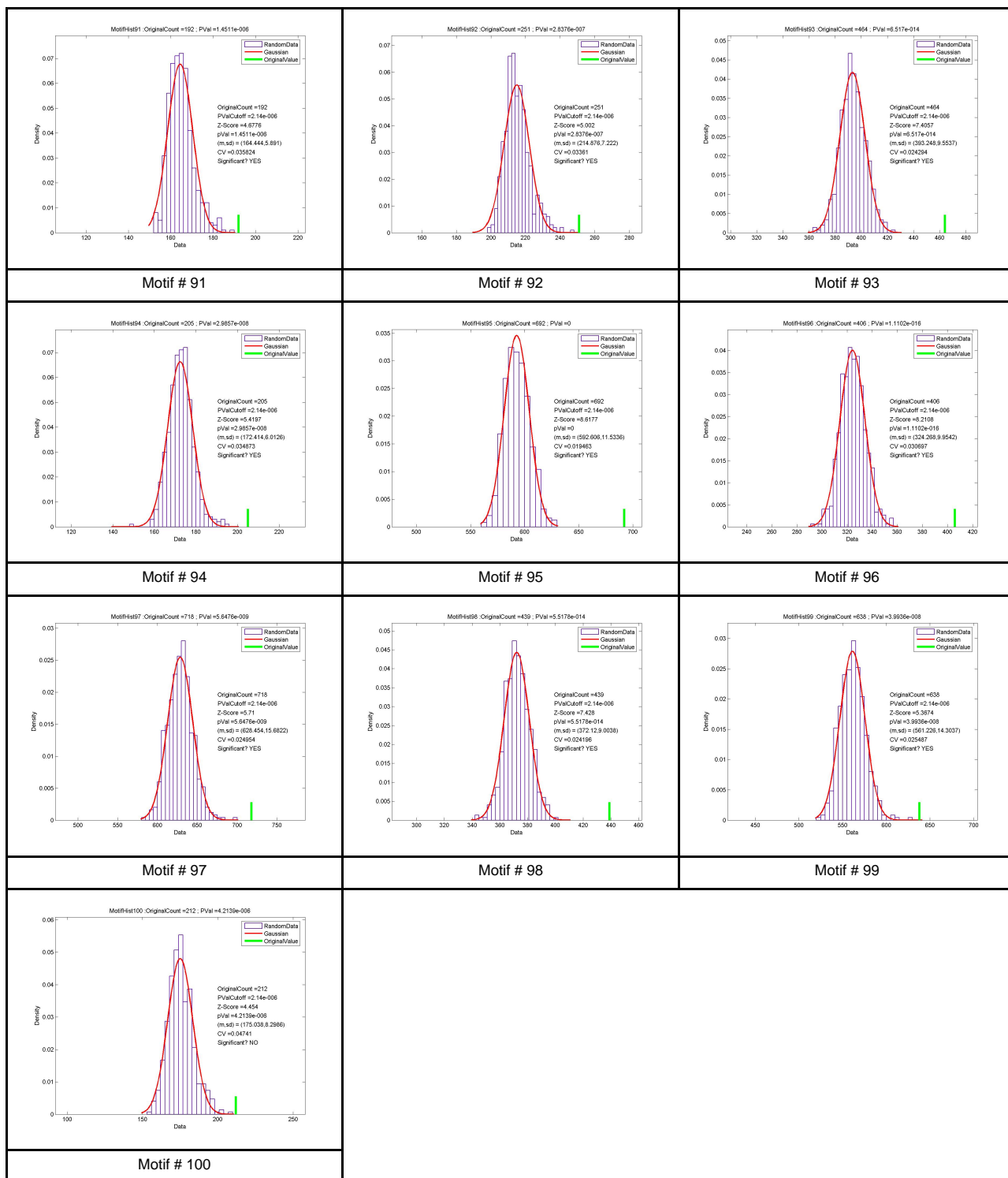

Supplement: Additional data file 20 — Random distribution, parametric fit, and significance of the top 200 significant 3nGO network patterns found in the genetic network. [file gb-2007-8-8-r160-S20.pdf]

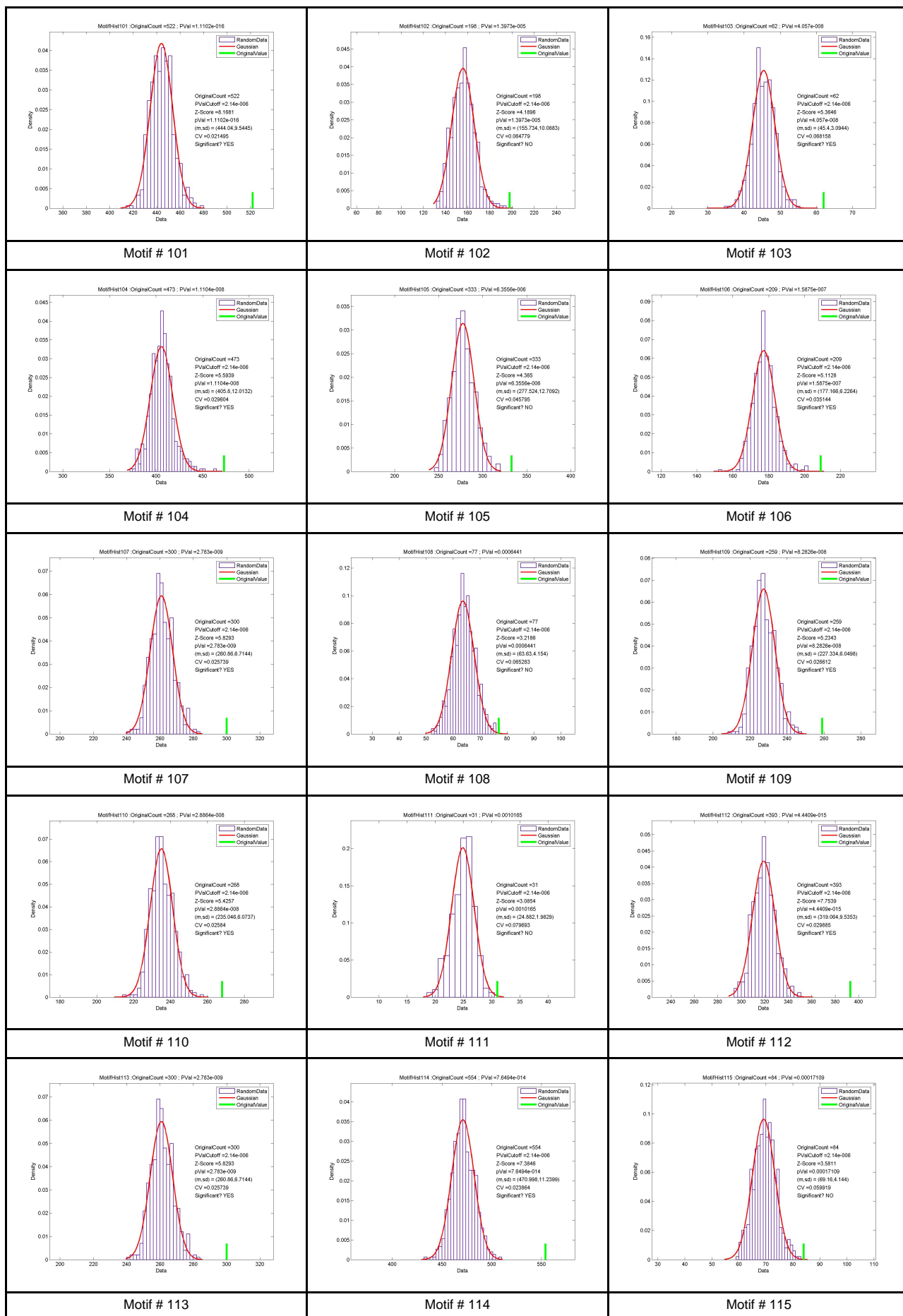

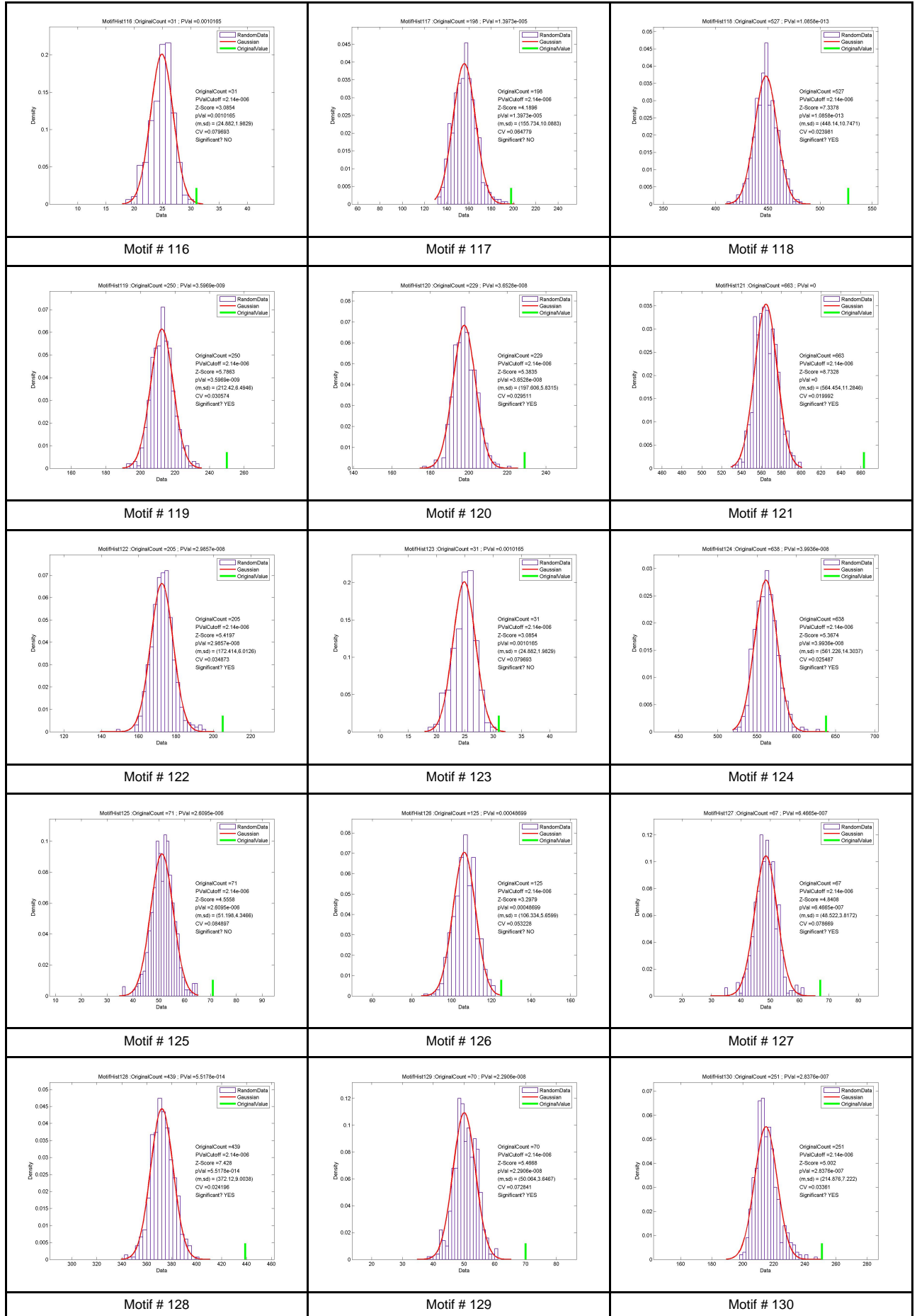

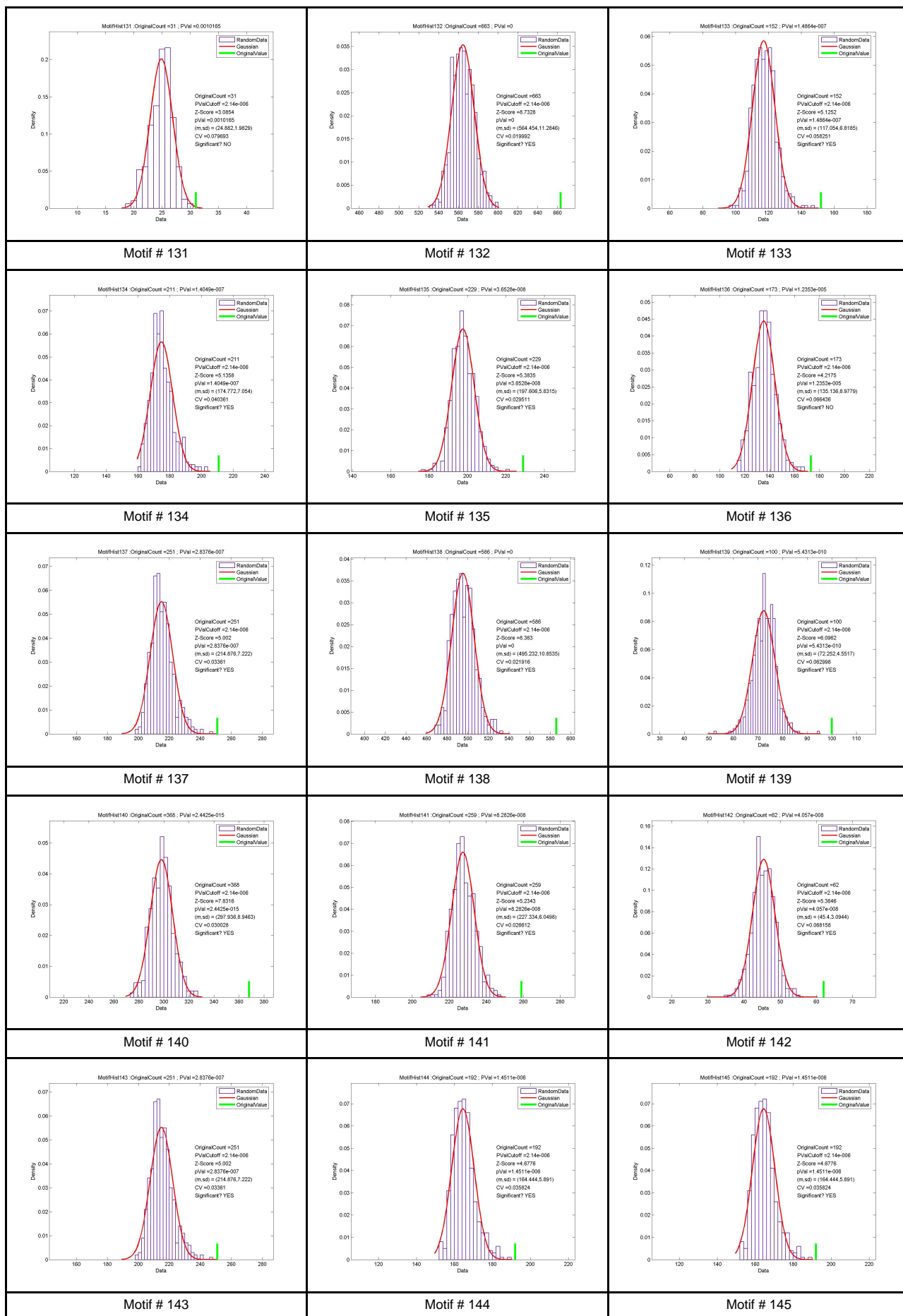

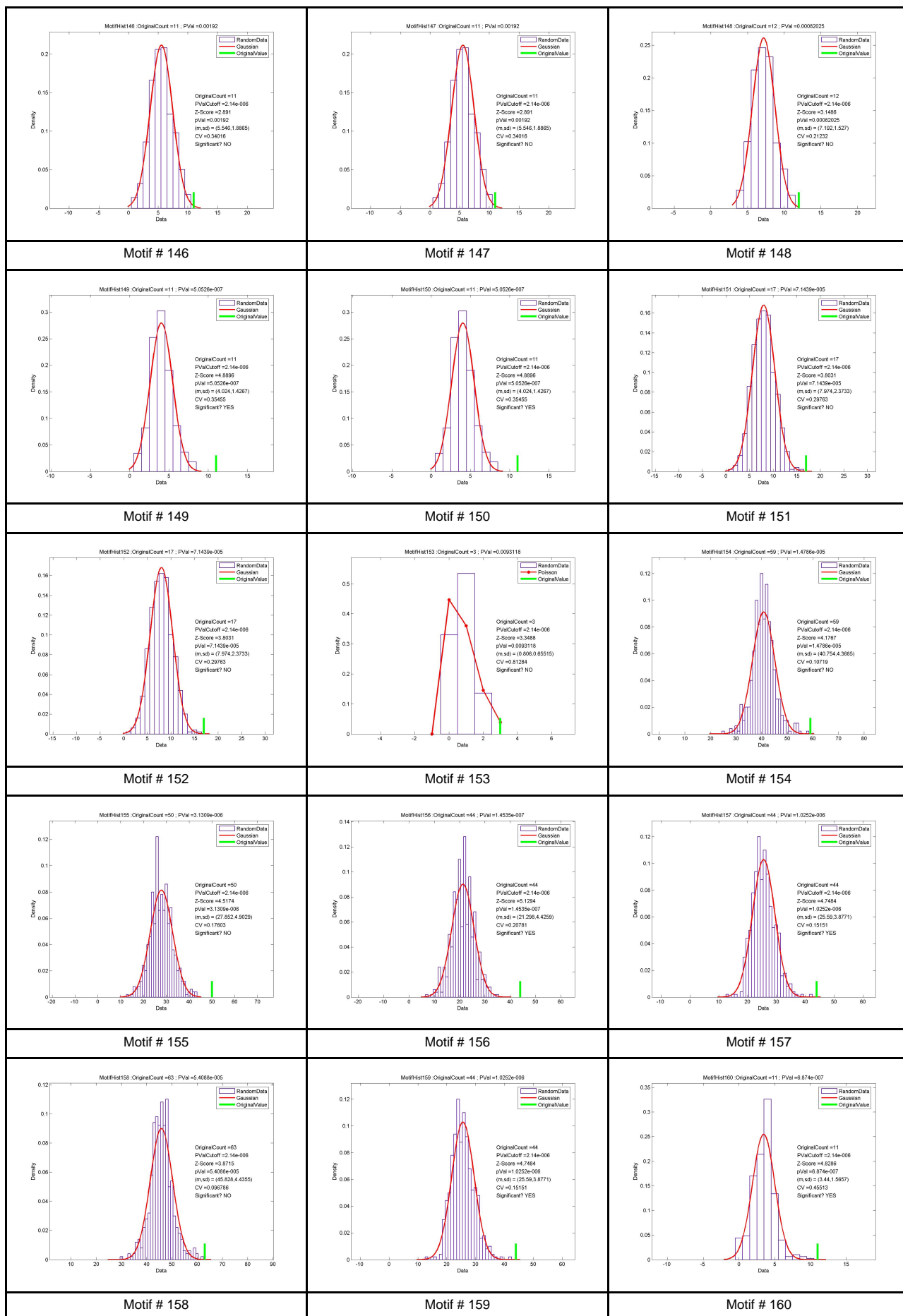

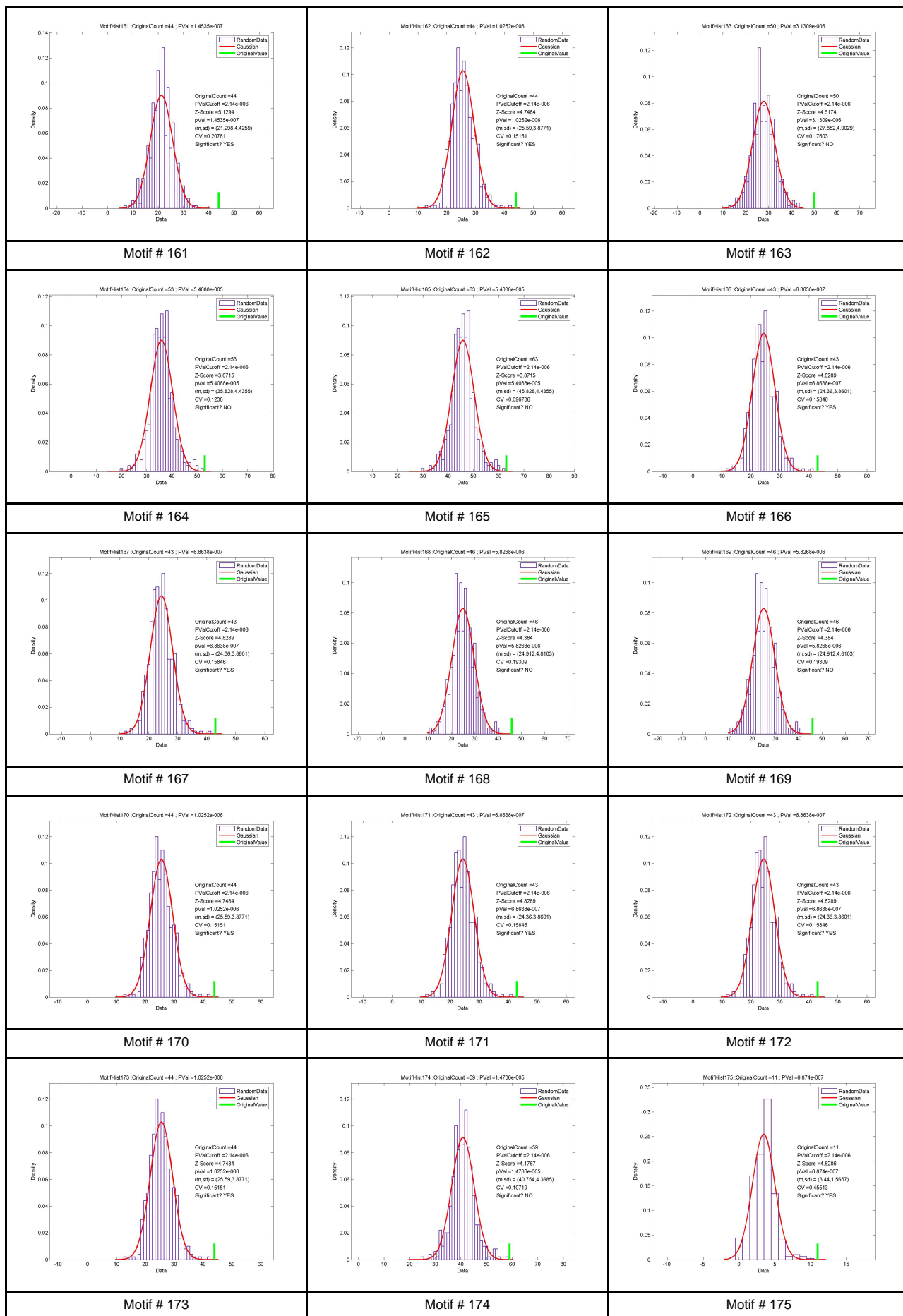

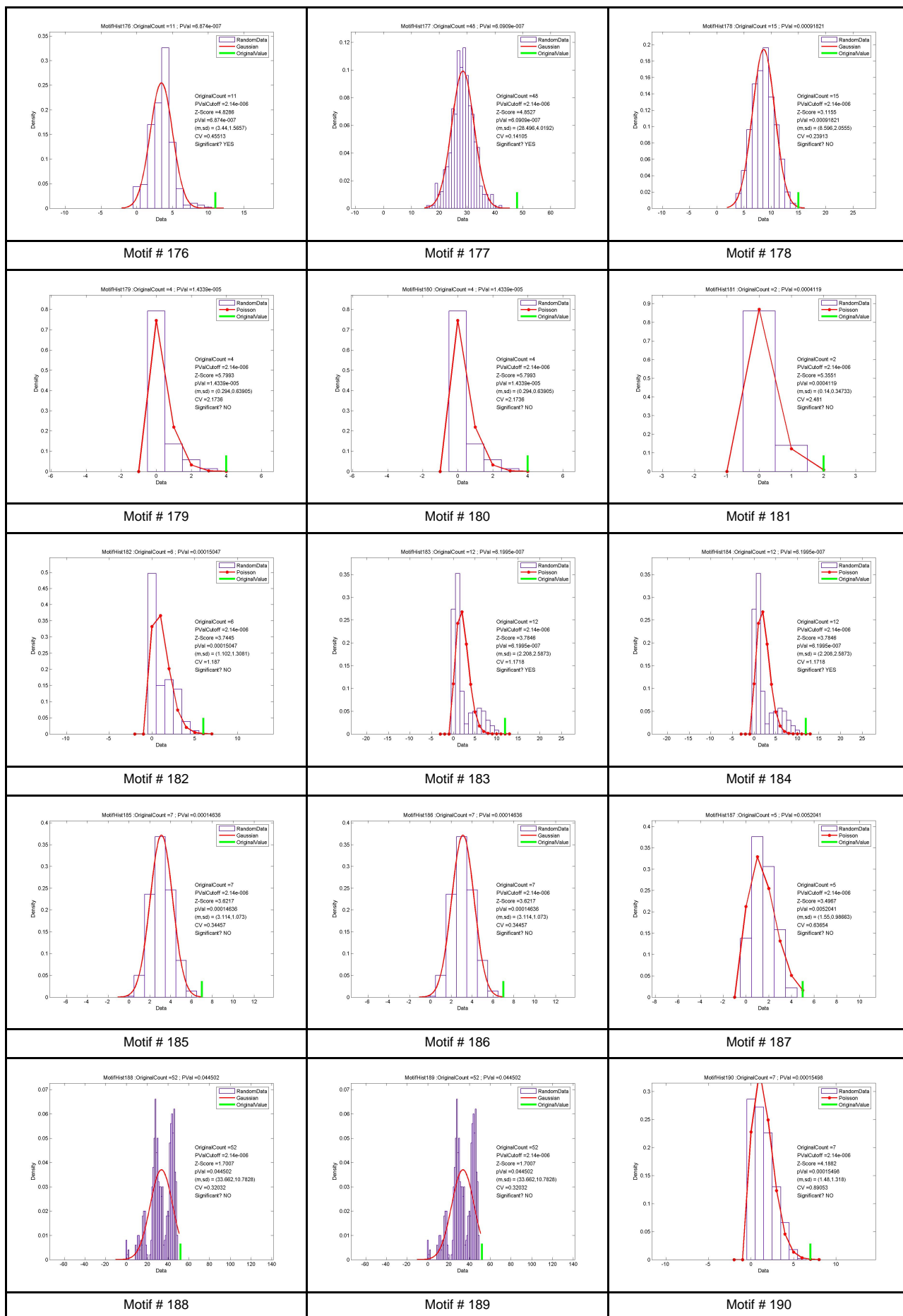

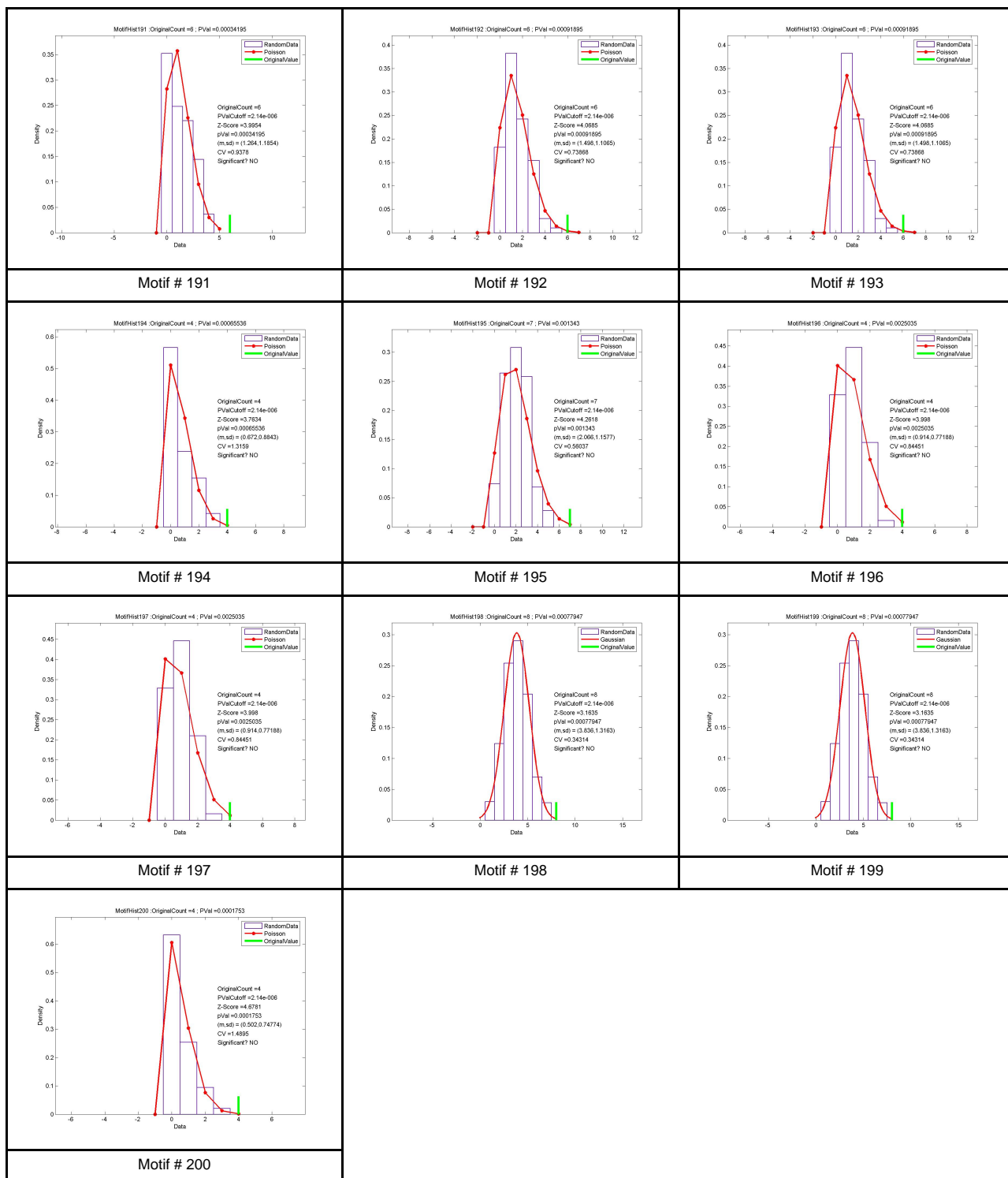

Supplement: Additional data file 21 — Random distribution, parametric fit, and significance of the top 200 significant 3nGO network patterns found in the genetic network. [file gb-2007-8-8-r160-S21.pdf]
