# Supplementary material for: Network motif analysis of a multi-mode genetic-interaction network
Source: Genome Biol. 2007 Aug 2;8(8):R160. doi: 10.1186/gb-2007-8-8-r160 (PMC2374991; doi:10.1186/gb-2007-8-8-r160)
Supplement: Additional data file 10 — Full collection of 3nGO-motifs. [file gb-2007-8-8-r160-S10.pdf]

| | | | | | |
|---|---|---|---|---|---|
| 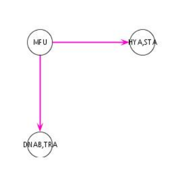 | 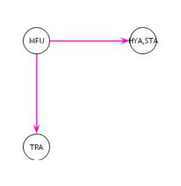 | 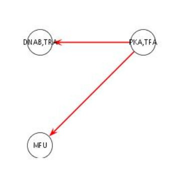 | 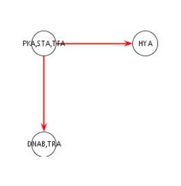 | 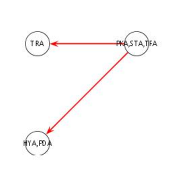 | 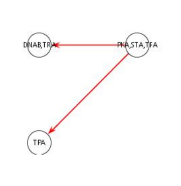 |
| Motif # 149 | Motif # 150 | Motif # 156 | Motif # 157 | Motif # 159 | Motif # 160 |
| Num Real = 11 | Num Real = 11 | Num Real = 44 | Num Real = 44 | Num Real = 44 | Num Real = 11 |
| 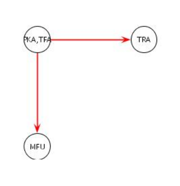 | 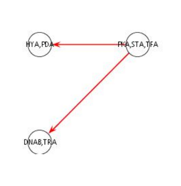 | 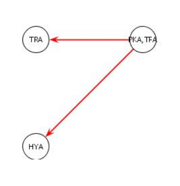 | 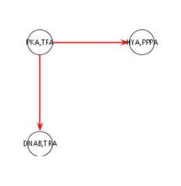 | 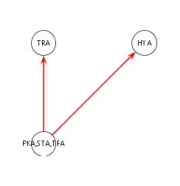 | 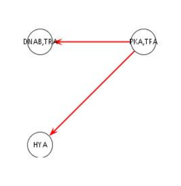 |
| Motif # 161 | Motif # 162 | Motif # 166 | Motif # 167 | Motif # 170 | Motif # 171 |
| Num Real = 44 | Num Real = 44 | Num Real = 43 | Num Real = 43 | Num Real = 44 | Num Real = 43 |
| 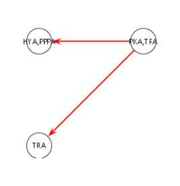 | 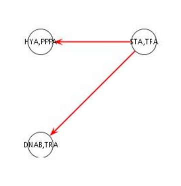 | 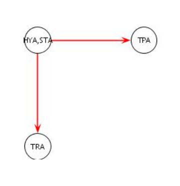 | 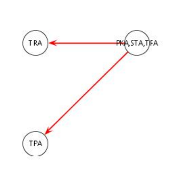 | 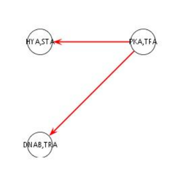 | 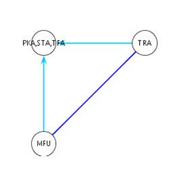 |
| Motif # 172 | Motif # 173 | Motif # 175 | Motif # 176 | Motif # 177 | Motif # 183 |
| Num Real = 43 | Num Real = 44 | Num Real = 11 | Num Real = 11 | Num Real = 48 | Num Real = 12 |
| 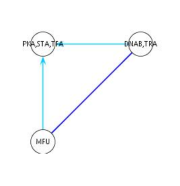 | 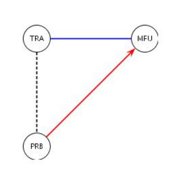 | 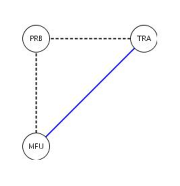 | | | |
| Motif # 184 | Motif # 995 | Motif # 1073 | | | |
| Num Real = 12 | Num Real = 8 | Num Real = 8 | | | |
