# Supplementary material for: Network motif analysis of a multi-mode genetic-interaction network
Source: Genome Biol. 2007 Aug 2;8(8):R160. doi: 10.1186/gb-2007-8-8-r160 (PMC2374991; doi:10.1186/gb-2007-8-8-r160)
Supplement: Additional data file 19 — Software tutorial for the NetworkMotifFinder plugin. [file gb-2007-8-8-r160-S19.doc]

**SOFTWARE TUTORIAL FOR THE CYTOSCAPE PLUGIN**

***NETWORK MOTIF FINDER***

[**Introduction** 2](#__RefHeading___Toc145291714)

[**Running the Network Motif Wizard** 2](#__RefHeading___Toc145291715)

[*Network Motif Wizard Panel 1 – Please Select Edges to Include* 3](#__RefHeading___Toc145291716)

[*Network Motif Wizard Panel 2 – Please Select Node Types to Include* 4](#__RefHeading___Toc145291717)

[*Network Motif Wizard Panel 3 – Please Select the Experimental Parameters* 5](#__RefHeading___Toc145291718)

[*Network Motif Wizard Panel 4 – Please Select Enumeration* 6](#__RefHeading___Toc145291719)

[*Network Motif Finder Panel 5 – Display Options* 7](#__RefHeading___Toc145291720)

[**Running the Null Hypothesis Wizard** 10](#__RefHeading___Toc145291721)

[**Converting Phenotype Genetics Data to a Motif Finder Network** 11](#__RefHeading___Toc145291722)

[**References** 12](#__RefHeading___Toc145291723)

**Introduction**

This tutorial details how to use the *Network Motif Finder* plugin accompanying Taylor et al. 2007. The plugin and all aforementioned data can be downloaded at http://cytoscape.org/plugins2.php. This plugin has been tested on Cytoscape v2.3.

“NetworkMotifFinder.jar” must be loaded into Cytoscape to run the plugin. This can be through the Cytoscape menus Edit-> Preferences…. or by adding the “NetworkMotifFinder.jar” to the core cytoscape plugin directory in your current Cytoscape installment. Cytoscape will then need to be restarted for the Network Pattern Finder plugin to be loaded. All functionality of the *Network Motif Finder* plugin is accessed through the use of the Motif Wizard, which is described in the following.

MEMORY USAGE NOTE: When analyzing networks of many possible motif types (motifs with >3 nodes, or motifs with node attributes) Network Motif Finder uses much memory. If memory issues occur it is recommended to increase the amount of RAM that java accesses, or to reduce the memory usage of the analysis by the ‘*MEMORY TIPS*’ found within the tutorial.

**Running the Network Motif Wizard**

The Motif Wizard guides a user through experiments that identify and apply significance to small repeated network patterns – network motifs. Before running the Motif Wizard, the user will need to:

1. Load the desired network. As these calculations can be very demanding, it is recommended that the smaller network, “2nInvasionSmallExample.sif” is used for this tutorial. This is done through the Cytoscape menus File-> Import -> Network….
2. Load the inequalities edge properties. This can be done through the Cytoscape menus File -> Import -> Edge Attributes… , and loading the file “2nInvasion.inequalities”. If an inequalities file is not loaded, a warning will appear when starting the Wizard. If this occurs, close the wizard, load the inequalities attribute file, and restart the wizard.
3. If motifs that include node attributes (such as GoSlim annotations) are to be identified, the desired node attributes file must be loaded. This can be done through the Cytoscape menus File -> Import -> Node Attributes… , and loading the appropriate file. For this tutorial, load: “GoSlimFunction.gosFunction”.
4. An appropriate Visual Mapper needs to be chosen. A large aspect of the Motif Finder output is an image (.jpg) associated with each motif pattern identified. This image relies on the current Visual Mapper selected in Cytoscape. The mappers automatically loaded by this plugin are: GIMotifFinder and GIMotifFinder_GoSLIM. Use the latter if GoSLIM annotations are incorporated.
5. In case more than one network has been loaded, the network to be analyzed must be selected.

To run the Motif Wizard, click through the Cytoscape menus: Plugins -> Motif Finder Analysis -> Calculate Network Motifs. The Motif Wizard will then be displayed. There are five panels in the Entropy Wizard, with each setting important experimental parameters.

*Network Motif Wizard Panel 1 – Please Select Edges to Include*


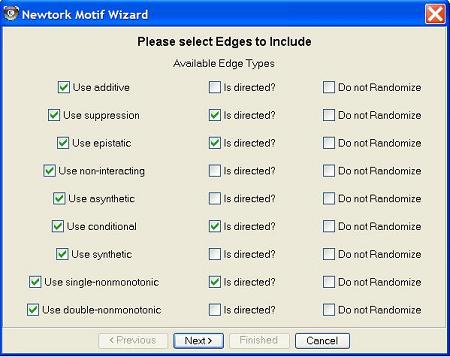


This panel asks the user to choose which edge types are to be included in the analyses. The types are loaded dynamically based on the edge types loaded in Cytoscape. For the analysis the user needs to state:

1. If the edge type should be considered directed. For the edge types of the network used in Taylor et al., the Entropy Wizard automatically selects those which are directed. For other networks, the user needs to select all directed edge types.
2. If any of these edge types should not be randomized in the creation of the null hypothesis, then select “Do not randomize”. In Taylor et al. 2007, all genetic interaction edges are randomized.

*Network Motif Wizard Panel 2 – Please Select Node Types to Include*


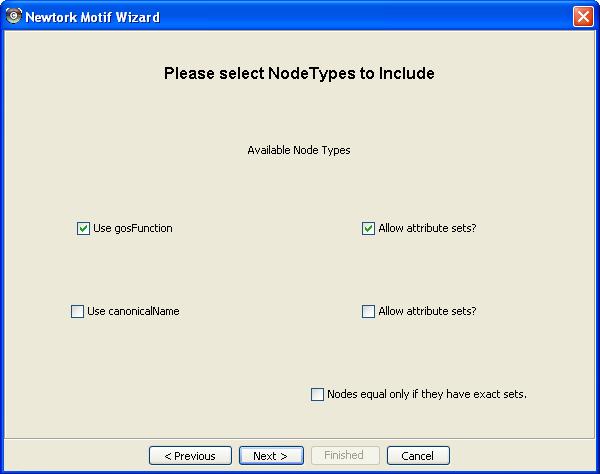


This panel asks the user to choose which node types are to be included in the analyses. The available node types are dynamically loaded into the Motif Wizard, based on the node types loaded into Cytscape. In this example the node attribute file “GoSlimFunction.gosFunction” was loaded into Cytoscape prior to beginning the Motif Wizard, causing the option “Use gosFunction” to appear. The other node types automatically loaded into Cytoscape and are irrelevant to this example.

For the analysis the user needs to state:

1) Whether or not the attribute has been loaded as a set or not. An attribute set means that the node has been loaded with a set of attributes for that type. For example, a single node may have multiple GoSlim Function annotations. Looking in the GoSlimFunction.gosFunction file, the following line can be found:

YER177W = DNAB,PRB

This means that YER177W has been annotated as both a DNA Binding Protein and a Protein Binding Protein. By selecting “Allow Attribute Sets” for the gosFunction attribute, Motif Finder knows that this attribute has a set of values. As shown above, elements of a set need to be separated by a comma.

1. If a node attribute allows attribute sets, the user can specify if “Nodes equal only if have exact sets.” This option is relevant to how motif types are counted during their enumeration. By asserting this option, two node attribute sets are only equal if the two sets share all the exact same elements. If this option is not asserted, attribute sets are considered equal if two sets share a single common element. In Taylor et al. 2007, this option applies to the enumeration of GoSLIM annotation motifs. For the analysis the option was not selected, and motif nodes were considered equal when nodes shared at least a single common set element. See Taylor et al. 2007 for further clarification.

*Network Motif Wizard Panel 3 – Please Select the Experimental Parameters*

**
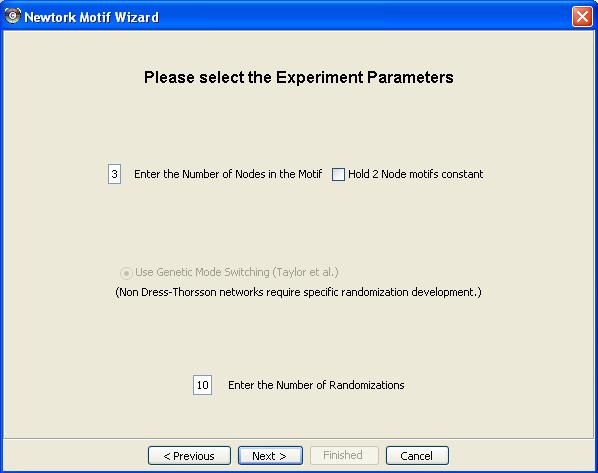
**

In this panel, the user needs to select the following:

1. The number of nodes in the motifs to be found. In Taylor et al., motifs containing exactly 2 nodes, 3 nodes and 4 nodes were found.
2. Whether or not to hold the number of 2 node motifs constant during randomizations. In Taylor et al, the number of 2node motifs was held constant during the enumeration of 3node GOSlim annotation motifs.
3. What network randomization calculator to use. As seen, there is currently only the single calculator, “Genetic Mode Switching (Taylor et al)” available. The *Motif Network Finder* was built such that users can easily develop and insert newly coded randomization procedures for new network types. Example network types may be molecular interaction networks containing protein-protein and protein-DNA interactions, or genetic networks extended with new molecular interaction data types.
4. How many randomizations of the original network will be used to achieve statistical significance.

*Network Motif Wizard Panel 4 – Please Select Enumeration*


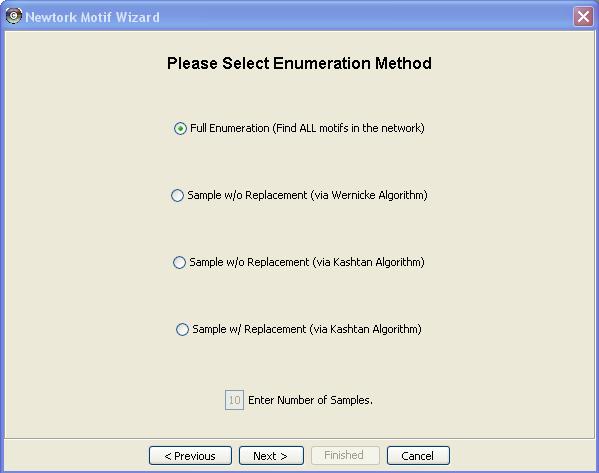


This panel asks the user to define what enumeration method is to be used to count the motifs. The options are:

1. Full enumeration – This counts through every possible motif in the network.
2. Sample (via Wernicke Algorithm) – This is a sampling algorithm based on the Wernicke network motif sampling algorithm (Wernicke 2005). This sampling algorithm was used in Taylor et al. as it was found to be computationally the fastest. This instance of the algorithm samples without replacement.
3. Sample without replacement (via Kashtan Algorithm) – This is a sampling algorithm based on the Kashtan network motif sampling algorithm (Kashtan, Itzkovitz et al. 2004). This instance of the algorithm samples without replacement.
4. Sample with replacement – This is a sampling algorithm based on the Kashtan network motif sampling algorithm (Kashtan, Itzkovitz et al. 2004). This instance of the algorithm samples without replacement.

If a sampling algorithm is chosen then the number of motifs to be sampled needs to be assigned.

*Network Motif Finder Panel 5 – Display Options*


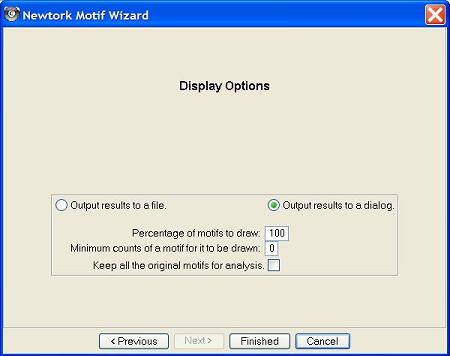


This panel allows the user to specify if they would like to save the results straight to files or to open them in a dialog table.

***Saving to a file.*** If the results are saved to a file, the user will be prompted with a save dialog box. After the analysis has completed the following files are saved:

1. *.xml file that contains all of the statistical and motif information for each motif type.
2. *-logger.txt file that contains the relevant analysis parameters as well as some general information about each network enumeration and randomization.
3. *.jpg files that are images of all motif types that were found in the original network. Images are saved along with the raw data as it is much easier to understand motifs visually.

***Saving to a dialog box.*** If the results are to be saved to a dialog, the following options are available:

1. Percentage of motifs to draw. This allows the user to only display the most and least significant x/2% motifs in the display dialog.
2. Minimum counts of a motif for it to be drawn. This allows user to specify the number of counts a motif has before it is displayed in the dialog.
3. Keep all the original motifs for the analysis. As discussed below, the display dialog has functionality to reintegrate the identified motifs back into Cytoscape. This option needs to be selected if these functionalities are to be used.

Once these options are set, the analysis is run and the following dialog appears upon completion.


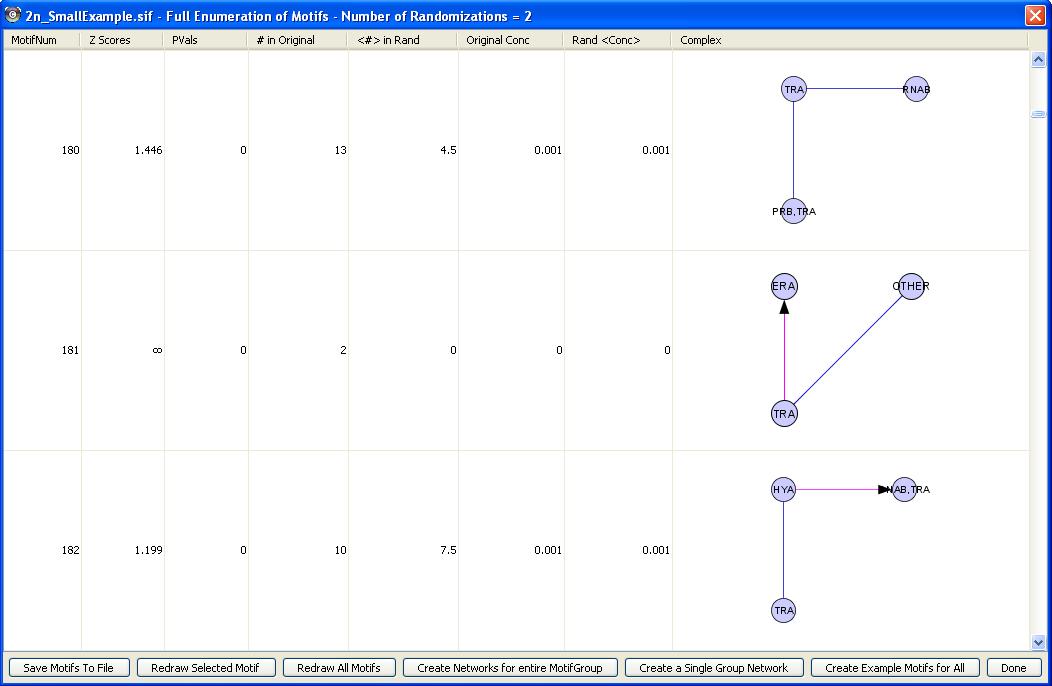


The dialog displays the statistical information about each motif type as well as an example image. Using the buttons along the bottom of the dialog, the user has the ability to:

1. *Save Motifs to File*. This saves the same files as the “Save to file” option above.
2. *Redraw Selected Motif*. This redraws the image of a selected motif type. A motif type is selected by clicking it.
3. *Redraw All Motifs.* This feature redraws all motif type images. This feature is usually used if an incorrect Visual Mapper was selected when the analysis was initiated. With this, all images can be updated after a new Visual Mapper is selected.
4. *Create Networks for entire Motif Group*. This feature can be used to create individual Cytoscape networks for all of the motifs found in the original network that make up the selected motif type. The ‘Keep all the original motifs for the analysis’ option needs to be selected in the display options as discussed above.
5. *Create a Single Group Network*. This takes all the enumerated motifs of a type and creates a single Motif subnetwork as discussed in Taylor et al. 2007. The ‘Keep all the original motifs for the analysis’ option needs to be selected in the display options as discussed above.
6. *Create Example Motifs for All*. This feature creates a Cytoscape network of a representative motif for each motif type. These motifs are automatically loaded into Cytoscape where they can be further analyzed and manipulated.
7. *Done*. Close the dialog. All analysis is discarded at this point.

*MEMORY TIP: If analyzing networks of many motif types, the images displayed in the dialog can use much memory. Use the ‘Save to a file’ option above if java runs out of memory after the final random network is analyzed, and before the dialog is displayed.*

**Running the Null Hypothesis Wizard**

The Null Hypothesis Wizard can be run through Plugins -> Motif Finder Analysis -> Compare Null Hypotheses… Running the Null Hypothesis Wizard is similar to running the Network Motif Finder Wizard, with the below exceptions. We refer the user to the above sections for a full tutorial. Differences to the Network Motif Finder Wizard:

1) The user has to manually define which two networks are to be compared. When beginning the Null Hypothesis Wizard an initial dialog prompts the user for to define these networks. This is done by selected the desired network and clicking on either Network1 or Network2 as shown below:


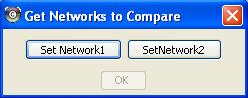


2) Once both networks are chosen the Null Hypothesis Wizard is displayed. This Wizard is the same as the Network Motif Finder Wizard, so please refer to the above sections for a tutorial on the Wizard. The only exception is that the Null Hypothesis Wizard has no display options. This is because all Null Hypothesis data is saved in the Output Logger that runs during the analysis. The relevant analysis parameters as well as some general information about each network enumeration and randomization can be found here. As well the correlation value comparing the two Null Hypotheses can be found at the end of the log, and all data can be saved through the ‘Save Log’ button on the logger dialog.

**Converting Phenotype Genetics Data to a Motif Finder Network**

The following is an exception of how to convert Phenotype Genetics data into a network readable for the MotifFinder pluging. It is assumed that the user is familiar with the Phenotype Genetics Cytoscape plugin. This is necessary as the MotifFinder plugin algorithms assume that all directed inequalities have an A->B perturbation directionality, while Phenotype Genetics also allows for a B->A perturbation directionality. More information can be found at <http://cytoscape.org/plugins2.php>. To convert Phenotype Genetics data, take the following steps:

1. Using the Phenotype Genetics Cytoscape plugin, load in the appropriate data file (*.xml). Shown here is 2n_SLAD_inv.xml file used for the analysis in Taylor et al.


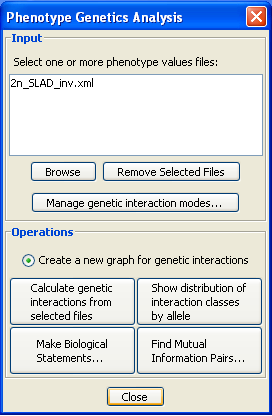


The network is loaded into Cytoscape using the “Calculate genetic interactions from selected files” button. It should be noted that the user may first need to define the possible edge types by using the “Manage genetic interaction modes…” and loading the appropriate *.xml definition file. In our case the “phenGenModes.xml” definition file is loaded.

1. Select the edges to be exported into a new network for the Motif Finder Analysis. Either all edges can be selected by using the Cytoscape menu: Select -> Select all Nodes and Edges, or a subset of edges can be selected using mouse drags or the Cytoscape filters.
2. Export the Phenotype Genetics data. This is done by using the Cytoscape menu: File -> Export -> Edge Attributes. A dialog will be displayed with the possible attributes to save. Click the “phenotype inequality” attribute, and then the ‘Choose Directory and Save’.

In the *Network Motif Finder* plugin, tools have been created to convert this phenotype attributes file into a Cytoscape network that *Network Motif Finder* can use.

1. Convert the Phenotype Genetics attribute file into a MotifFinder network using the MotifFinderTools. This is done by using the Cytoscape Menu: Plugins -> Motif Finder Tools -> Parse Phenotype Genetics File. When requested, open up the saved “phenotype inequality” file described above. This will save a new *.sif file and *.inequalities attribute file to the same directory (the file names will have the suffix “_NewEdges”).
2. Load the new *.sif file and *.inequalities file into Cytoscape. This network can now be analyzed using the *Network Motif Finder* plugin. As in the 2n_SLAD_inv.xml derived network, there may be double edges between a pair of nodes (due to multiple genetic interaction experiments of different gene alleles). These double edges can be pruned from a network by using the tool: Plugins -> Motif Finder Tools -> Prune a Network’s Double Edges. This adds an additional pruned network in Cytoscape that can be saved and analyzed with the *Network Motif Finder* plugin.

**References**

Kashtan, N., S. Itzkovitz, et al. (2004). "Efficient sampling algorithm for estimating subgraph concentrations and detecting network motifs." Bioinformatics 20(11): 1746-58.

Wernicke, S. (2005). A faster algorithm for detecting network motifs. Proceedings of the 5th Workshop on Algorithms in Bioinformatics (WABI '05).
