# Supplementary material for: Network motif analysis of a multi-mode genetic-interaction network
Source: Genome Biol. 2007 Aug 2;8(8):R160. doi: 10.1186/gb-2007-8-8-r160 (PMC2374991; doi:10.1186/gb-2007-8-8-r160)
Supplement: Additional data file 22 — Supplemental Figure 1: definition of the genetic interactions used in this study. [file gb-2007-8-8-r160-S22.pdf]

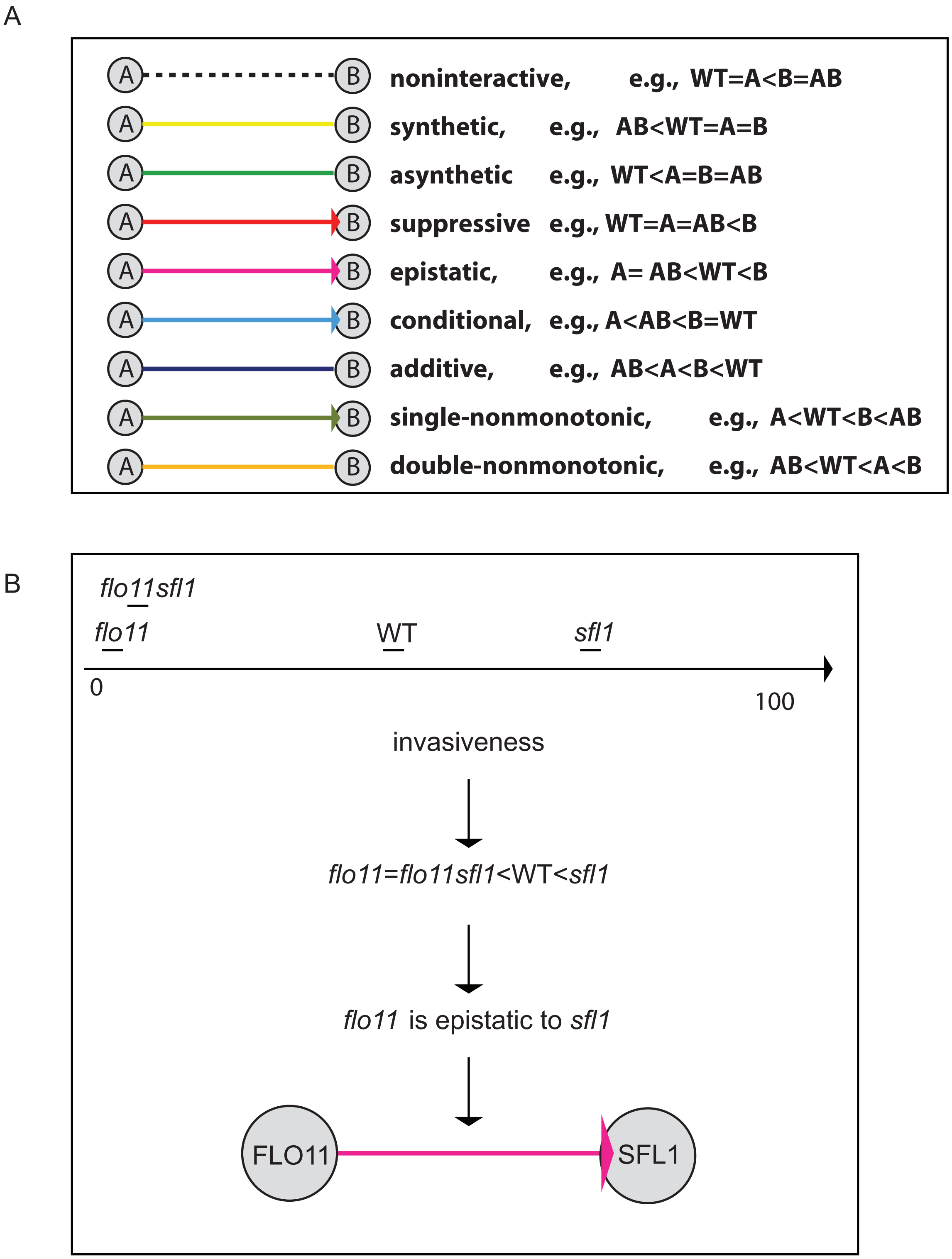

Supplemental Figure 1. Defining genetic interactions.
A) An example of the invasion-phenotype measurements involved in a genetic interaction between deletions of the FLO11 and SFL1 genes: (WT, *flo11*, *slf1*, *flo11 sfl1*). The data can be written as an inequality. There are 75 permutations of the 4 measurements.
B) In Drees et al. [1], the 75 inequalities were grouped into 9 genetic-interaction modes. Here each mode is shown with an example inequality.
