# Supplementary material for: Network motif analysis of a multi-mode genetic-interaction network
Source: Genome Biol. 2007 Aug 2;8(8):R160. doi: 10.1186/gb-2007-8-8-r160 (PMC2374991; doi:10.1186/gb-2007-8-8-r160)
Supplement: Additional data file 23 — Supplemental Figure 2a,b: further examples of 3n-motif subnetworks. [file gb-2007-8-8-r160-S23.pdf]

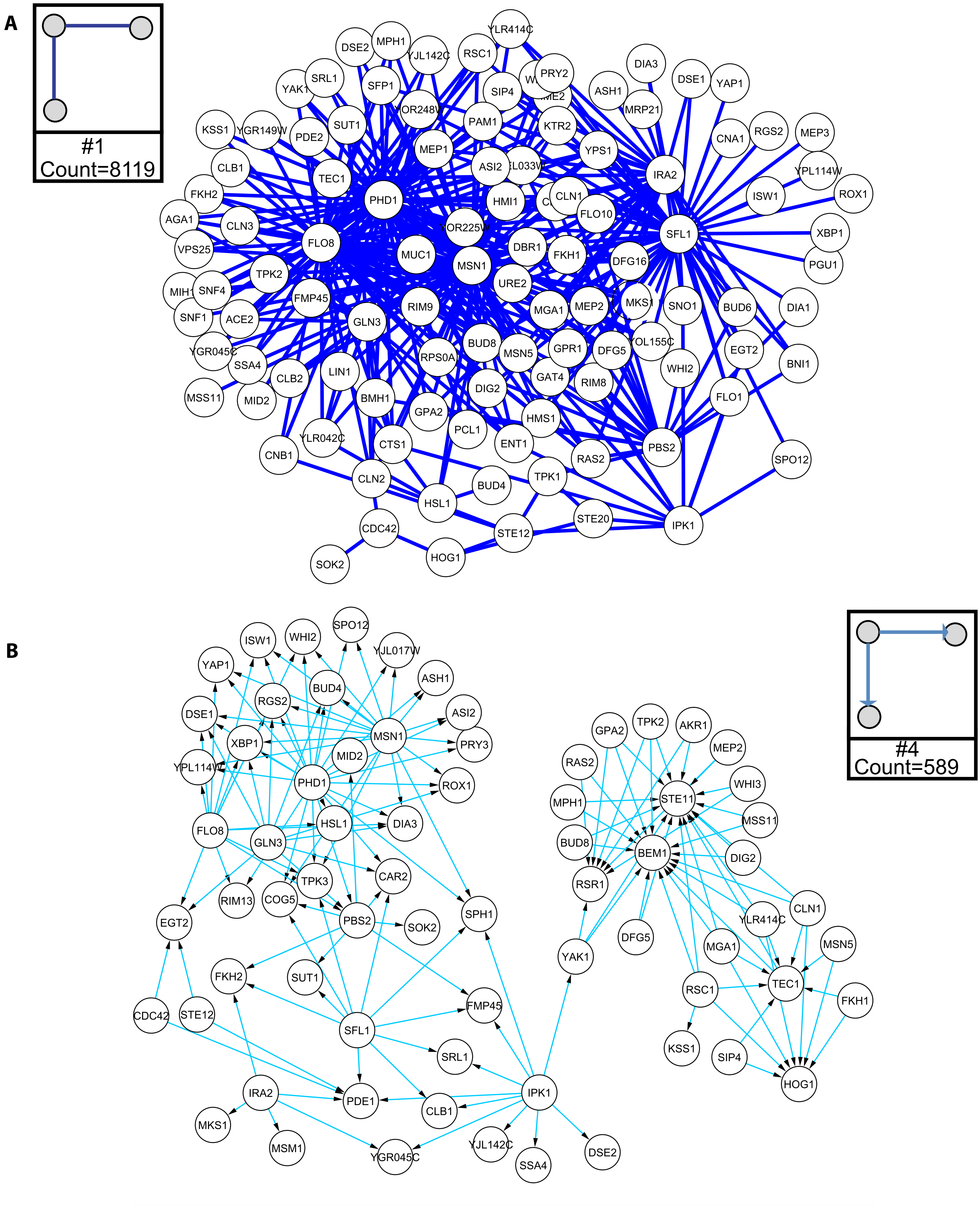

Supplemental Figure 2. Further examples of motif subnetworks.
A) The subnetwork of an additive motif, 3n-motif 1, forms a dense interaction network centered around the genes TEC1, PHD1, MUC1, SFL1 and FLO8.
B) The subnetwork of the outgoing conditional motif, 3n-motif 4, occurs in two main clusters showing opposite hub directionality.
