# Supplementary material for: Network motif analysis of a multi-mode genetic-interaction network
Source: Genome Biol. 2007 Aug 2;8(8):R160. doi: 10.1186/gb-2007-8-8-r160 (PMC2374991; doi:10.1186/gb-2007-8-8-r160)
Supplement: Additional data file 24 — Supplemental Figure 2c,d: further examples of 3n-motif subnetworks. [file gb-2007-8-8-r160-S24.pdf]

C

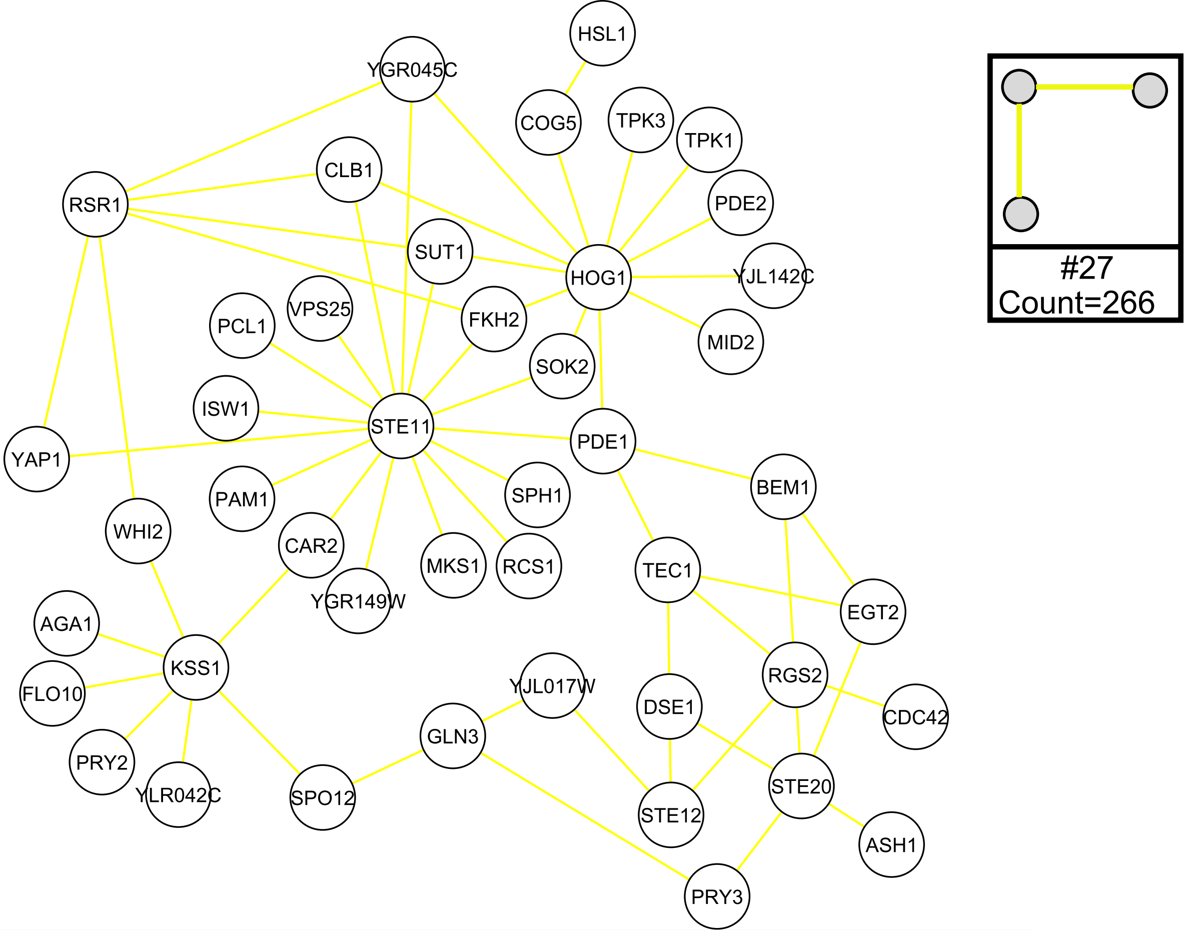

D

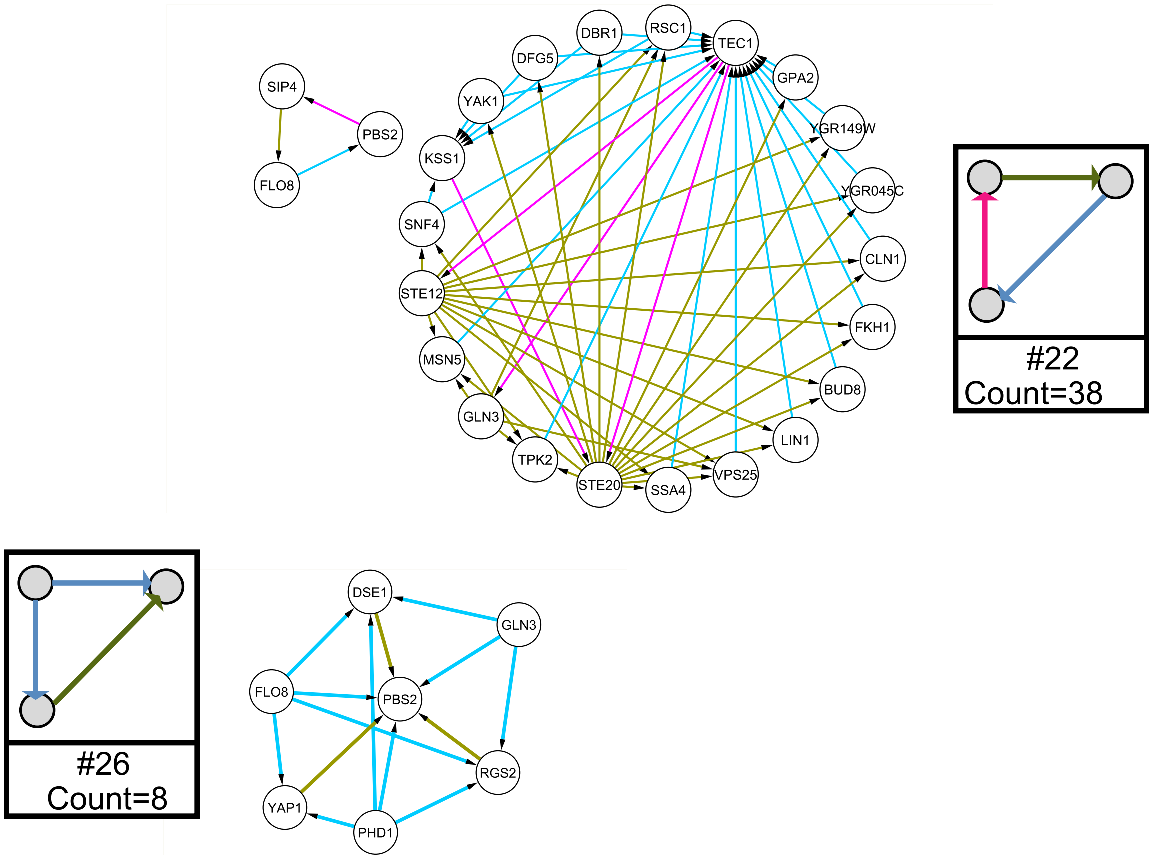

Supplemental Figure 2. Further examples of motif subnetworks, continued.
C) The synthetic interaction motif, 3n-motif 27, forms a subnetwork of relatively low density.
D) Motifs 3n-motif 22 and 26 form multimode subnetworks dominated by single genes, TEC1 and PBS2, respectively.
