# Supplementary material for: Network motif analysis of a multi-mode genetic-interaction network
Source: Genome Biol. 2007 Aug 2;8(8):R160. doi: 10.1186/gb-2007-8-8-r160 (PMC2374991; doi:10.1186/gb-2007-8-8-r160)
Supplement: Additional data file 25 — Supplemental Figure 2e: further examples of 4n-motif subnetworks. [file gb-2007-8-8-r160-S25.pdf]

E

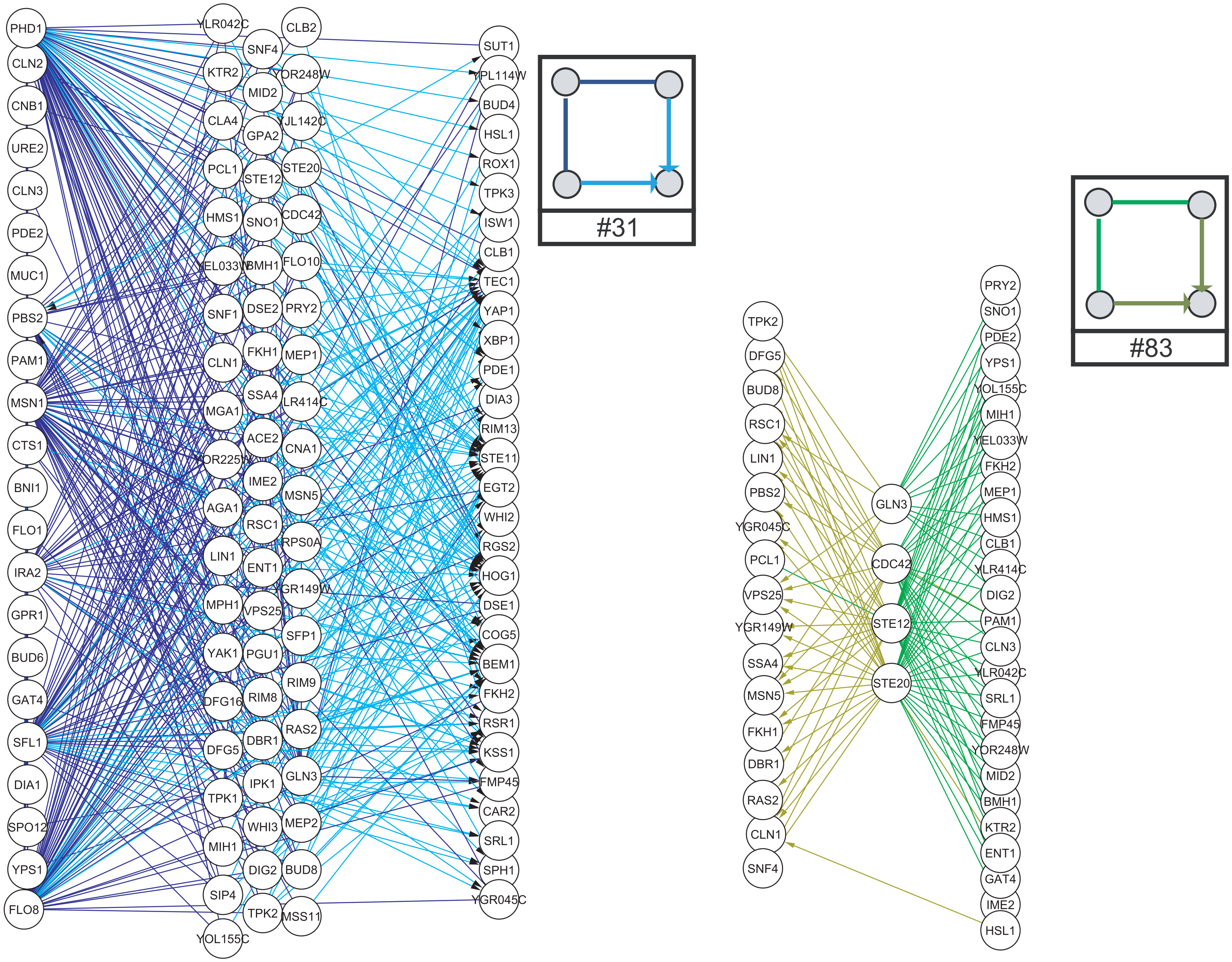

Supplemental Figure 2. Further examples of motif subnetworks, continued.
E) Motif subnetworks of 4n-motifs 31 and 83 illustrate common interaction modes with numerous common interaction partners.
